# Novel 3D models for β-catenin-driven liver cancers

**DOI:** 10.1016/j.jhepr.2026.102040

**Published:** 2026-09-18

**Authors:** Vanessa Bou Malham, Fanny Léandre, Akila Hamimi, Isabelle Lagoutte, Sandrine Bouchet, Alexandre Archambault-Marsan, Jérémy Augustin, Angélique Gougelet, Sabine Colnot, Christèle Desbois-Mouthon

**Affiliations:** 1 INSERM, Université Paris Cité, Sorbonne Université, Centre de Recherche des Cordeliers, F-75006 Paris, France; 2 Team « Oncogenic functions of beta-catenin signaling in the liver », Équipe labellisée par la Ligue Nationale contre le Cancer, F-75013 Paris, France; 3 AP-HP, Institut du Cancer Paris CARPEM, F-75015 Paris, France; 4 Projet de recherche Enfants, Adolescents et Cancer Ligue Contre le Cancer, France; 5 INSERM, CNRS, Université Paris Cité, Institut Cochin, Plateforme Imagerie du vivant, F-75014 Paris, France; 6 AP-HP, Hôpital Saint-Antoine, F-75012 Paris, France; 7 AP-HP, Hôpital Henri Mondor, Département d’anatomopathologie, F-94000 Créteil, France; 8 Faculté de médecine, Université de Montréal, Département de pathologie et biologie cellulaire, Montréal, Québec, Canada; 9 INSERM, Institut Mondor de Recherche Biomédicale, F-94000 Créteil, France

**Keywords:** Tumouroids, 3D culture, rotating bioreactor, preclinical modeling, tumour microenvironment, therapeutic screening, translational research

## Abstract

**Background & Aims:**

Constitutive activation of β-catenin is a key feature in the pathogenesis of hepatocellular carcinoma (HCC) and hepatoblastoma (HB). Activating alterations in the *CTNNB1* gene and, to a lesser extent, inactivating alterations in the *APC* gene are observed in 30-40% of HCC cases and 80-90% of HB cases. For both tumours, therapeutic options remain suboptimal. Therefore, relevant experimental models are needed to improve our understanding and to test new therapeutic approaches.

**Methods:**

Liver organoids (n = 17 mice) and tumouroids (n = 14 mice) were established from APC^Δhep^ and βcat^Δex3^ mouse models, which are clinically relevant models of β-catenin-activated HCC and mesenchymal HB. We developed a new methodological approach based on dynamic suspension culture in a rotating bioreactor. Morphological and molecular characteristics, as well as drug responses, were evaluated by histology, immunohistochemistry, immunofluorescence, transcriptomics and reverse-transcription quantitative PCR.

**Results:**

This easy-to-implement methodology allows for the rapid production of large numbers of uniform organoids and tumouroids (30-75 per tumour) without detectable cell death and hypoxia in their core. The robustness of the approach is also illustrated by the preservation of histological architecture, cellular diversity (tumour cells, macrophages, and endothelial cells) and gene expression in organoids and tumouroids compared with native liver tissue. Gene set enrichment analysis showed significant enrichment of 10 human HCC/HB tumour signatures in murine tumouroids. Organoids and tumouroids showed the expected responses to specific inhibitors, including the β-catenin antagonist WNTinib.

**Conclusions:**

These organoids and tumouroids represent robust *in vitro* models of β-catenin-driven HCC and mesenchymal HB. They provide a scalable platform for drug screening and may support the development of “à la carte” therapies urgently needed for these indications.

**Impact and implications:**

This study addresses the need for representative *in vitro* models to investigate β-catenin-driven liver cancers. The tumouroids developed here provide robust and reproducible 3D models that closely recapitulate the histological features, cellular diversity, and gene expression profiles of native liver tumours. These models offer a valuable platform for studying tumour biology, evaluating therapeutic candidates, and supporting the development of personalised treatment strategies. They may also contribute to reducing the use of animals in preclinical research.

## Highlights

• Easy and rapid generation of mouse organoids and tumouroids from β-catenin-activated livers and tumours using a rotating bioreactor culture system.
• 3D models preserve the histological architecture, cellular diversity, and gene expression profiles of the corresponding native liver tissue.
• 3D models exhibit expected responses to several drugs, including the β-catenin inhibitor WNTinib.
• These robust 3D models provide a valuable platform for drug screening while reducing reliance on animal experimentation.

## Graphical abstract

**Figure:**
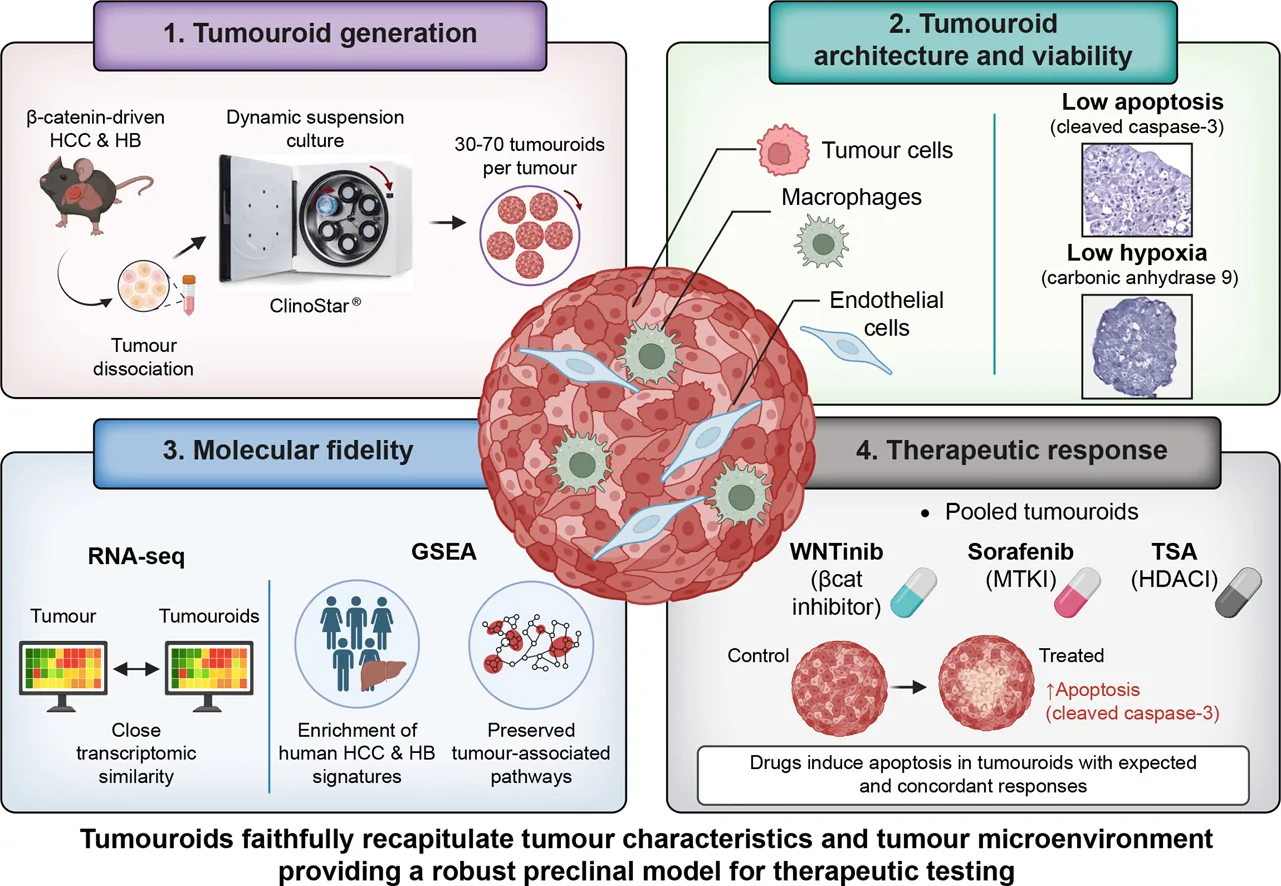

## Introduction

Oncogenic activation of β-catenin is a key driver of primary liver cancers in both adults and children, namely hepatocellular carcinoma (HCC) and hepatoblastoma (HB), respectively. HCC is the most common primary liver malignancy, with more than 900,000 new cases diagnosed worldwide annually, and represents the third leading cause of cancer-related death, accounting for more than 830,000 deaths in 2020.^1^ HB is the most common primary liver cancer in children, typically diagnosed before five years of age, with an estimated incidence of 1-1.5 cases per million children and a marked increase over recent decades.^2^

Recurrent genetic alterations affecting *CTNNB1*, encoding β-catenin, are frequently observed in primary liver cancers. Gain-of-function mutations in *CTNNB1* occur in 30-40% of HCC, whereas gain-of-function deletions are detected in approximately 80% of HB. Loss-of-function deletions in *APC,* a tumour-suppressor gene encoding a key negative regulator of the β-catenin pathway, have also been reported in approximately 3% of HCC and 10% of HB cases.^3^^,^^4^ These alterations induce β-catenin stabilisation and nuclear accumulation, enabling β-catenin to interact with the transcription factor TCF-4 and activate transcriptional programmes involved in tumour initiation and progression. β-catenin pathway-altered HCC represents a relatively homogenous subgroup of well-differentiated tumours with low proliferative activity. Although associated with favourable outcomes when detected early and treated surgically,[3], [4] no specific treatment is currently available for advanced *CTNNB1*-mutated HCC, and oncogenic β-catenin activation has been linked to resistance to immune checkpoint inhibitor therapies.[5], [6], [7], [8] HB exhibits marked phenotypic diversity, with three major histological subtypes spanning a spectrum of differentiation states^9^ and reflecting distinct transcriptomic profiles.[10], [11], [12], [13] Current treatment relies on cisplatin-based chemotherapy combined with surgical resection, resulting in 5-year survival rates exceeding 80%.^14^ However, unresectable, chemoresistant or recurrent tumours are associated with poor outcomes, and chemotherapy-associated toxicity remains a major clinical concern.

For many years, our team has generated and characterised mouse models of β-catenin-driven liver carcinogenesis. Using Cre-lox recombination and, more recently, CRISPR/Cas9-based approaches, we demonstrated that oncogenic activation of β-catenin signalling in hepatocytes, through loss of *Apc* expression (APC^Δhep^ model) or stabilising mutations in *Ctnnb1* (βcat^Δex3^ model), is sufficient to induce transformation and tumour initiation.^15^^,^^16^ APC^Δhep^ and βcat^Δex3^ mice develop HCC and HB-like tumours with comparable incidence. These tumours closely recapitulate the histological and transcriptional features of human β-catenin-mutated HCC and mesenchymal HB, respectively.^16^ These models have proven valuable for elucidating β-catenin-driven hepatocyte transformation, and for evaluating diagnostic and therapeutic strategies.[17], [18], [19]

3D *in vitro* culture systems have emerged as valuable approaches to bridge the gap between conventional 2D cultures and *in vivo* models, particularly in cancer research.[20], [21], [22] Tumour-derived organoids (tumouroids) better reproduce native tumour architecture and functions by preserving cell-cell and cell-extracellular matrix interactions, tumour heterogeneity, and microenvironmental complexity. Several methods have been developed, ranging from simple ultra-low attachment (ULA) plate cultures to more sophisticated scaffold-based systems, organ-on-chip platforms and bioreactors, each presenting specific advantages and limitations.^23^ Human liver tumouroid generation predominantly relies on extracellular matrix embedding,[24], [25], [26], [27], [28], [29] whereas ULA plate-based approaches remain less frequently reported.^30^ Moreover, limited data are available for human tumouroids derived from β-catenin-mutated HCC and mesenchymal HB. Previous studies indicate that human HCC-derived tumouroids are more readily established from moderately to poorly differentiated tumours, limiting representation of β-catenin-mutated HCC subtypes.^24^^,^^30^

In the present study, we investigated whether tumouroids generated from our clinically relevant mouse models could address this gap in available tumouroid models for β-catenin-mutated HCC and mesenchymal HB. We used an original dynamic suspension culture system based on a rotating bioreactor (ClinoStar® system, Celvivo). Previous studies have demonstrated that this system supports the generation of HepG2/C3A-derived 3D cultures suitable for liver pathophysiology studies.[31], [32], [33], [34] After optimising culture conditions for mouse hepatocytes with activated β-catenin signalling, we demonstrate that this technology enables the maintenance of murine HCC and HB-like tumouroids while preserving phenotypic and transcriptional features of their tumours of origin. These novel 3D preclinical models provide valuable tools for basic and translational research on β-catenin-driven liver cancers, as demonstrated by their preserved responses to pharmacological treatments.

## Materials and methods

### Mouse models and procedures

For the generation of APC^Δhep^ liver organoids, *Apc*^fl/fl^*;*TTR-CreERT2 mice (2-3-month-old, males) were injected intraperitoneally with 2 mg tamoxifen (MP Biomedicals) diluted in corn oil.^15^ For the generation of APC^Δhep^ liver tumouroids, *Apc*^fl/fl^ mice (2-3-month-old males) were injected with an adenovirus encoding Cre recombinase (AdCre, 0.5 x 10^9^ particles), resulting in APC loss in a fraction of hepatocytes (30-40%), subsequent β-catenin activation, and the development of liver tumours within 4-6 months.^15^ For the generation of βcat^Δex3^ liver tumouroids, C57BL/6J mice were injected with two adeno-associated virus serotype 8 (AAV8) vectors encoding saCas9 and sgRNA targeting *Ctnnb1* to induce the specific deletion of *Ctnnb1* exon 3 in a subset of hepatocytes as previously reported.^16^ In both models, tumour development was followed by 2D ultrasound under isoflurane anesthesia, as previously described.^18^^,^^19^ The maximum cumulative tumour diameter permitted by the ethics committees was 12 mm and was not exceeded. For terminal tissue and blood collection, mice were anesthetised with ketamine (80 mg/kg) and xylazine (5 mg/kg). Mice were housed under a 12/12-h light/dark cycle at 22–24°C with *ad libitum* access to food (Safe A03, SAFE® Complete Care Competence) and water. All animal experiments were conducted in accordance with French government regulations and were approved by two ethics committees (Charles Darwin, Sorbonne Université, APAFIS#16420, APAFIS#45107 and CEEA34, Université de Paris, APAFIS#14472).

### ClinoReactor® preparation

One day before use for organoid or tumouroid culture, the humidification beads inside the ClinoReactors (CelVivo) were hydrated with 20 ml sterile ultra-pure water (CelVivo). The ClinoReactors were then thoroughly washed with DPBS for 15 min at a rotation speed of 15 rpm before cell seeding, as previously described.^32^

### 2D culture of hepatocytes and generation of hepatic organoids from APC^Δhep^ mice

APC^Δhep^ mouse livers were perfused with collagenase six days after tamoxifen administration, and hepatocytes and non-parenchymal cells (NPCs) were isolated and counted as previously described.^35^ Hepatocyte viability was systematically assessed by trypan blue exclusion and was consistently greater than 90%. Detailed procedures for 2D culture are provided in the supplementary methods. For culture in 96-well ULA plates (Greiner), a mixture of 2,000 hepatocytes and 1,000 NPCs was seeded into each well in 100 μl of culture medium. For dynamic culture in the ClinoStar, 500,000 hepatocytes and 250,000 NPCs were seeded into a ClinoReactor rotating at 45 rpm. For culture in Matrigel^TM^ (Corning), a mixture of 50,000 hepatocytes and 25,000 NPCs was suspended in 40 μl of Matrigel and seeded as a dome into each well of a 24-well plate. After polymerisation at 37°C, 500 μl of culture medium was added to each well. After four days, the medium in the 3D cultures was replaced with the same medium without FBS. Thereafter, the medium was renewed every 3-4 days. The cell seeding densities used for ULA and Matrigel cultures were selected based on the conditions most frequently reported in the literature. In contrast, the seeding density for dynamic cultures was optimised experimentally. The maximum number of cells compatible with the ClinoReactors capacity was used to minimise organoid aggregation and fusion during culture. All cultures were maintained at 37°C in a humidified atmosphere containing 5% CO_2_. Bright-field images of organoids were acquired using a MATEO TL digital transmitted-light microscope (Leica Microsystems).

### Generation of hepatic tumouroids from APC^Δhep^ and βcat^Δex3^ mice

Tumours were excised and immediately placed on ice in basal DMEM F-12 medium (Gibco) until processing. The tumours were then gently minced into small pieces using fine scissors, transferred to a 50 ml centrifuge tube containing 10 ml of digestion solution (0.75 mg/ml collagenase (Sigma-Aldrich) and 0.15 U dispase (STEMCELL^TM^ technologies) in basal DMEM F-12 medium), and incubated at 37°C for 20 min under gentle agitation. Three to four rounds of enzymatic digestion were performed, depending on tumour size and type. After digestion, the cell suspension was filtered through a 100-μm cell strainer (Falcon®) to remove aggregates and centrifuged at low speed (50 x g for 5 min at room temperature) to separate hepatocytes (pellet) from NPCs (supernatant). The NPC fraction was then centrifuged at 300 x g for 5 min. The hepatocytes and NPC fractions were then combined, resuspended in 10 ml of HepatiCult™ Organoid Growth Medium (Mouse) (STEMCELL^TM^ technologies), and seeded into a ClinoReactor. The cell suspension was cultured in the ClinoStar system at 45 rpm for up to 16 days, with the medium replaced every 3-4 days. Table S1 summarises the characteristics of the tumour samples used for transcriptomic analysis, including tumour type, tumour size at sacrifice, and the total number of tumouroids generated after tissue fragments had been reserved for RNA extraction and immunohistochemistry (IHC).

### Organoid and tumouroid treatment with inhibitors

After 8 days of culture in the ClinoStar, organoids and tumouroids were transferred into ClinoReactors containing either vehicle (control condition) or WNTinib (1 μM for organoids and 5 μM for tumouroids; MedChemExpress), sorafenib (5 μM, Selleck Chemicals), or trichostatin A (TSA; 0.5 μM; Selleck Chemicals). Treatments were maintained for 5 days and renewed every 2 days.

### Total RNA isolation, reverse transcription and quantitative real-time PCR

Total RNA was extracted from liver tissues, organoids and tumouroids using TRIzol^TM^ reagent (Thermo Fisher Scientific) according to the manufacturer’s instructions. cDNA was synthesised using Maxima reverse transcriptase (Thermo Fisher Scientific). qPCR was performed with specific primers using either BlasTaq^TM^ 2X PCR master mix (abm®) or TaqMan^TM^ gene expression assays (Applied Biosystems). Target gene expression was normalised to that of *B2m,* the most stable reference gene under our experimental conditions*,* and expressed as relative expression using the 2^-ΔΔCt^ method. For selected experiments, a pool of liver tissue cDNA was used as a calibrator.

### Histology, IHC, TUNEL assay and multiplexed immunofluorescence

H&E staining, IHC and TUNEL were performed on formalin-fixed, paraffin-embedded liver tissue sections obtained as previously described.^19^ Detailed procedures for IHC, TUNEL and multiplexed immunofluorescence are provided in the supplementary methods. Whole-slide images of IHC sections were acquired using an AxioScan Z1 scanner (Zeiss), whereas fluorescent and TUNEL images were acquired using an Eclipse E800 microscope (Nikon) and Axio Observer Z1 microscope (Zeiss). The number of stained cells per field was quantified using QuPath software (v0.7.0).^36^ The surface area and nuclear density of organoids and tumouroids were quantified using Fiji software.

### RNA sequencing analysis

RNA sequencing was performed on total RNA extracted from APC^Δhep^ non-tumour liver tissues (n = 8), tumours (n = 5 for HCC; n = 4 for HB), and pools of tumouroids established from each tumour. Of note, two HCCs (tumours 4.B and 4.G) developed in the same mouse liver (Table S1). Detailed RNA sequencing data processing and statistical analysis methods are provided in the supplementary methods. Processed datasets, including lists of up- and down-regulated genes from the different comparisons, are provided in Dataset S1. Heatmaps were generated using the web-based tool Morpheus (https://software.broadinstitute.org/morpheus), and KEGG pathway enrichment analyses were performed using ShinyGO 0.85.1. For gene set enrichment analysis (GSEA), previously published human HCC and HB gene signatures were converted into mouse gene sets using orthology mappings from the MSigDB (Molecular Signatures Database). The resulting mouse ortholog gene sets were used as custom signatures for enrichment analysis (Table S2). Details for GSEA analysis are provided in the supplementary methods.

### Statistical analysis

Statistical analysis was performed using GraphPad Prism (v10.6.1). Differences between two groups were assessed using the Mann-Whitney test. For comparisons involving more than two groups, the Kruskal-Wallis test was performed. A *p* value <0.05 was considered statistically significant.

Detailed information on antibodies, reagents, PCR primer pairs, TaqMan^TM^ gene expression assays, animal models, and experimental resources is provided in the CTAT table.

## Results

### The ClinoStar system enables the generation of liver organoids from APC^Δhep^ mice

For many years, 2D culture of human and murine hepatocytes on collagen matrix has been considered the gold standard approach. However, rapid cell dedifferentiation and cell mortality associated with this culture system have limited its relevance for maintaining hepatocyte identity.^37^ Accordingly, we investigated whether 2D culture altered the phenotype of APC^KO^ hepatocytes isolated from *Apc*^fl/fl^;TTR-CreERT2 mice injected with 2 mg tamoxifen (Fig. S1A). This treatment induces *Apc* deletion in approximately 90% of hepatocytes (Fig. S1B), resulting in the activation of β-catenin-dependent pathways throughout the liver parenchyma. Freshly isolated APC^KO^ hepatocytes overexpressed major direct β-catenin target genes, including *Glul* (encoding glutamine synthetase (GS)), *Axin2*, *Lgr5*, and *Slc13a3* compared with wild-type (WT) hepatocytes isolated from tamoxifen-treated *Apc*^fl/fl^ mice (Fig. S1C). After 24 h of 2D culture, their expression remained significantly elevated in *APC*^KO^ hepatocytes, although the magnitude of induction was substantially lower than in freshly isolated APC^KO^ hepatocytes (Fig. S1C). These findings indicate that 2D monoculture rapidly compromises the maintenance of the β-catenin-activated hepatocyte phenotype.

We next investigated whether dynamic 3D culture could better support the β-catenin-driven phenotype in hepatocytes. We therefore assessed the rotating ClinoStar bioreactor and compared its performance with that of ULA plates and Matrigel-based culture. *APC*^KO^ hepatocytes and NPCs were combined at a 2:1 ratio and cultured either in a ClinoReactor within the ClinoStar system, on ULA plates, or embedded in Matrigel (Fig. 1A). Of note, the initial cell seeding densities varied across culture systems: 750,000 cells per ClinoReactor (75,000 cells/ml), 3,000 cells per ULA well (30,000 cells/ml), and 75,000 cells embedded in a 40 μl Matrigel dome (1.88 x 10^6^ cells/ml). By day 4, APC^ΔHep^ organoids had formed under all culture conditions, demonstrating efficient 3D self-organisation of hepatocytes and NPCs (Fig. 1B,C). By day 8, organoids generated in the ClinoStar system displayed a uniform spherical morphology, and a larger projected area than those formed in ULA plates and Matrigel cultures (145 ± 15, 88 ± 14, 36 ± 14 x 10^3^ μm^2^, respectively). Notably, ClinoStar-derived organoids reached a larger size despite being generated from a lower initial cell density than Matrigel cultures. Organoids cultured in Matrigel also exhibited greater morphological heterogeneity. After 16 days of culture, organoids generated in the ClinoStar system retained a compact and spherical architecture. Similarly, Matrigel-derived organoids remained structurally intact despite their heterogeneous morphology. In contrast, 62% of organoids cultured in ULA plates exhibited structural loosening and reduced size, indicating impaired long-term stability. Together, these findings show that while APC^Δhep^ organoids formed within 4 days in all culture systems tested, the ClinoStar system supported the generation of larger, more homogeneous and structurally stable organoids.

**Fig. 1:**
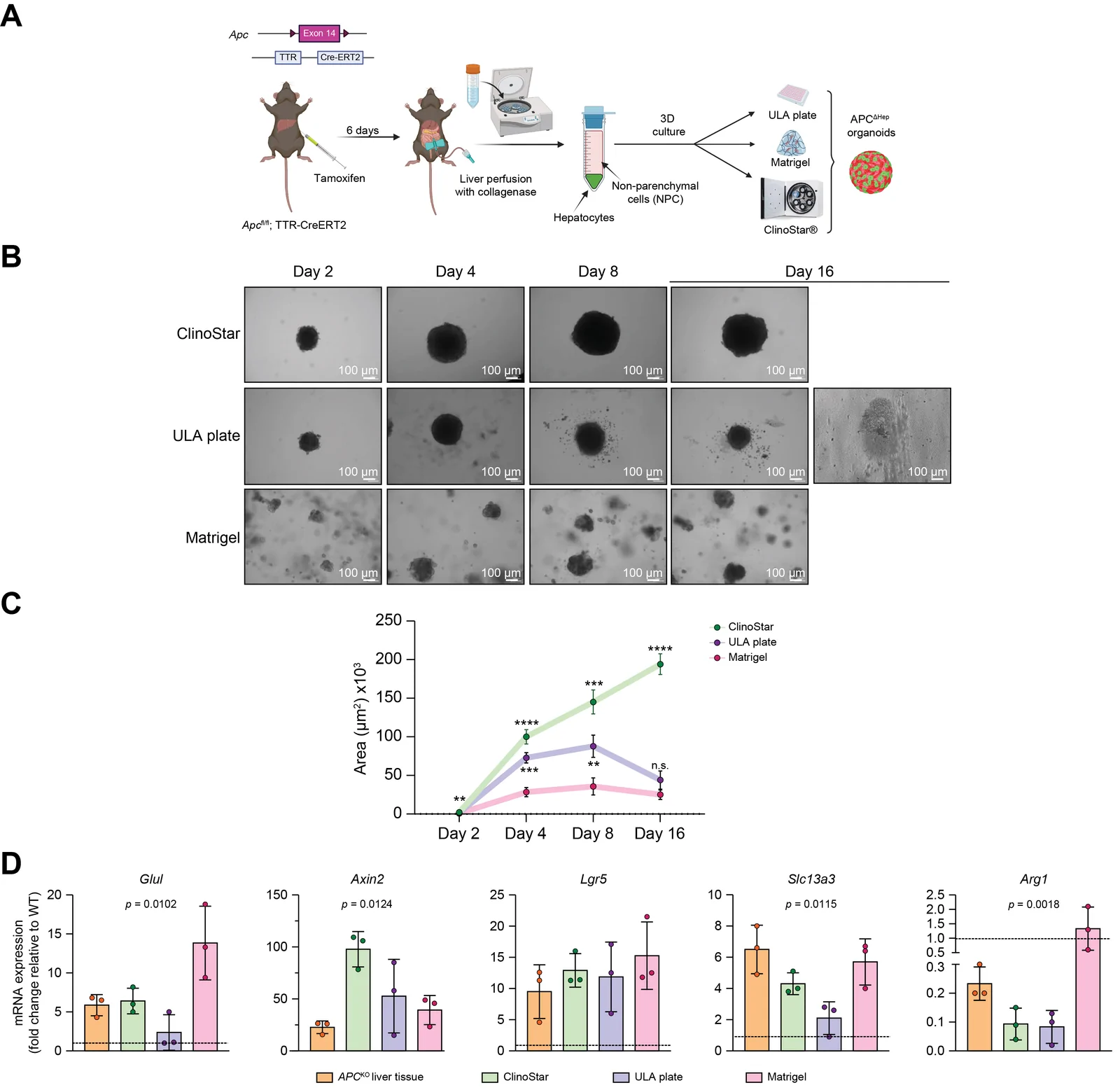
The ClinoStar system supports the growth of APC**^Δhep^** liver organoids**.** (A) Schematic illustration of the experimental design for 3D organoid culture (created with BioRender.com). (B) Representative brightfield images of APC^Δhep^ liver organoids cultured in ULA plates, Matrigel, or the ClinoStar system at days 2, 4, 8, and 16. (C) Quantification of projected organoid area. ∗∗*p <*0.01; ∗∗∗*p <*0.001; ∗∗∗∗*p <*0.0001. Differences were assessed using the Kruskal-Wallis test. (D) Relative transcript expression assessed by RT-qPCR in APC^Δhep^ liver tissue and liver organoids compared with WT liver tissue normalised to 1; the dashed horizontal line indicates this reference level. Global differences were assessed using the Kruskal-Wallis test. Pairwise comparisons were performed using Dunn's multiple comparisons test; no pairwise comparison reached statistical significance. Values are mean ± SD (n = 3 biological replicates, one mouse per replicate). For each replicate, areas were analysed for 12-20 organoids and RNA was extracted from a pool of 20-25 organoids. RT-qPCR, reverse-transcription quantitative PCR; ULA, ultra-low attachment; WT, wild type.

Reverse-transcription quantitative PCR analysis of canonical positive (*Glul*, *Axin2*, *Lgr5*, and *Slc13a3)* and negative (*Arg1*) β-catenin target genes demonstrated that APC^Δhep^ organoids generated using ClinoStar recapitulated the β-catenin-dependent transcriptional signature observed in APC^Δhep^ livers (Fig. 1D). By contrast, transcriptional responses were less consistent in organoids cultured in ULA plates or embedded in Matrigel. ULA-derived organoids showed only limited induction of *Glul* and *Slc13a3*, whereas Matrigel-cultured organoids failed to reproduce the downregulation of *Arg1* after 8 days of culture (Fig. 1D). Histology and IHC analyses were performed to further characterise APC^Δhep^ organoids generated using the ClinoStar system at D8 (Fig. 2A). H&E staining revealed well-preserved tissue architecture in ClinoStar-derived organoids, which also exhibited reduced cell death, as evidenced by decreased cleaved caspase-3 staining (Fig. 2B,C), TUNEL positivity (Fig. 2D), and *Noxa* mRNA expression (Fig. S2A) compared with organoids generated using ULA plates. Ki67 staining revealed low proliferative activity in ClinoStar-derived organoids at D4, comparable to that observed in organoids generated using ULA plates (Fig. 2C). By D8, however, ClinoStar-derived organoids exhibited significantly higher proliferative activity than ULA-derived organoids (Fig. 2B,C), consistent with their improved viability. These findings were corroborated by reverse-transcription quantitative PCR analysis of *Mki6*7 mRNA expression (Fig. S2B). Furthermore, APC^Δhep^ organoids generated using the ClinoStar system showed a trend toward higher proliferative activity than those cultured in Matrigel (Fig. S2B).

**Fig. 2:**
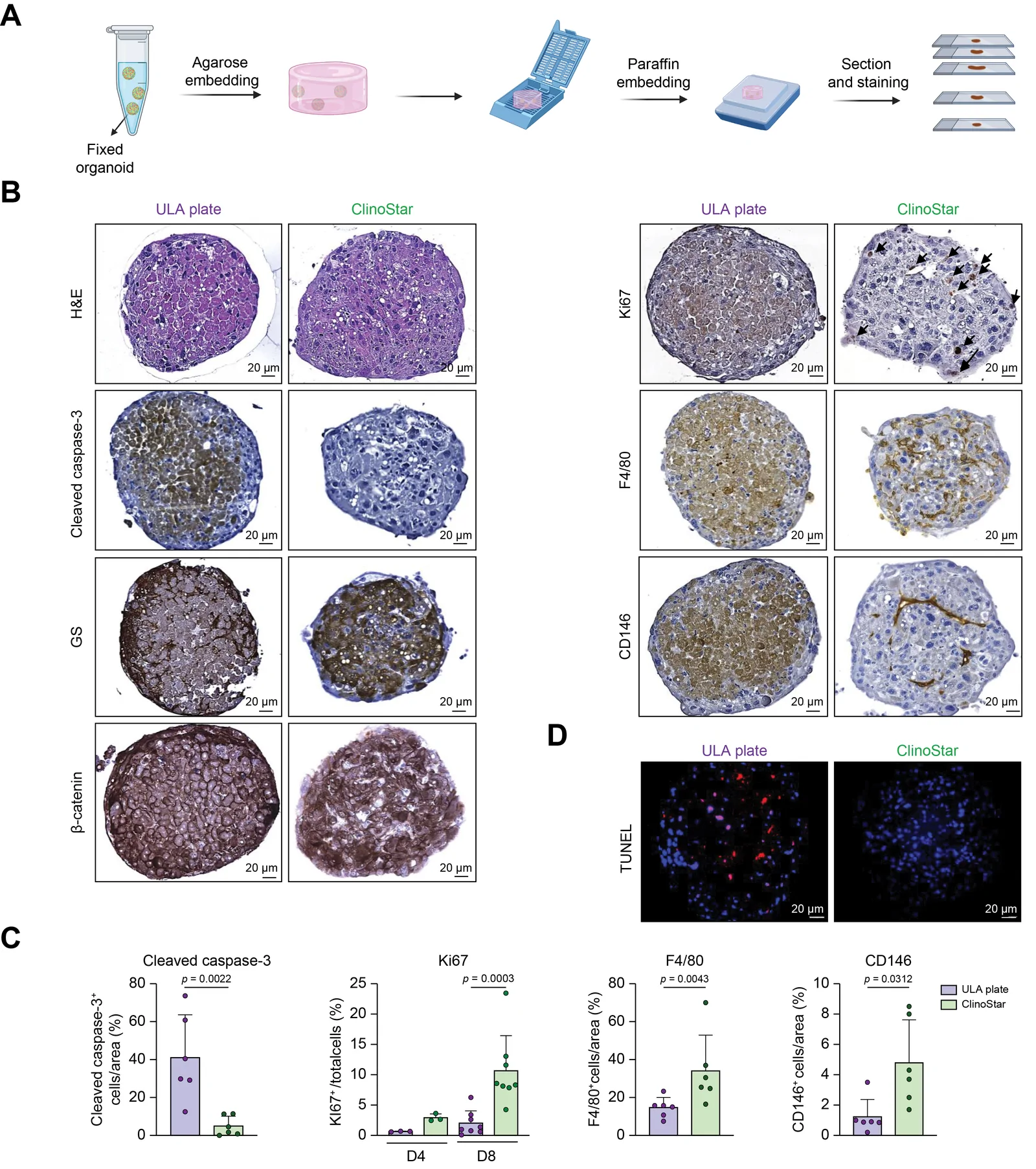
The ClinoStar system promotes an organised tissue architecture in APC^Δhep^ liver organoids. (A) Schematic illustration of the spheroid inclusion protocol (created with BioRender.com). (B) Representative H&E staining and immunostaining in APC^Δhep^ organoids obtained after 8 days in ULA plates or in the ClinoStar. Black arrows indicate Ki67-positive nuclei. (C) Quantification of immunostaining. (D) Representative images of apoptotic cells detected by TUNEL staining (red), with nuclei counterstained with DAPI (blue), in organoids cultured for 8 days in ULA plates or ClinoStar. Values are mean ± SD (n = 6 biological replicates, one mouse per replicate; 8-14 organoids per replicate). Differences were assessed using the Mann-Whitney test. ULA, ultra-low attachment.

ClinoStar-derived organoids showed homogeneous GS staining together with membranous, cytoplasmic and nuclear localisation of β-catenin, consistent with sustained activation of the β-catenin pathway. Importantly, NPCs were also detected, including F4/80-positive macrophages and CD146-positive endothelial cells (Fig. 2B, C). In contrast, ULA-derived organoids exhibited weaker immunostaining for GS, β-catenin, F4/80, and CD146, accompanied by increased background staining (Fig. 2B, C). To further assess hypoxic stress, hypoxia was evaluated by carbonic anhydrase 9 (CA9) immunostaining and by measuring the expression of the HIF1-responsive genes *Epo* and *Vegfa*. ClinoStar-derived organoids exhibited reduced CA9 staining and lower *Epo* and *Vegfa* expression than ULA-derived organoids (Fig. S2C and D). Collectively, these data demonstrate that the ClinoStar system offers favourable conditions for the generation of β-catenin-activated liver organoids by preserving tissue architecture, cellular viability, and cellular heterogeneity while limiting hypoxic stress.

### The ClinoStar system enables the generation of tumouroids from APC^Δhep^ and βcat^Δex3^ mouse liver tumours

These findings prompted us to investigate whether the ClinoStar system could also be used to generate β-catenin-activated tumouroids. To this end, *Apc*^fl/fl^ mice were injected with a low dose of an adenovirus encoding Cre recombinase (AdCre) and monitored by ultrasound imaging for tumour development over several months. Once tumours were detected, they were harvested, mechanically and enzymatically dissociated, and seeded into bioreactors (Fig. 3A). Two types of tumours were obtained from these mice: well-differentiated APC^Δhep^ HCC and undifferentiated APC^Δhep^ HB-like tumours.

**Fig. 3:**
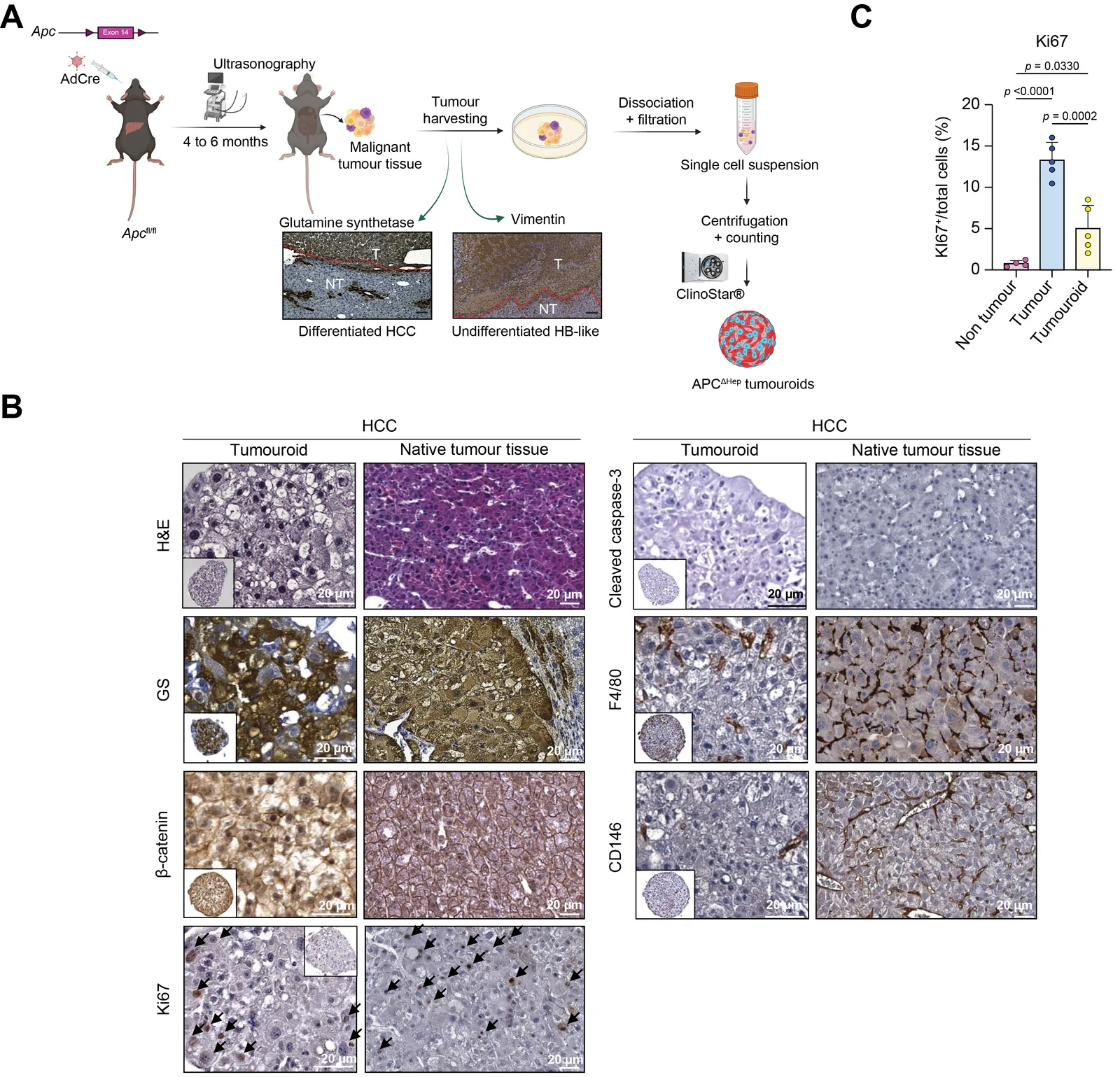
Culture in the ClinoStar generates and preserves APC^Δhep^ HCC tumouroids. (A) Schematic illustration of the experimental design for 3D tumouroid culture in the ClinoStar system (created with BioRender.com). (B) Representative H&E staining and immunostaining in tumouroids generated after 8 days of culture in the ClinoStar, compared with corresponding native HCC tissue. Black arrows indicate Ki-67 positive nuclei. (C) Quantification of Ki67-positive cells (n = 4-5 biological replicates, one mouse per replicate). Differences were assessed using the Kruskal-Wallis test. For each biological replicate, 7-13 tumouroids were analysed. HCC, hepatocellular carcinoma.

After 8 days of culture in the ClinoStar, tumour cells and NPCs isolated from both HCC and HB-like tumours formed 3D structures, yielding approximately 30-75 tumouroids per culture with diameters ranging from 150 to 200 μm. These tumouroids reproduced key structural and cellular features of their corresponding tumour tissue. APC^Δhep^ HCC and HB-like tumouroids retained activation of the β-catenin pathway, as demonstrated by cytoplasmic GS staining and nuclear β-catenin staining (Fig. 3, Fig. 4; Table 1). HB-like tumouroids also exhibited vimentin expression, consistent with their mesenchymal phenotype (Fig. 4A,B). After 16 days of culture, HCC-derived tumouroids showed no detectable CA9 immunostaining within their core, whereas CA9-positive areas were observed in some HB-like tumouroids (Fig. S3A). This observation is consistent with the gene expression data showing higher *Vegfa* expression in both primary HB-like mouse tumours and the corresponding tumouroids than in their HCC counterparts (Fig. S3B), suggesting that the limited hypoxic response detected in HB-like tumouroids may primarily reflect the intrinsic characteristics of the tumour cells rather than the culture conditions. Overall, both tumouroid models exhibited little evidence of marked hypoxic stress under the conditions used. Consistent with this finding, cleaved caspase-3 staining was absent from the core of tumouroids, regardless of their tumour of origin, whereas Ki67-positive hepatocytes were distributed throughout the structures (Fig. 3, Fig. 4A). As previously observed in the preneoplastic organoids (Fig. 2B,C), F4/80 and CD146 staining confirmed the presence of macrophages and endothelial cells in tumouroids, respectively (Fig. 3, Fig. 4A). In addition to conventional chromogenic IHC, HCC tumouroids were analysed using a high-throughput automated multiplex immunofluorescence platform based on high-pressure microfluidic circuits. As shown in Fig. S4, markers specific for the different cellular populations were readily detected within APC^Δhep^ HCC tumouroids.

**Fig. 4:**
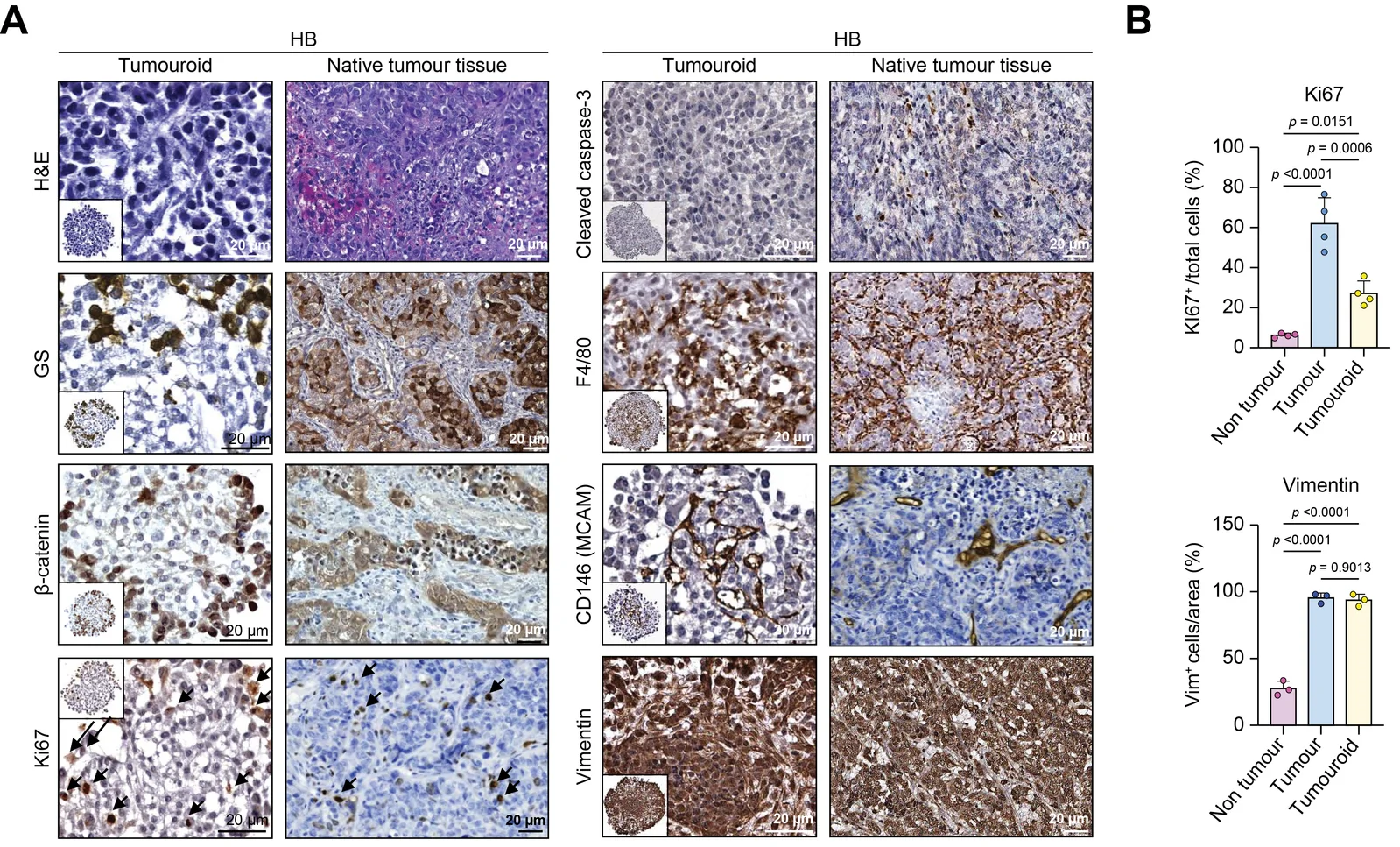
Culture in the ClinoStar generates and preserves APC^Δhep^ HB-like tumouroids. (A) Representative H&E staining and immunostaining in tumouroids generated after 8 days of culture in the ClinoStar, compared with corresponding native HB-like tissue. Black arrows indicate Ki67-positive nuclei. (B) Quantification of Ki67- and vimentin-positive cells (n = 3-4 biological replicates, one mouse per replicate). For each biological replicate, 6-10 tumouroids were analysed. Differences were assessed using the Kruskal-Wallis test. HB, hepatoblastoma.

**Table 1:** Immunohistochemical characterisation of murine HCC and HB and their corresponding tumouroids.

Name	H-score GS	Membranous β-catenin (%)	Nuclear β-catenin (%)
**HCC**			
Tumour #2	300	100	60
Tumouroid #2	275	100	93
Tumour #4B	300	97	58
Tumouroid #4B	283	100	100
Tumour #4G	300	100	90
Tumouroid #4G	217	98	67
Tumour #7	300	100	80
Tumouroid #7	272	80	55

**HB-like**			
Tumour #5	200	55	50
Tumouroid #5	75	85	33
Tumour #9	165	75	85
Tumouroid #9	218	83	70
Tumour #14	170	95	30
Tumouroid #14	95	90	29

H-score (range 0-300) of GS and percentage of membranous and nuclear β-catenin-positive cells in murine HCC, HB, and their corresponding tumouroids. Because GS expression is substantially lower in HB than in HCC, immunostaining conditions were adjusted to optimise signal detection in each tumour type. Therefore, H-score values are not intended for direct quantitative comparison between HCC and HB. Data are presented for each individual tumour and its corresponding tumouroids (3-7 tumouroids for each tumour). GS, glutamine synthetase; HB, hepatoblastoma; HCC, hepatocellular carcinoma.

We further validated these data using βcat^Δex3^ HCC, which shares a similar transcriptomic profile with APC^Δhep^ HCC.^16^ Tumouroids derived from βcat^Δex3^ HCC retained the histological architecture of their tumour of origin (Fig. S5). Altogether, these data indicate that dynamic culture in the ClinoStar system enables the generation of tumouroids that maintain key histological and cellular features of murine β-catenin-activated HCC and HB-like tumours while preserving intra-tumour cellular heterogeneity.

### APC^Δhep^ tumouroids cultured in the ClinoStar system retain the transcriptomic profile of their tumour of origin

To further characterise APC^Δhep^ HCC (n = 5) and HB-like tumouroids (n = 4), RNA sequencing was performed, and their transcriptomic profiles were compared with those of the corresponding native tumours and adjacent non-tumour tissues. Principal component analysis showed that each tumouroid clustered closely with its tumour of origin, whereas non-tumour tissues formed a distinct cluster (Fig. 5A for HCC and Fig. 6A for HB). One sample pair (HCC#1/tumouroid#1) appeared as an outlier. Notably, HCC#1 was among the largest of the tumours analysed, and exhibited haemorrhagic and necrotic areas (Table S1). Differential gene expression analysis using MA plots further demonstrated the high degree of similarity between tumouroids and their corresponding tumours (Fig. 5B for HCC and Fig. 6B for HB). In HCC-derived tumouroids, only two genes (*Clec4d* and *Gm10273*) were differentially expressed compared with the corresponding primary tumours (Fig. 5B). Similarly, HB-like tumouroids showed a highly conserved transcriptomic profile, with only *Fam177a2* and *2810047C21Rik* genes significantly upregulated compared with the tumour of origin (Fig. 6B). We next examined the expression of gene signatures characteristic of each tumouroid type relative to the corresponding non-tumour liver tissue (Fig. 5, Fig. 6C, S6, and S7; Tables S3 and S4; samples HCC#1/tumouroid#1 were excluded from this analysis). Consistent with previous reports in APC^Δhep^ HCC,^16^^,^^18^^,^^19^^,^^38^^,^^39^ HCC-derived tumouroids exhibited high expression of *Ctnnb1* target genes, including both canonical targets and adult liver-specific targets involved in glutamine, bile acid, and xenobiotic metabolism. They also displayed increased expression of genes associated with inflammation and redox signalling. Conversely, genes involved in amino acid catabolism, known to be repressed by β-catenin in mature hepatocytes, were expressed at low levels (Fig. 5C and S6; Table S3). Similarly, HB-like tumouroids recapitulated APC^Δhep^ HB-like tumours,^16^ including high expression of canonical *Ctnnb1* target genes, *Apc*^Δhep^ embryonic liver genes, mesenchymal markers and YAP pathway-associated genes (Fig. 6C and S7; Table S4). Transcripts encoding vascular markers, including *Mcam*/Cd146 were significantly upregulated in both APC^Δhep^ HCC and HB-like tumouroids, consistent with the IHC detection of endothelial cells. KEGG pathway enrichment analysis was subsequently performed by comparing tumouroids with their corresponding non-tumour tissues (Fig. S8A–D). In HCC-derived tumouroids, the most enriched pathways were associated with cell cycle progression, xenobiotic metabolism and glutathione metabolism (Fig. S8A), whereas pathways related to amino acid metabolism were among the most downregulated (Fig. S8B). In HB-like tumouroids, pathways involved in mitosis and cell division showed the highest enrichment scores (Fig. S8C), while several lipid metabolism pathways were significantly downregulated (Fig. S8D). These pathway-level alterations closely mirrored the biological characteristics of the corresponding native tumours, namely a predominantly metabolic phenotype in HCC and a highly proliferative phenotype in HB-like tumours. Collectively, these findings demonstrate that the ClinoStar-based culture system generates murine HCC and HB-like tumouroids that preserve the transcriptomic programmes, cellular complexity, and biological features of the tumours of origin.

**Fig. 5:**
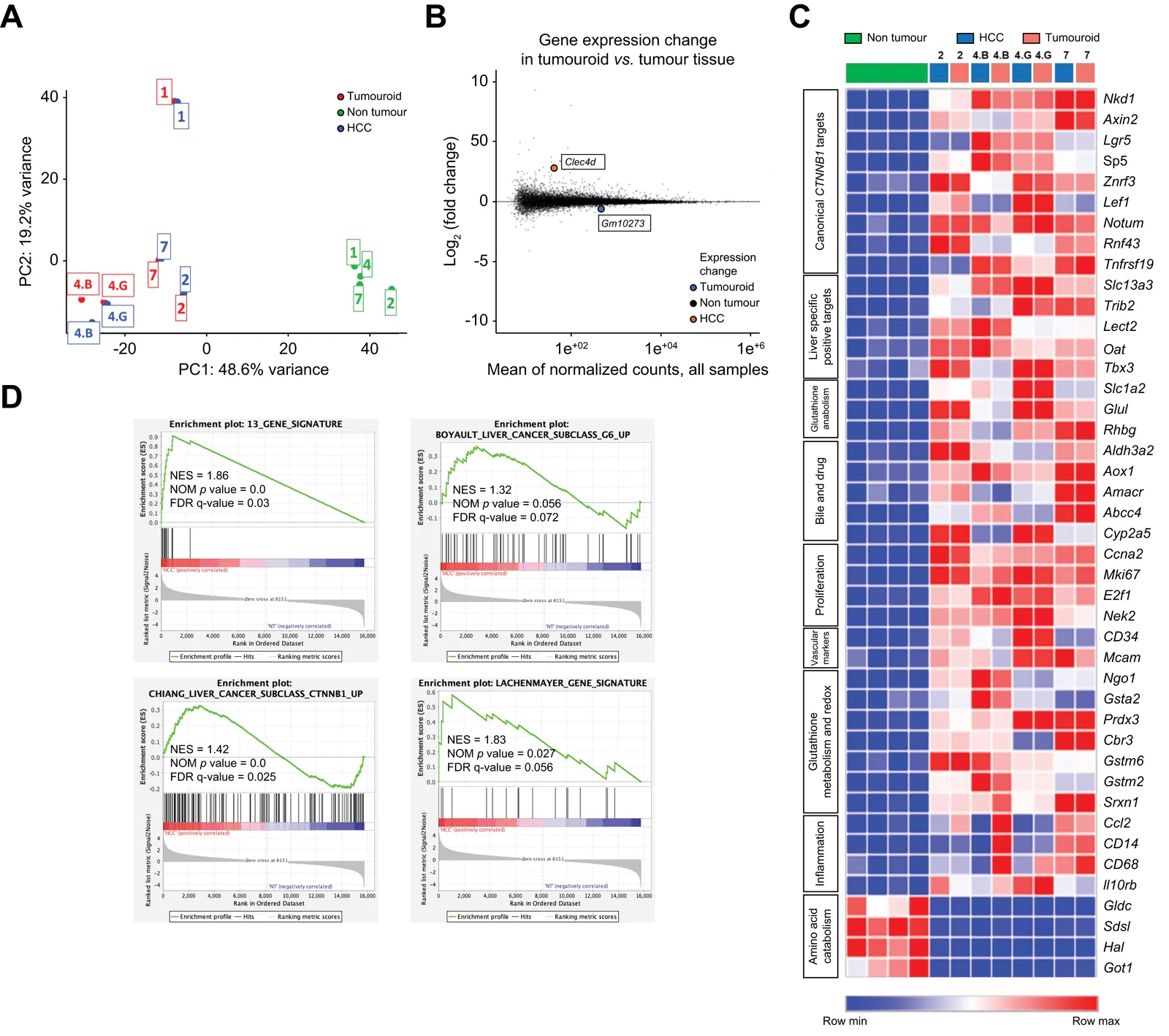
Culture in the ClinoStar maintains the transcriptomic programmes of APC^Δhep^ HCC tumouroids. (A) PCA of normalised gene expression profiles from five pooled tumouroid samples (each generated from a single HCC), five HCC, and four adjacent non-tumour liver tissues. (B) MA plot showing gene expression differences between each pool of tumouroids and its corresponding HCC. Each dot represents a gene, plotted as log_2_ fold change *vs*. mean normalised counts across all samples. (C) Heatmap of differentially expressed genes between four pools of tumouroids, four HCC, and four adjacent non-tumour liver tissues. Samples were clustered using unsupervised hierarchical clustering. (D) GSEA highlighting significantly enriched signatures between murine tumouroids and human HCC tissues. Enrichment results are shown for selected gene sets, with NES, NOM *p* values, and FDR q-values indicated. FDR, false discovery rate; GSEA, gene set enrichment analysis; HCC, hepatocellular carcinoma; NES, normalised enrichment score; NOM, nominal; PCA, principal component analysis.

**Fig. 6:**
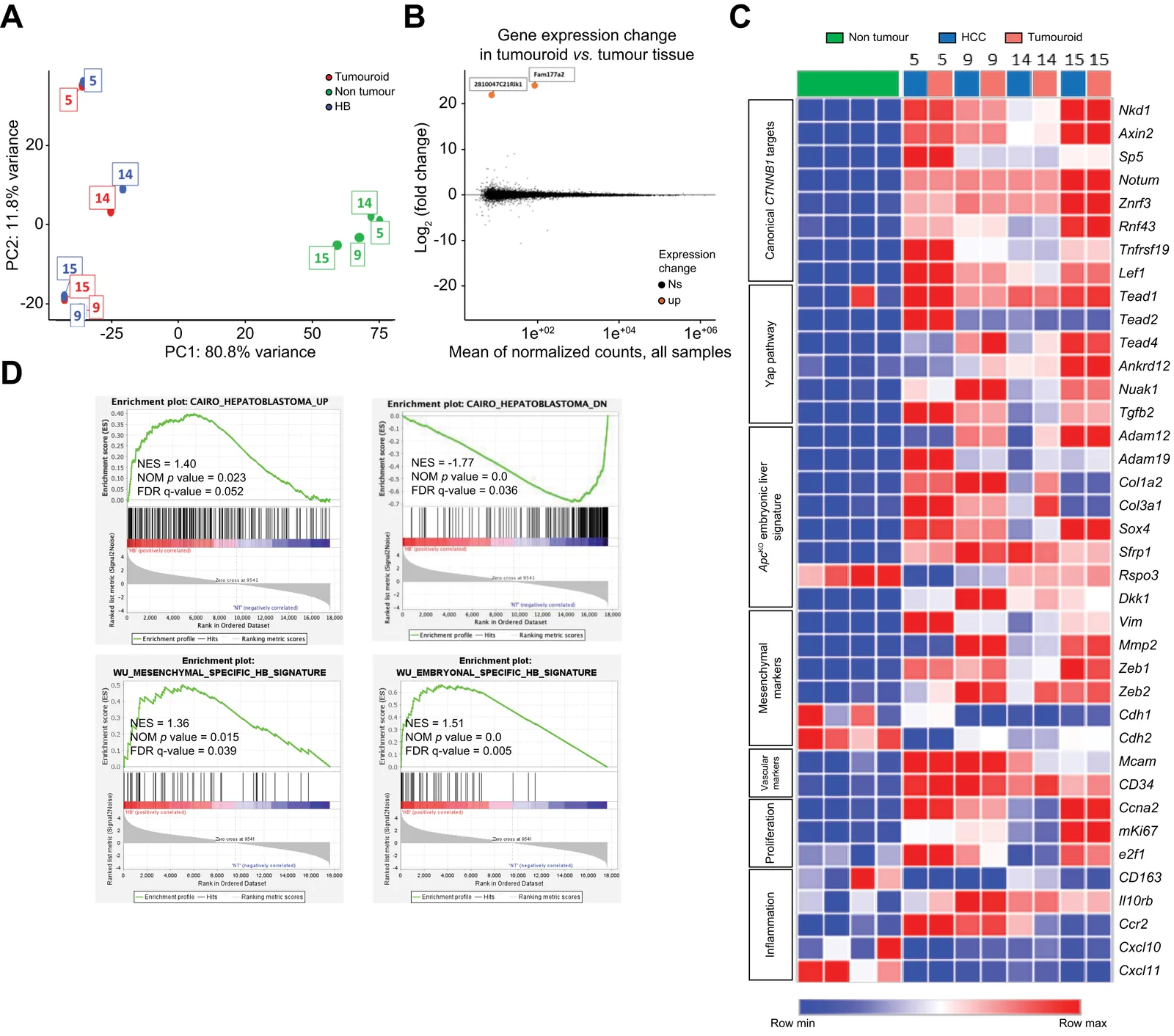
Culture in the ClinoStar maintains the transcriptomic programmes of APC^Δhep^ HB-like tumouroids. (A) PCA of normalised gene expression profiles from four pooled tumouroid samples (each generated from a single HB), four HB, and four adjacent non-tumour liver tissues. (B) MA plot showing gene expression differences between each pool of tumouroids and its corresponding HB-like tumour. Each dot represents a gene, plotted as log_2_ fold change *vs*. mean normalised counts across all samples. (C) Heatmap of differentially expressed genes between four pools of tumouroids, four HB, and four adjacent non-tumour liver tissues. Samples were clustered using unsupervised hierarchical clustering. (D) GSEA highlighting significantly enriched signatures between murine tumouroids and human HB tissues. Enrichment results are shown for selected gene sets, with NES, NOM *p* values, and FDR q-values indicated. FDR, false discovery rate; GSEA, gene set enrichment analysis; HCC, hepatocellular carcinoma; NES, normalised enrichment score; NOM, nominal; PCA, principal component analysis.

### Murine tumouroids retain features of human HCC and HB transcriptional programmes

To evaluate the relevance of the murine tumouroids generated from HCC, we investigated whether they recapitulated transcriptional programmes associated with β-catenin-mutated human HCC. GSEA was performed using four previously published human β-catenin-related HCC signatures, including the Boyault G6 Up signature,^40^ the Chiang *CTNNB1* Up signature,^41^ the Lachenmayer signature,^42^ and the recently developed mutated β-catenin gene signature, a robust 13-gene transcriptional classifier.^43^ All four signatures were enriched in murine HCC-derived tumouroids (Fig. 5D). We next examined whether tumouroids derived from murine HB recapitulate molecular features observed in human HB. To this end, GSEA was performed using six independent human HB gene signatures (Figs 6D and S8E). The enrichment patterns observed across these datasets revealed a strong concordance between murine HB-like tumouroids and human HB transcriptional programmes. Notably, murine HB-like tumouroids were significantly enriched for the hypomethylated gene signature described by Carrillo-Reixach *et al.*,^44^ while exhibiting negative enrichment of the corresponding downregulated signature. These findings indicate that they share molecular characteristics with the Epi-CB subclass of human HB, which is characterised by global DNA hypomethylation. In addition, these tumouroids displayed significant enrichment for both the embryonal and mesenchymal signatures defined by Wu *et al.*,^28^ suggesting the coexistence of developmental and mesenchymal transcriptional programmes associated with more immature HB phenotypes. Together, these results demonstrate that murine HCC and HB-like tumouroids preserve key transcriptional features associated with human β-catenin-driven liver cancers, supporting their relevance as models to investigate biologically relevant aspects of these diseases.

### β-catenin-driven organoids and tumouroids exhibit drug-induced antiproliferative and pro-apoptotic responses

To assess whether ClinoStar-generated organoids and tumouroids represent relevant preclinical models for pharmacological testing, we evaluated their response to three compounds with distinct mechanisms of action: WNTinib, a multi-kinase inhibitor previously shown to target *CTNNB1*-mutated liver cancer cells,^45^^,^^46^ sorafenib, a clinically approved multikinase inhibitor targeting RAF kinases and angiogenic signalling pathways,^47^ and trichostatin A (TSA), a histone deacetylase inhibitor reported to exert potent antiproliferative and pro-apoptotic effects in β-catenin-activated hepatic cancer cell lines.^48^ APC^Δhep^ organoids were cultured for 8 days before compound treatment was initiated and maintained for an additional 5 days. WNTinib induced marked morphological alterations, including disruption of organoid architecture and the appearance of cell-depleted areas within the organoid core (Fig. 7A). These structural changes were associated with reduced proliferative activity, as assessed by Ki67 immunostaining (Fig. 7A, B), and activation of an apoptotic programme, evidenced by increased cleaved caspase-3 immunolabelling (Fig. 7A,B), loss of nuclear density (Fig. 7C), increased TUNEL positivity (Fig. S10A), increased *Noxa* expression and decreased *Bcl2l1* expression (Fig. 7D). TSA elicited an even stronger pro-apoptotic response (Fig. S9A–C), whereas sorafenib produced comparatively weaker effects (Fig. S9D and E). Notably, antiproliferative and pro-apoptotic effects were not accompanied by a detectable reduction in organoid size during the treatment period (Figs 7E and S9F). βcat^Δex3^ HCC-derived tumouroids also responded to WNTinib treatment by undergoing cell death and reduced proliferative activity (Figs 7F-I and S10B). In an exploratory experiment using βcat^Δex3^ tumouroids derived from a single independent tumour, TSA treatment was associated with a more pronounced apoptotic response, with apoptotic cells representing up to 80% of the tumouroid area (Fig. S9G–I). Again, no consistent change in tumouroid size was observed under any of the treatment conditions (Figs 7J and S9J). Together, these results demonstrate that organoids and tumouroids generated in the ClinoStar are amenable to pharmacological manipulation and can discriminate between compounds inducing distinct levels of biological response, supporting their potential for drug screening applications.

**Fig. 7:**
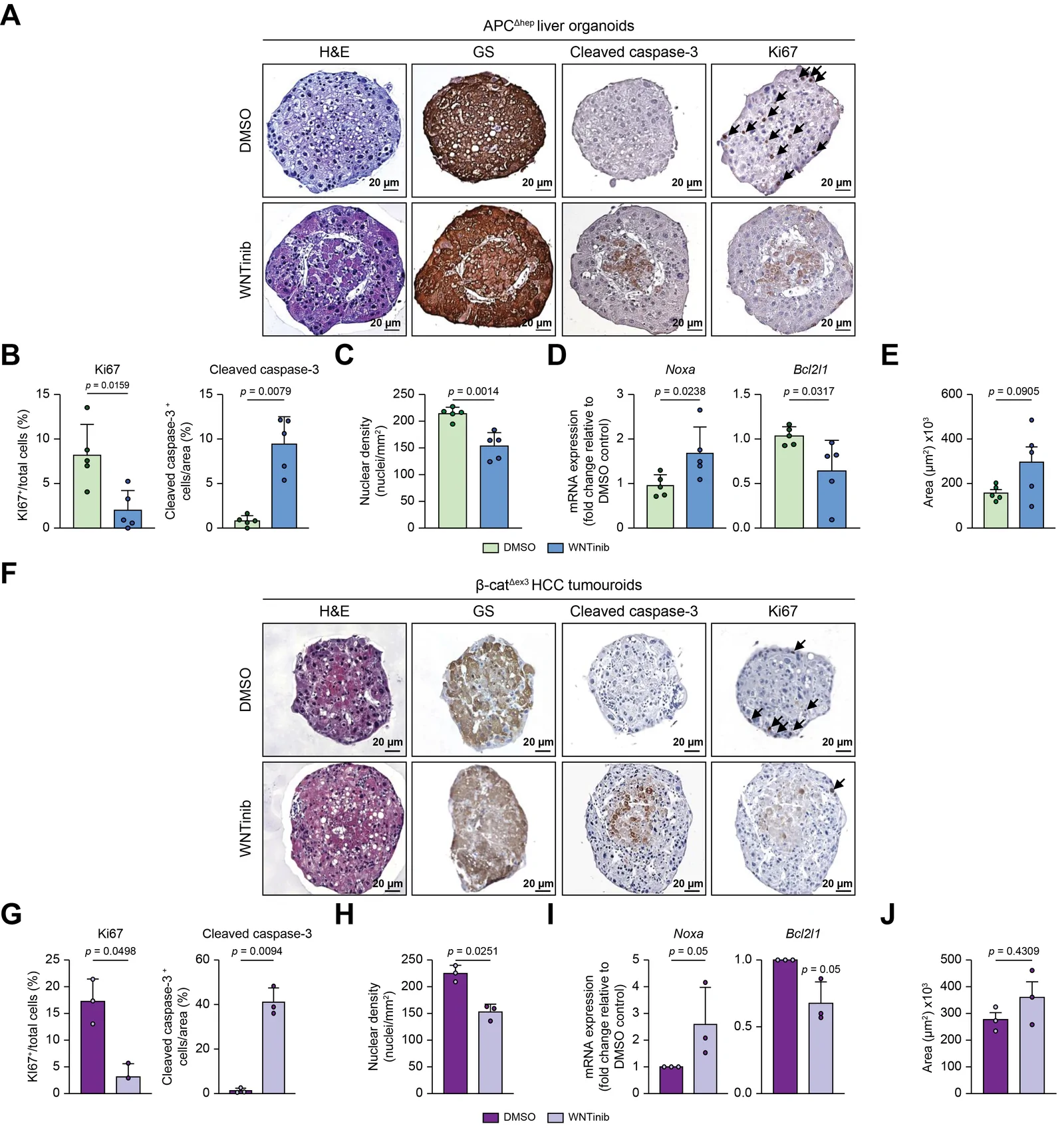
WNTinib induces apoptosis and impairs proliferation in APC^Δhep^ liver organoids and βcat^Δex3^ HCC tumouroids. (A) Representative H&E staining and immunostaining in APC^ΔHep^ liver organoids after 8 days of culture in the ClinoStar and treated with WNTinib (1 μM) or DMSO for 5 additional days. Black arrows indicate Ki67-positive nuclei. (B) Quantification of immunostaining. (C) Quantification of nuclear density. (D) Relative transcript expression assessed by RT-qPCR. (E) Quantification of projected organoid area. n = 5 biological replicates, one mouse per replicate. For each biological replicate, RNA was extracted from a pool of 20-25 organoids and 8-15 tumouroids were analysed for protein expression. (F) Representative H&E staining and immunostaining in βcat^Δex3^ HCC tumouroids after 8 days of culture in the ClinoStar system and treated with WNTinib (5 μM) or DMSO for 5 additional days. Black arrows indicate Ki67-positive nuclei. (G) Quantification of immunostaining. (H) Quantification of nuclear density. (I) Relative transcript expression assessed by RT-qPCR. (J) Quantification of projected organoid area. n = 3 biological replicates, one mouse per replicate. For each biological replicate, RNA was extracted from a pool of 20-25 organoids and 5-10 tumouroids were analysed for protein expression. Values are mean ± SD. Differences were assessed using the Mann-Whitney test. HCC, hepatocellular carcinoma; RT-qPCR, reverse-transcription quantitative PCR.

## Discussion

In this study, we established a matrix-free dynamic culture platform enabling the generation of murine preneoplastic organoids from livers with hepatocyte-specific β-catenin activation, together with tumouroids derived from β-catenin-activated well-differentiated HCC and poorly differentiated HB-like tumours. Histological, immunohistochemical and transcriptomic analyses demonstrated that these 3D models faithfully preserve the architecture, cellular composition and molecular features of their tumours of origin. Our findings demonstrate that dynamic suspension culture can maintain the complexity of β-catenin-driven liver tumours while enabling downstream molecular and pharmacological analyses.

The successful establishment of these models relies on the unique culture properties of the ClinoStar rotary bioreactor. By continuously counterbalancing gravitational forces, the system maintains cells in suspension under near-microgravity conditions without extracellular matrix components. This low-shear environment promotes nutrient and oxygen distribution while limiting hypoxia and cell death, contributing to the morphological homogeneity and reproducible size of liver organoids and tumouroids, consistent with recent studies using the ClinoStar system to generate brain organoids^49^ and lung tumouroids.^50^ Beyond these biological advantages, the matrix-free configuration simplifies tissue handling, avoids Matrigel-associated variability, facilitates recovery of intact tumouroids for molecular analyses, and enables the production of sufficient material for extensive histological characterisation. Together, these features provide a standardised and experimentally tractable alternative to conventional matrix-based culture systems.

The ClinoStar system preserves the molecular identity of individual tumours and captures inter-tumour heterogeneity rather than driving samples towards a common transcriptional profile. Principal component analysis demonstrated that tumouroids clustered with their corresponding tumour of origin, including HCC#1, a large tumour characterised by extensive haemorrhagic and necrotic regions and a distinctive transcriptional programme. These findings demonstrate preservation of tumour-specific features, an essential prerequisite for applications such as drug screening, where inter-patient variability influences therapeutic response.

A further strength of this culture system is its preservation of tumour cellular heterogeneity. Throughout at least 8 days of culture, differentiated and poorly differentiated tumour hepatocytes remained associated with macrophages and endothelial cells, indicating maintenance of key components of the tumour microenvironment *ex vivo*. This multicellular organisation provides a physiologically relevant platform to investigate tumour cell plasticity and tumour-microenvironment interactions. Although circulating immune cell populations are not represented in the current model, limiting studies of immune-mediated therapeutic responses, future incorporation of additional immune components should broaden its utility for immuno-oncology research.

Preclinical modelling of β-catenin-driven primary liver cancers remains challenging. Conventional liver cancer cell lines are readily amenable to drug screening but fail to reproduce the cellular heterogeneity and tissue architecture of primary tumours. Patient-derived xenografts retain these characteristics but are constrained by variable engraftment efficiency, high costs, lengthy experimental timelines and limited compatibility with medium-throughput therapeutic testing. Patient-derived tumouroids have emerged as an attractive complementary approach, particularly following the pioneering work of Huch and colleagues demonstrating long-term expansion of primary HCC and cholangiocarcinoma organoids.^24^ However, subsequent studies consistently reported greater success in establishing tumouroids from poorly differentiated HCC than from well-differentiated *CTNNB1*-mutated tumours, leaving an important unmet need in available experimental models.^25^^,^^29^^,^^30^ Likewise, although several groups have established human HB organoids,[26], [27], [28] transcriptomic information remains limited for tumours displaying mesenchymal features.

Our models directly address these limitations. Transcriptomic analyses demonstrated that murine HCC and HB-like tumouroids were enriched for molecular signatures previously associated with human *CTNNB1*-mutated HCC and mesenchymal HB, respectively. These findings indicate that the biological programmes in these murine tumours parallel those observed in their human counterparts, supporting the translational relevance of the models. The ability to reproducibly generate tumouroids representative of clinically relevant tumour subtypes should facilitate mechanistic studies while expanding the repertoire of preclinical models available for β-catenin-driven liver cancers.

A major objective of developing these organoids and tumouroids was to establish a biologically relevant platform for preclinical drug evaluation. Consistent with this goal, both preneoplastic organoids and HCC tumouroids responded to WNTinib, a recently described transcriptional inhibitor of mutant β-catenin, with reduced proliferation and increased apoptosis, in agreement with previous findings obtained in matrix-embedded organoid models.^45^^,^^46^ In contrast, sorafenib showed limited activity under our experimental conditions, whereas TSA induced a marked pro-apoptotic response. These results demonstrate that the ClinoStar system can discriminate between compounds with distinct therapeutic effects while preserving clinically relevant tumour features.

Although the number of tumouroids obtained from an individual mouse tumour is inherently finite, the ClinoStar platform routinely produces sufficient tumouroids (typically 30-75 per tumour) that retain the molecular identity of the tumour of origin. Individual tumouroids can be distributed into multiwell plates using a dedicated dispensing system and exposed to different treatment conditions, enabling medium-throughput drug testing and functional prioritisation of candidate therapies. By maximising experimental information obtained from each tumour, this approach also contributes to the principle of reduction in animal research.

In conclusion, we describe a robust matrix-free methodology for generating murine tumouroids from β-catenin-driven HCC and HB. These models recapitulate tumour architecture, cellular heterogeneity and clinically relevant molecular features while providing a practical platform for histological, molecular and pharmacological investigations. By bridging genetically engineered mouse models and functional preclinical studies, this approach provides a valuable resource for investigating liver tumourigenesis and accelerating therapeutic development. Extending this strategy to additional murine liver cancer models and, ultimately, to human liver tumours could further broaden its translational applications.

## Abbreviations

AAV8, adeno-associated virus serotype 8; AdCre, adenovirus encoding Cre recombinase; CA9, carbonic anhydrase; GS, glutamine synthetase; HB, hepatoblastoma; IHC, immunohistochemistry; NPC, non-parenchymal cells; PDX, patient-derived xenograft; TSA, trichostatin A; ULA, ultra-low attachment; WT, wild-type.

## Authors’ contributions

C.D.-M. and V.B.M. conceived the study and supervised all aspects of the project. V.B.M., F.L. and A.G. performed hepatocyte isolation and cell culture experiments. V.B.M. and A.H. performed histology, immunodetection and RT-qPCR. I.L. performed liver ultrasonography. SB followed tumour progression by ultrasonography. A.A.-M. and J.A. analyzed IHC staining. A.G. managed *Apc*^fl/fl^ mouse breeding. C.D.-M. and S.C. obtained funding supports. C.D.-M. and V.B.M. wrote the manuscript with contributions from all authors. All authors read and approved the final manuscript.

## Data availability

The sequencing data generated in this study have been deposited in ENA under accession number PRJEB115263. Other data supporting the findings of this study are available from the corresponding author upon reasonable request.

## Financial support

This study has been supported by grants from Institut National de la Santé et de la Recherche Médicale (INSERM), Association Française pour l’Etude du Foie (AFEF, to CDM), Institut National du Cancer (to CDM and VBM, PLBIO 24-107, and to SC and VBM, PLBIO 21-268, INCa_1602), and Ligue Nationale Contre le Cancer (to SC and VBM, AAPEAC2022.LCC/VM). VBM salary is funded by Ligue Nationale Contre le Cancer (AAPEAC2022.LCC/VM, INCa_1602 PLBIO 21-268, and by INCa PLBIO 24-107).

## Conflict of interest

The authors declare no conflicts of interest pertaining to this manuscript.

Please refer to the accompanying ICMJE disclosure forms for further details.
