# Supplementary material for: Novel 3D models for β-catenin-driven liver cancers
Source: JHEP Rep. 2026 Sep 18;8(12):102040. doi: 10.1016/j.jhepr.2026.102040 (PMC13755961; doi:10.1016/j.jhepr.2026.102040)
Supplement: Supplementary file 1 [file mmc1.pdf]

Novel 3D models for β-catenin-driven liver cancers

Vanessa Bou Malham, Fanny Léandre, Akila Hamimi, Isabelle Lagoutte, Sandrine Bouchet, Alexandre Archambault-Marsan, Jérémy Augustin, Angélique Gougelet, Sabine Colnot, Christèle Desbois-Mouthon

Table of contents

Supplementary materials and methods………………...2

Supplementary figures…….……………………………………………………….....…5

Supplementary tables…….……………………………………………………………..22

Supplementary materials and methods

2D culture of hepatocytes from APC$^{\Delta hep}$ mice. 500,000 hepatocytes were seeded onto 6-well plates coated with rat tail collagen type I (Gibco) in William's E medium with GlutaMAX™ (Gibco) supplemented with 10% fetal bovine serum (FBS), 100 U/mL penicillin, 10 µg/mL streptomycin, 4 µg/mL insulin (Sigma-Aldrich), 0.1% bovine serum albumin (BSA) (Eurobio), 0.5 µg/mL amphotericin B (Thermo Fisher Scientific), and 40 ng/mL dexamethasone (Sigma-Aldrich). Four hours later, the medium was replaced with the same medium lacking FBS, insulin, BSA and amphotericin B. For 3D culture, hepatocytes and NPC were resuspended in William's E medium with GlutaMAX™, supplemented with 10% FBS, penicillin/streptomycin, and 1% insulin-transferrin-selenium (ITS-G, Thermo Fisher Scientific).

Histology, IHC, TUNEL assay and multiplexed immunofluorescence. For IHC, liver sections were deparaffinized and rehydrated, and antigen retrieval was performed in citrate buffer (pH 6.0) for 20 min in a water bath at 95°C. Sections were blocked with TBS containing 0.1% Tween (TBST) and 5% normal goat serum (Vector Laboratories), incubated with the primary antibody for 1 h at room temperature or overnight at 4°C, depending on the antibody, washed twice with TBST, and then incubated with biotinylated secondary antibody. Immunoreactivity was detected using the VECTASTAIN® Elite ABC-HRP kit (Peroxidase; Vector Laboratories) and a 3,3′-diaminobenzidine substrate kit (Vector Laboratories). Nuclei were counterstained with Mayer's hemalun solution, and slides were dehydrated and mounted. For IHC analysis on organoids and tumouroids, samples were fixed for 1 h in 10% buffered formalin (VWR) at room temperature, washed with PBS, embedded in 2% agarose using a Leica mould, and subsequently processed for paraffin embedding. Detection of *in situ*

apoptosis was performed using the terminal deoxynucleotidyl transferase dUTP nick-end labeling (TUNEL) assay according to the manufacturer's instructions (Click-iT™ TUNEL Alexa Fluor™ 647, Thermo Fisher Scientific).

Multiplexed immunofluorescence was performed using the Comet™ instrument (Lunaphore) on 5-µm paraffin-embedded sections from $APC^{\Delta hep}$ non-tumour liver tissues, HCC, and tumouroids using a 12x12 mm chamber. Slides underwent deparaffinization followed by heat-induced epitope retrieval in alkaline buffer (pH 9.0) at a temperature reaching 102°C, followed by controlled cooling before subsequent staining steps. Tissues were subjected to 16 cycles of antibody incubation/elution and image acquisition with dedicated imaging, elution, quenching, and washing buffers provided by Lunaphore. One or two primary antibodies were incubated for 8 min, followed by incubation with one or two corresponding secondary antibodies for 4 min. Fields of view were saved, and background was subtracted using the HORIZON software (Lunaphore).

RNA sequencing (RNA-seq) and analysis. FASTQ files were aligned to the Ensembl release 101 reference genome using the STAR algorithm (version 2.7.6a). Reads were quantified with RSEM (v1.3.1), and differential expression analysis was performed in R (version 3.6.3) using the DESeq2 package (version 1.26.0). Gene counts were normalized using the standard DESeq2 normalization method (median-of-ratios implemented in the DESeq function) after pre-filtering reads and genes (reads uniquely mapped to the genome, or mapped to up to 10 loci with count adjustment, and genes with at least 10 reads in at least 3 samples). Differentially expressed genes were identified using the Wald test with the contrast function, and *P* values were adjusted for multiple testing using the Benjamini-Hochberg false discovery rate (FDR)

procedure, as recommended in the DESeq2 package documentation. R scripts and analysis parameters are available at https://github.com/GENOM-IC-Cochin/RNA-Seq_analysis. Raw sequencing data generated in this study are available in the European Nucleotide Archive (ENA) under accession number PRJEB115263.

GSEA analysis. GSEA was performed using GSEA software (Broad Institute, version 4.4.0). Genes were ranked according to their $\log_2$ fold change ($\log_2$FC) obtained from the differential expression analysis, and enrichment was assessed using the GSEA Pre-ranked algorithm. Statistical significance was determined using 1,000 gene set permutations. Nominal *P* values were adjusted for multiple testing using the false discovery rate (FDR) method. Gene sets with an FDR < 0.25 were considered significantly enriched, in accordance with the Broad Institute recommendations.

Supplementary figures

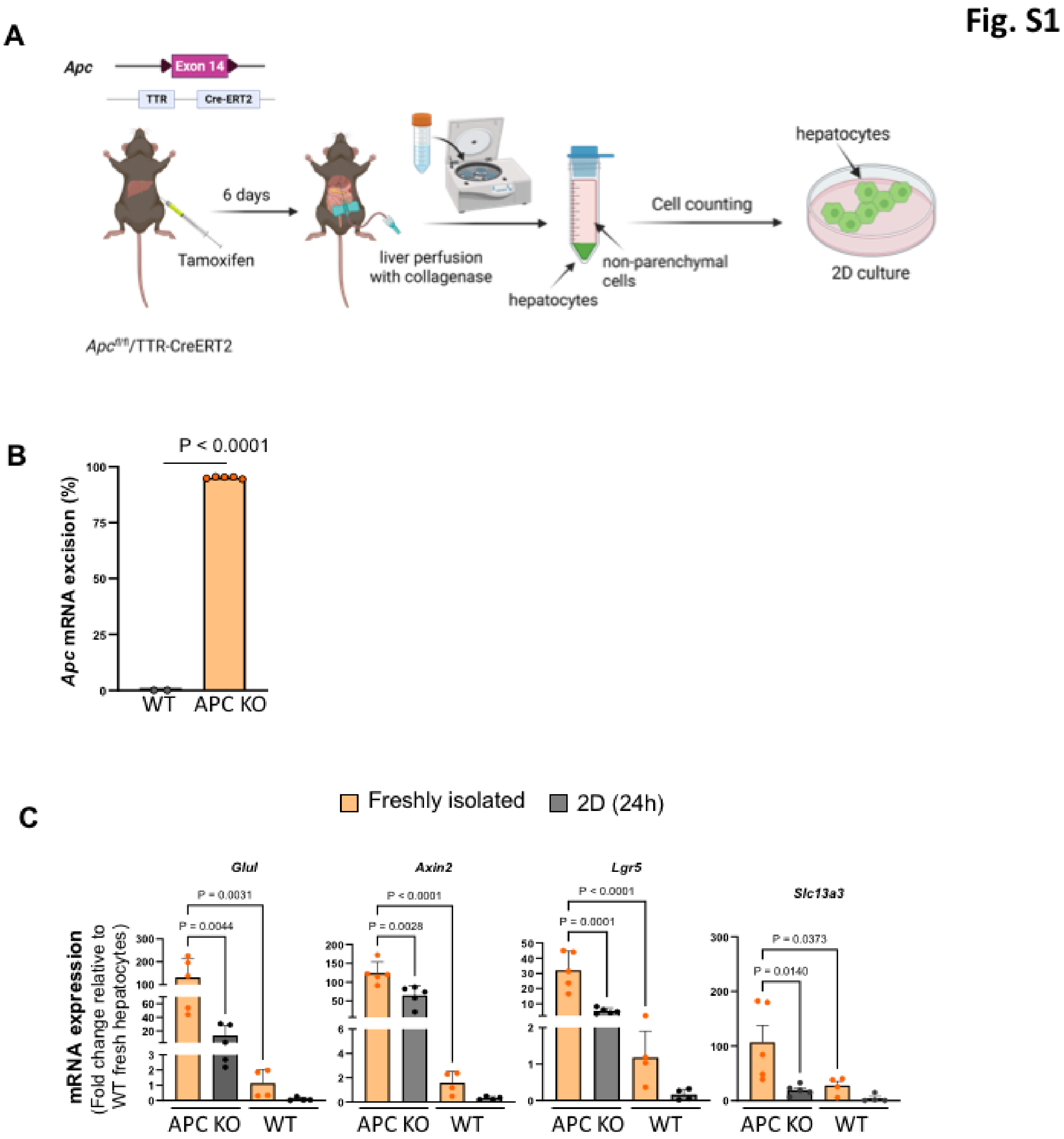

Fig. S1. 2D culture of APC^{KO} hepatocytes.

(A) Schematic illustration of the experimental design for the 2D culture of APC^{KO} hepatocytes (created with BioRender.com). **(B)** Percentage of *Apc* mRNA excision in freshly isolated wild-type (WT) and APC^{KO} hepatocytes determined by RT-qPCR (n = 5 biological replicates). **(C)** Relative transcript expression of *Glul*, *Axin2*, *Lgr5,* and *Slc13a3* assessed by RT-qPCR in freshly isolated APC^{KO} hepatocytes or after 24 h of

2D culture, normalized to WT freshly isolated hepatocytes (n = 5 biological replicates, one mouse per replicate). For each biological replicate, RNA was extracted from a pool of 20-25 organoids. Bar graphs represent mean values ± SD. Differences between two sample groups were assessed using the Mann-Whitney test. Differences among three sample groups were assessed using the Kruskal-Wallis test.

Fig. S2

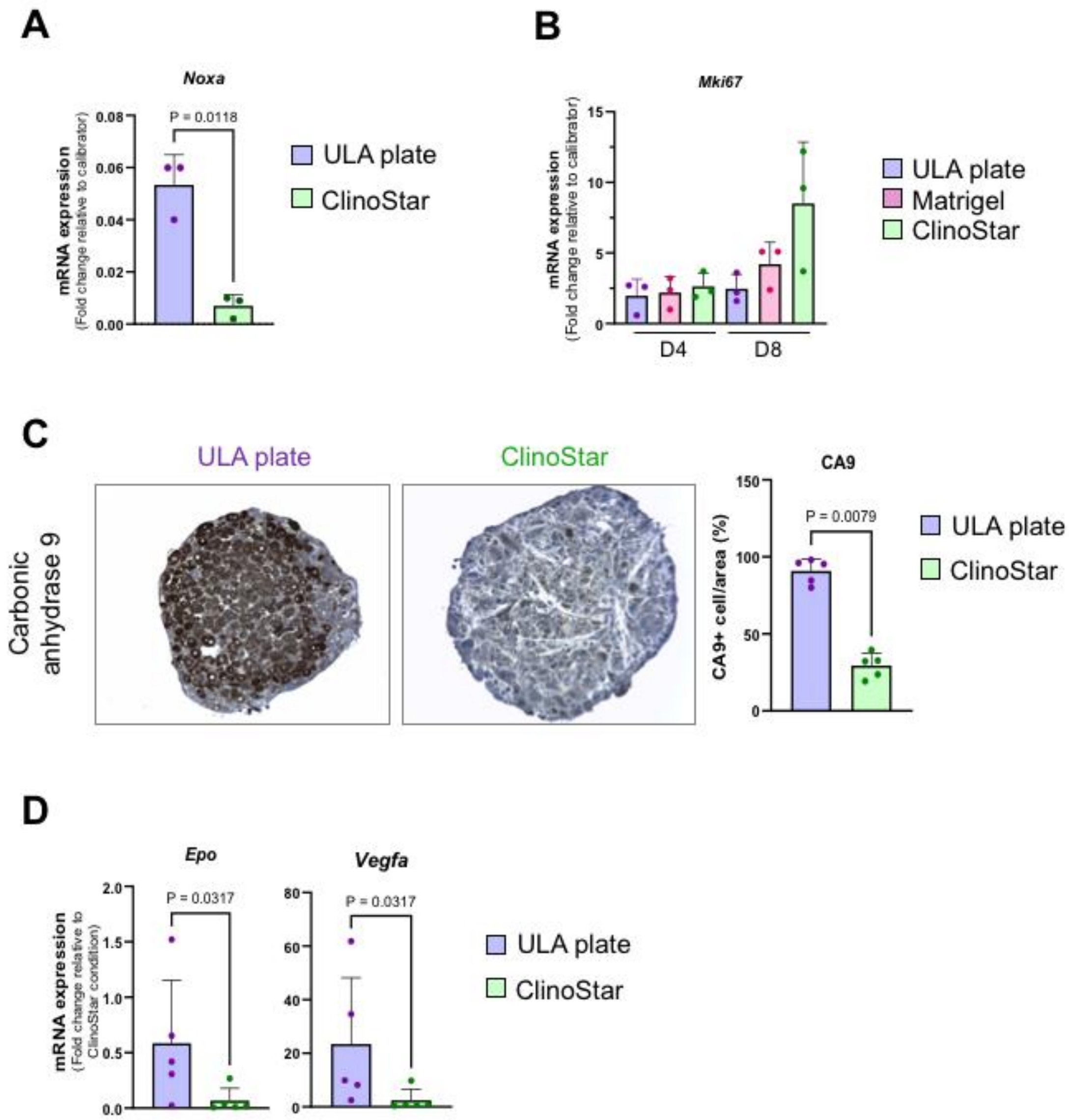

Fig. S2. Proliferation, apoptosis and hypoxia in APC$^{\Delta hep}$ liver organoids.

(A) Relative *Noxa* transcript expression assessed by RT-qPCR under the two culture conditions. Bar graphs show mean ± SD (n = 3 biological replicates, one mouse per replicate). For each biological replicate, RNA was extracted from a pool of 20-25 organoids. **(B)** Relative transcript expression of *Mki67* assessed by RT-qPCR in organoids cultured for 4 and 8 days in ULA plates, Matrigel or in the ClinoStar® system (n = 3 biological replicates, one mouse per replicate). For each biological replicate,

RNA was extracted from a pool of 20-25 organoids. Bar graphs represent mean values ± SD. **(C)** Representative immunostaining for carbonic anhydrase 9 (CA9) in $APC^{\Delta hep}$ liver organoids cultured for 8 days in ULA plates or in the ClinoStar® system (*left*), and quantification of CA9-positive cells (*right*) (n = 5 biological replicates, one mouse per replicate). For each biological replicate, 8-14 tumouroids were analyzed. **(D)** Relative transcript expression of *Epo* and *Vegfa*, assessed by RT-qPCR in organoids cultured for 8 days in ULA plates or in the ClinoStar® system (n = 5 biological replicates, one mouse per replicate). For each biological replicate, RNA was extracted from a pool of 20-25 organoids. Bar graphs represent mean values ± SD. Differences between groups were assessed using the Mann-Whitney test.

Fig. S3

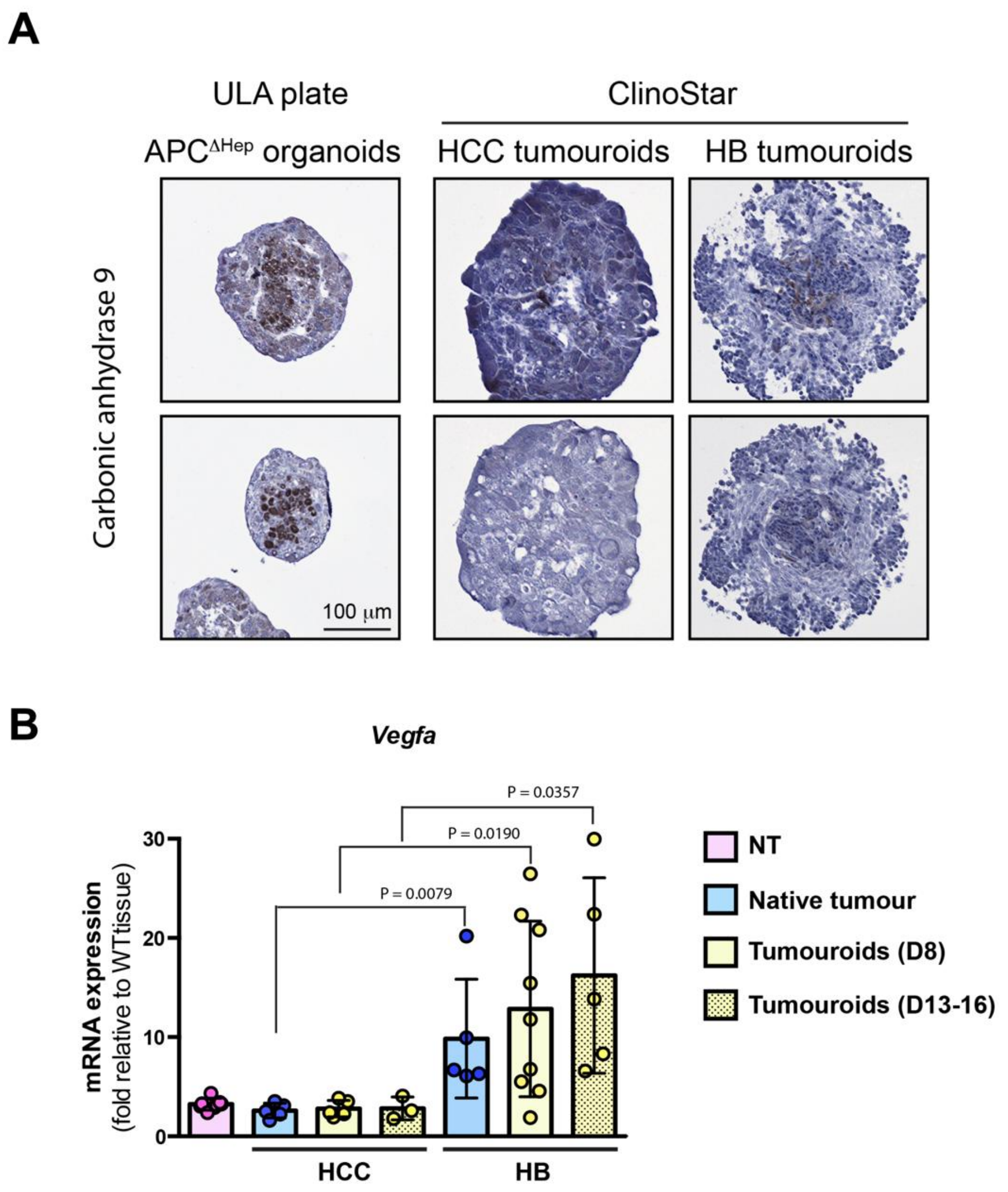

Fig. S3. Hypoxia in HCC and HB-liked tumouroids.

(A) Representative immunostaining for carbonic anhydrase 9 (CA9) in HCC and HB-liked tumouroids cultured during 16 days in the ClinoStar® system (n = 2 biological replicates, one mouse per replicate). For each biological replicate, 5-6 tumouroids were analyzed. As a positive control, APC$^{\Delta hep}$ liver organoids cultured in ULA plates are shown. **(B)** Relative transcript expression of *Vegfa*, assessed by RT-qPCR in non

tumour liver tissue (NT)s, HCC, HB, and HCC and HB-liked tumouroids cultured for 8 or 13-16 days in the ClinoStar® system (n = 3-9 biological replicates, one mouse per replicate). For each biological replicate, RNA was extracted from a pool of 20-25 organoids. Bar graphs represent mean values ± SD. Differences between groups were assessed using the Mann-Whitney test.

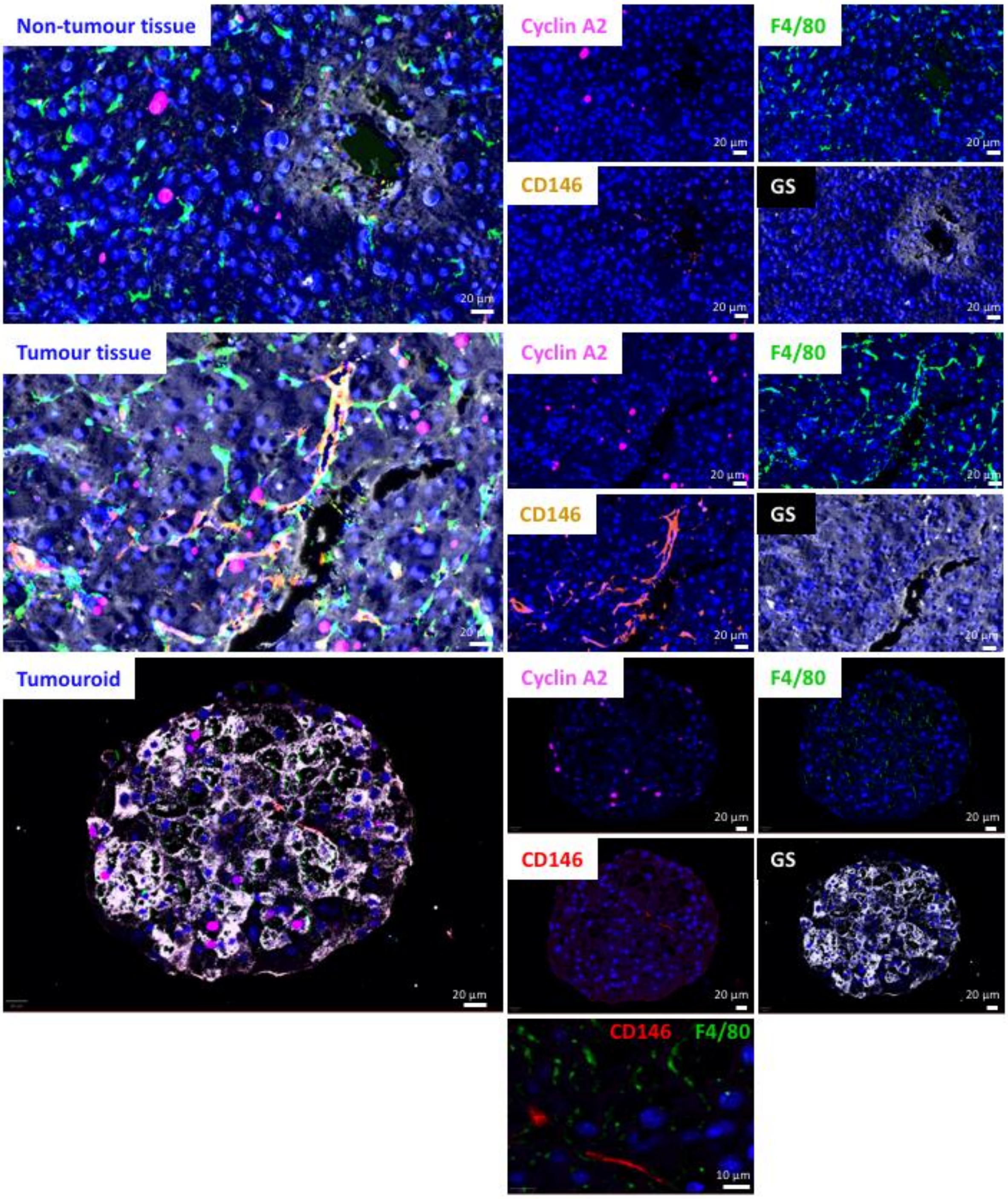

Fig. S4. Multiplex tissue profiling of HCC-derived tumouroids.

Representative images of multiplex immunofluorescence performed using COMET^{TM} on non-tumour liver tissue, HCC and HCC-derived tumouroid from APC$^{\Delta hep}$ mouse

showing cyclin A2 (magenta), CD146 (red), GS (white), F4/80 (green), and nuclei counterstained with DAPI (blue).

Fig. S5

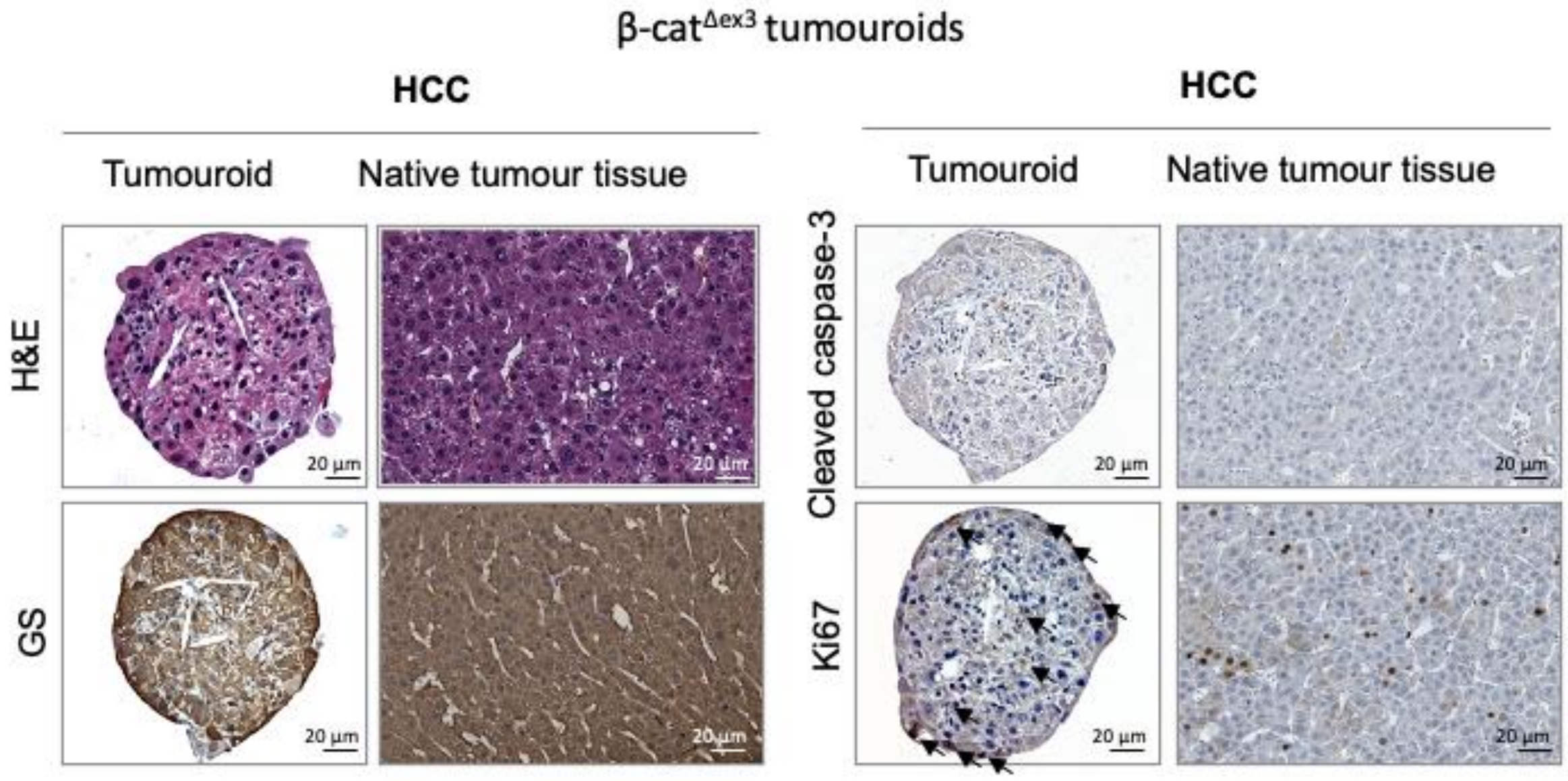

Fig.S5. Histological and IHC characterization of βcat$^{\Delta ex3}$ tumouroids.

Representative sections of H&E staining and immunostaining for cleaved capase-3, GS, and Ki67 in β-cat$^{\Delta ex3}$ tumouroids formed after 8 days of culture in the ClinoStar® system. Black arrows indicate Ki67-positive nuclei. n = 3 biological replicates, one mouse per replicate. For each biological replicate, 4-6 tumouroids were analyzed.

Fig. S6

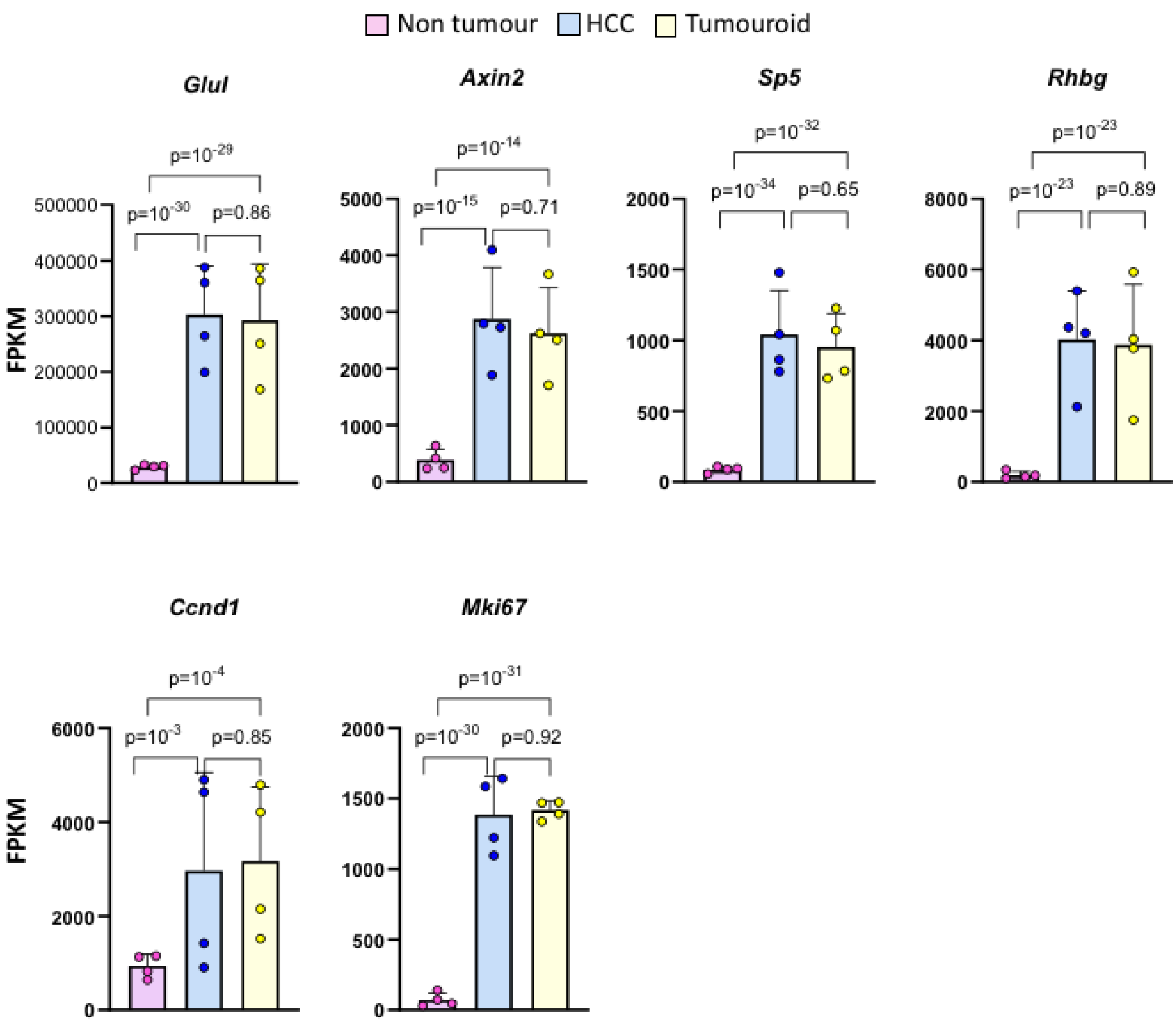

Fig. S6. Expression of proliferation- and β-catenin-related genes in HCC-derived APC$^{\Delta hep}$ tumouroids.

RNA-seq data (FPKM) showing the upregulation of *Glul*, *Axin2*, *Sp5*, *Rhbg*, *Ccnd1* and *Mki67* in murine HCC-derived APC$^{\Delta hep}$ tumouroids compared with adjacent non-tumour liver tissue and HCC**.** Differences among groups were assessed using the Kruskal-Wallis test.

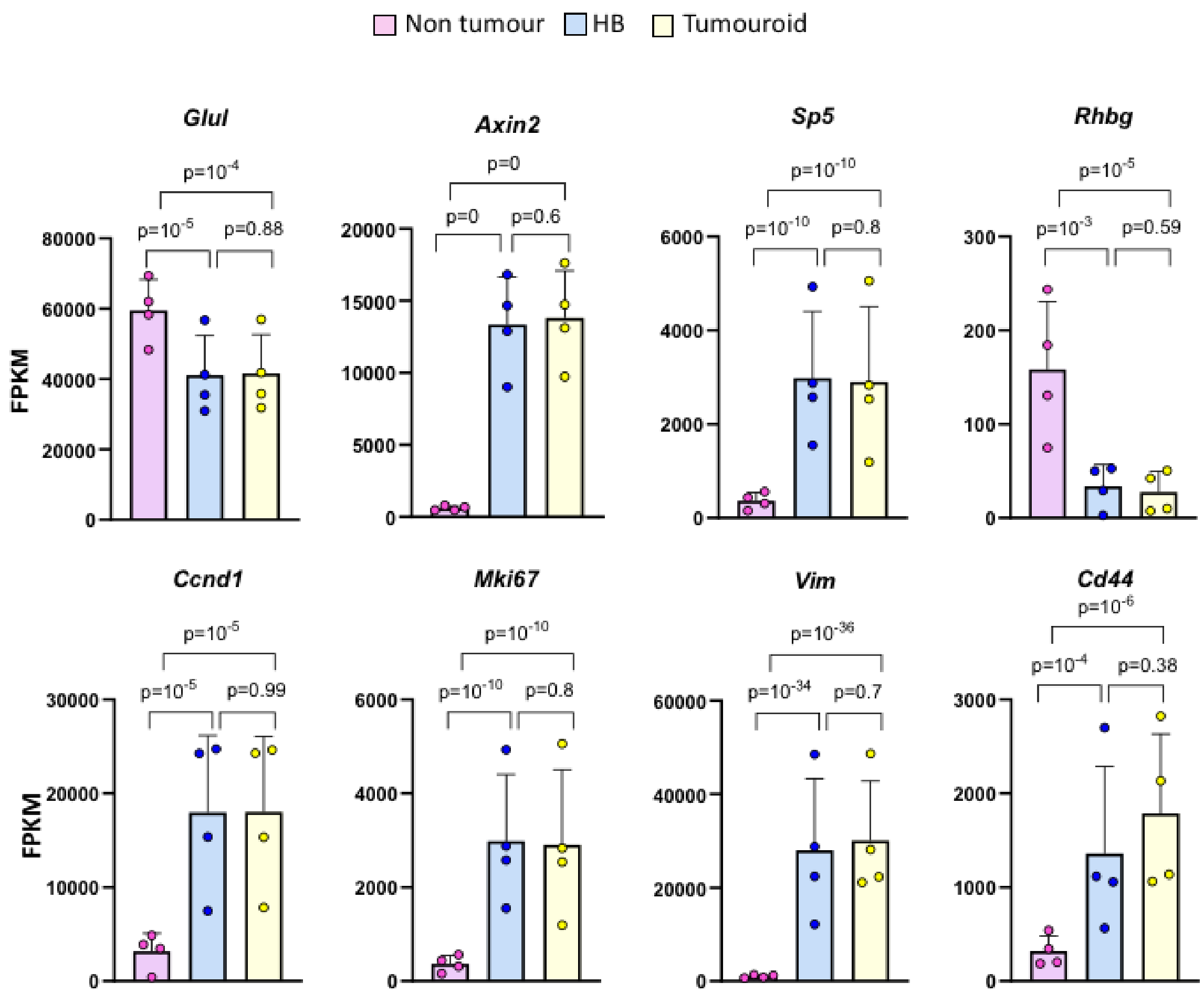

Fig. S7. Expression of proliferation-, β-catenin- and mesenchyme-related genes in HB-liked APC$^{\Delta hep}$ tumouroids.

RNA-seq data (FPKM) showing the status of *Glul*, *Axin2*, *Sp5*, *Rhbg*, *Ccnd1*, *Mki67, Vim* and *Cd44* in murine HB-liked APC$^{\Delta hep}$ tumouroids compared with adjacent non-tumour liver tissue and HB**.** Differences among groups were assessed using the Kruskal-Wallis test.

Fig. S8

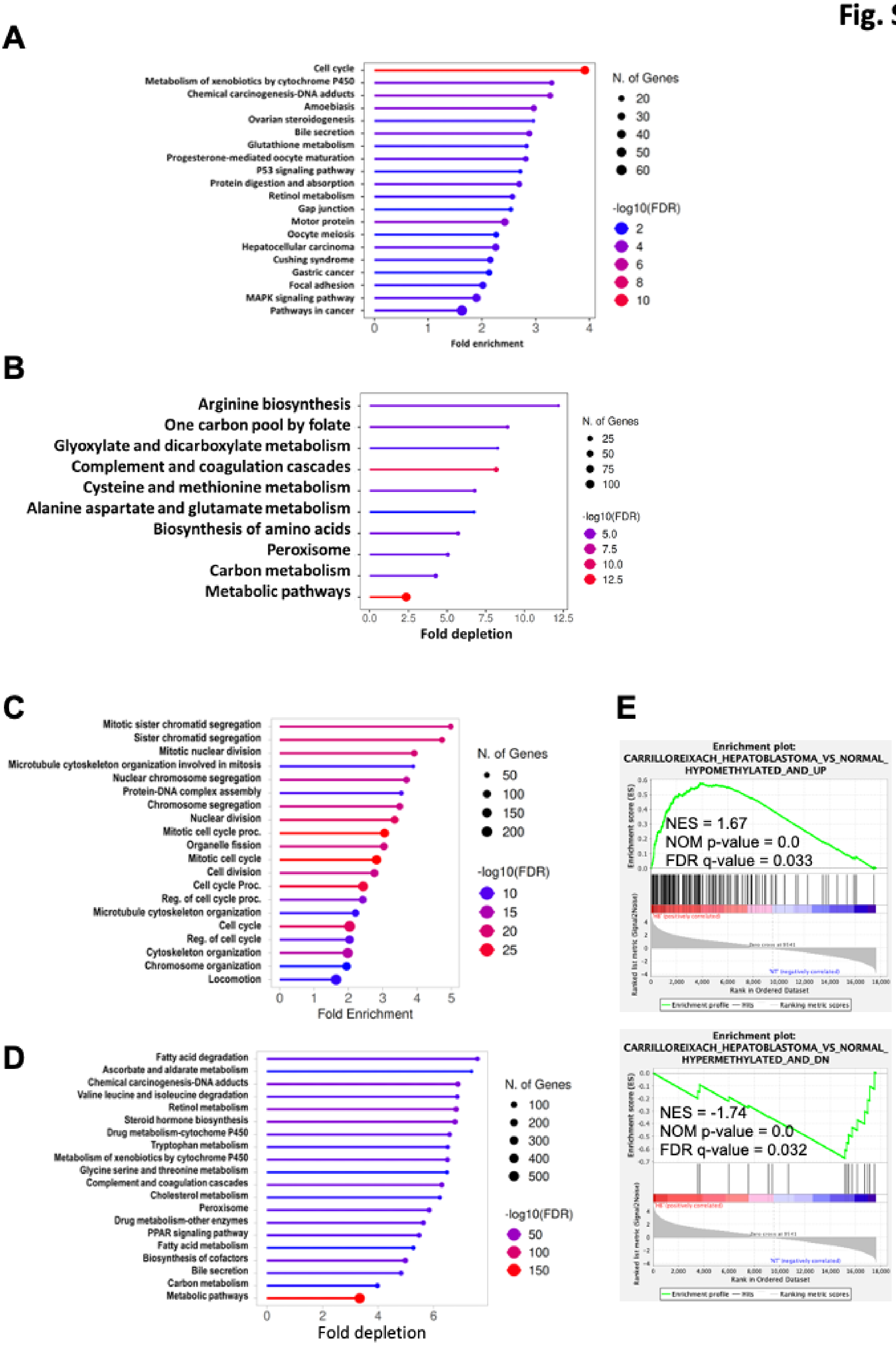
A
Cell cycle
Metabolism of xenobiotics by cytochrome P450
Chemical carcinogenesis-DNA adducts
Amoebiasis
Ovarian steroidogenesis
Bile secretion
Glutathione metabolism
Progesterone-mediated oocyte maturation
P53 signaling pathway
Protein digestion and absorption
Retinol metabolism
Gap junction
Motor protein
Oocyte meiosis
Hepatocellular carcinoma
Cushing syndrome
Gastric cancer
Focal adhesion
MAPK signaling pathway
Pathways in cancer
0 1 2 3 4
Fold enrichment
N. of Genes
20
30
40
50
60
-log10(FDR)
2
4
6
8
10
B
Arginine biosynthesis
One carbon pool by folate
Glyoxylate and dicarboxylate metabolism
Complement and coagulation cascades
Cysteine and methionine metabolism
Alanine aspartate and glutamate metabolism
Biosynthesis of amino acids
Peroxisome
Carbon metabolism
Metabolic pathways
0.0 2.5 5.0 7.5 10.0 12.5
Fold depletion
N. of Genes
25
50
75
100
-log10(FDR)
5.0
7.5
10.0
12.5
C
Mitotic sister chromatid segregation
Sister chromatid segregation
Mitotic nuclear division
Microtubule cytoskeleton organization involved in mitosis
Nuclear chromosome segregation
Protein-DNA complex assembly
Chromosome segregation
Nuclear division
Mitotic cell cycle proc.
Organelle fission
Mitotic cell cycle
Cell division
Cell cycle Proc.
Reg. of cell cycle proc.
Microtubule cytoskeleton organization
Cell cycle
Reg. of cell cycle
Cytoskeleton organization
Chromosome organization
Locomotion
0 1 2 3 4 5
Fold Enrichment
N. of Genes
50
100
150
200
-log10(FDR)
10
15
20
25
D
Fatty acid degradation
Ascorbate and aldarate metabolism
Chemical carcinogenesis-DNA adducts
Valine leucine and isoleucine degradation
Retinol metabolism
Steroid hormone biosynthesis
Drug metabolism-cytochrome P450
Tryptophan metabolism
Metabolism of xenobiotics by cytochrome P450
Glycine serine and threonine metabolism
Complement and coagulation cascades
Cholesterol metabolism
Peroxisome
Drug metabolism-other enzymes
PPAR signaling pathway
Fatty acid metabolism
Biosynthesis of cofactors
Bile secretion
Carbon metabolism
Metabolic pathways
0 2 4 6
Fold depletion
N. of Genes
100
200
300
400
500
-log10(FDR)
50
100
150
E
Enrichment plot:
CARRILLOREIXACH_HEPATOBLASTOMA_VS_NORMAL_HYPOMETHYLATED_AND_UP
NES = 1.67
NOM p-value = 0.0
FDR q-value = 0.033
Rank in Ordered Dataset
Enrichment plot:
CARRILLOREIXACH_HEPATOBLASTOMA_VS_NORMAL_HYPERMETHYLATED_AND_DN
NES = -1.74
NOM p-value = 0.0
FDR q-value = 0.032
Rank in Ordered Dataset

Fig. S8. KEGG pathway enrichment and GSEA analyses in murine HCC and HB-derived tumoroids

(A) KEGG pathway enrichment analysis of genes significantly upregulated in murine HCC-derived tumouroids compared to adjacent non-tumoural livers. **(B)** KEGG pathway enrichment analysis of genes significantly downregulated in HCC-derived tumouroids compared to adjacent non-tumoural livers. **(C)** KEGG pathway enrichment analysis of genes significantly upregulated in murine HB-derived tumouroids compared to adjacent non-tumoural livers. **(D)** KEGG pathway enrichment analysis of genes significantly downregulated in HB-liked tumouroids compared to adjacent non-tumoural livers. **(E)** GSEA highlighting significantly enriched signatures between murine tumouroids and human HB tissues. Enrichment results are shown for selected gene sets, with normalized enrichment scores (NES), nominal (NOM) p-values, and false discovery rate (FDR) q-values indicated.

Fig. S9

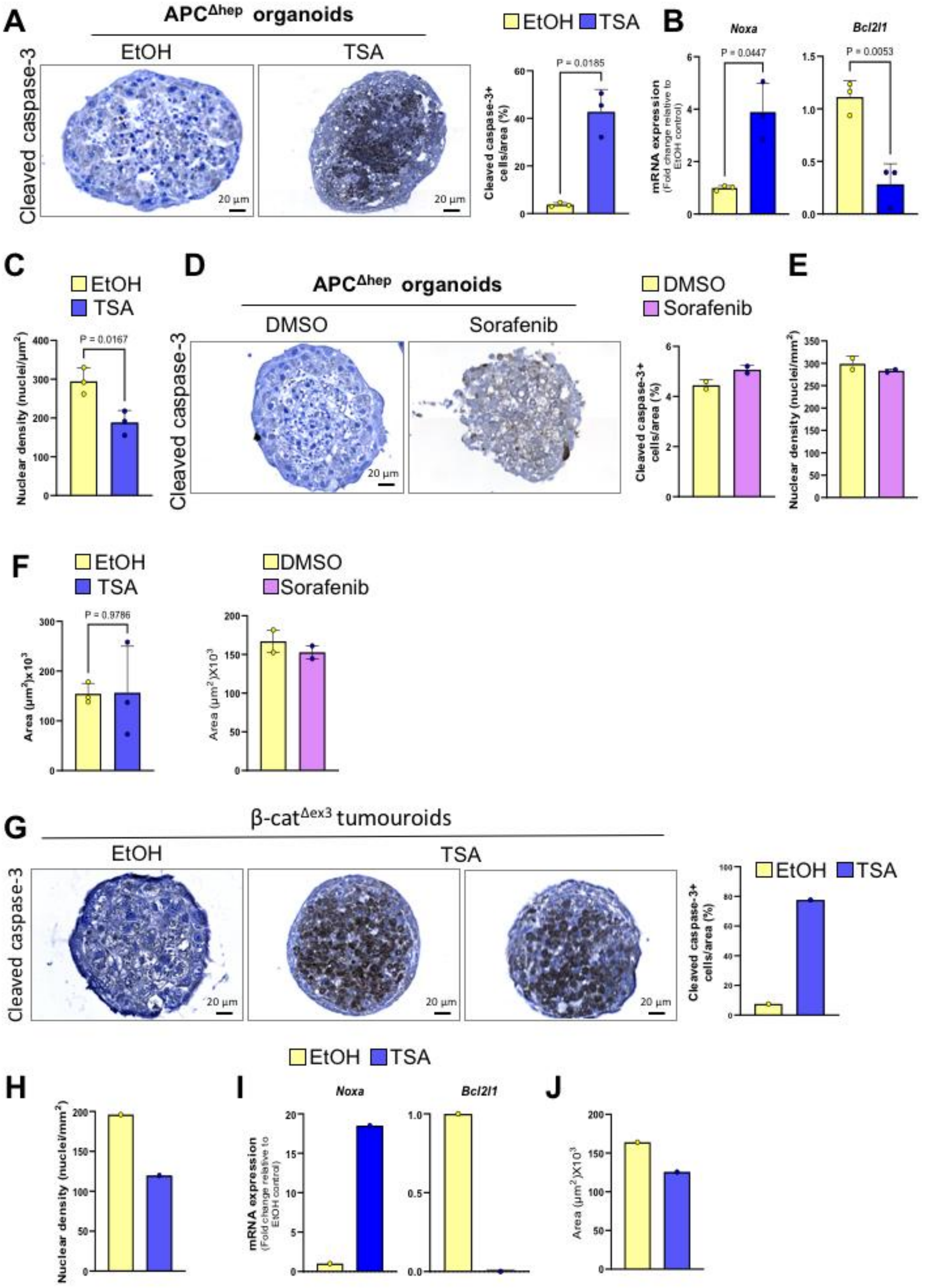
A
APC$^{\Delta hep}$ organoids
EtOH
TSA
Cleaved caspase-3
20 µm
EtOH TSA
P = 0.0185
Cleaved caspase-3+ cells/area (%)
B
Noxa
Bcl2l1
P = 0.0447
P = 0.0053
mRNA expression (Fold change relative to EtOH control)
C
EtOH
TSA
P = 0.0167
Nuclear density (nuclei/µm^2)
D
APC$^{\Delta hep}$ organoids
DMSO
Sorafenib
Cleaved caspase-3
DMSO
Sorafenib
Cleaved caspase-3+ cells/area (%)
E
Nuclear density (nuclei/mm^2)
F
EtOH
TSA
DMSO
Sorafenib
P = 0.9786
Area (µm^2)x10^3
Area (µm^2)X10^3
G
β-cat$^{\Delta ex3}$ tumouroids
EtOH
TSA
Cleaved caspase-3
EtOH TSA
Cleaved caspase-3+ cells/area (%)
EtOH TSA
H
Nuclear density (nuclei/mm^2)
I
Noxa
Bcl2l1
mRNA expression (Fold change relative to EtOH control)
J
Area (µm^2)X10^3

Fig.S9. Effects of TSA and sorafenib in APC$^{\Delta hep}$ liver organoids and βcat$^{\Delta ex3}$ HCC tumouroids.

(A) Representative immunostaining for cleaved caspase-3 in APC$^{\Delta hep}$ liver organoids generated after 8 days of culture in the ClinoStar® system and treated with trichostatin A (TSA, 0.5 µM) or ethanol (EtOH) for 5 additional days (*left*), with quantification (*right*) (n = 3 biological replicates, one mouse per replicate). For each biological replicate, 7-14 tumouroids were analyzed. **(B)** Relative *Noxa* and *Bcl2l1* transcript expression assessed by RT-qPCR in TSA- or vehicule-treated APC$^{\Delta hep}$ organoids (n = 3 biological replicates, one mouse per replicate). For each biological replicate, RNA was extracted from a pool of 20-25 organoids. **(C, E)** Quantification of nuclear density (n = 3 biological replicates, one mouse per replicate). For each biological replicate, 7-14 tumouroids were analyzed. **(D)** Representative immunostaining for cleaved caspase-3 in APC$^{\Delta hep}$ liver organoids generated after 8 days of culture in the ClinoStar® system and treated with sorafenib (5 µM) or DMSO for 5 additional days (*left*), with quantification (*right*) (n = 2 biological replicates, one mouse per replicate). For each biological replicate, 6-10 tumouroids were analyzed. **(F)** Quantification of projected organoid area (n = 2-3 biological replicates, one mouse per replicate). For each biological replicate, 6-10 tumouroids were analyzed. **(G)** Representative immunostaining for cleaved caspase-3 in HCC tumouroids generated after 8 days of culture in the ClinoStar® and treated with trichostatin A (TSA, 0.5 µM) or ethanol (EtOH) for 5 days (*left*), with quantification (*right*) (n = 1 mouse). A total of 15 tumouroids was analyzed. **(H)** Quantification of nuclear density (n = 1 mouse). A total of 15 tumouroids were analyzed. **(I)** Relative *Noxa* and *Bcl2l1* transcript expression assessed by RT-qPCR in TSA- or ethanol-treated βcat$^{\Delta ex3}$ HCC tumouroids (n = 1 mouse). RNA was extracted from a pool of 20-25 organoids. **(J)** Quantification of projected organoid area (n = 1 mouse). 15

tumouroids were analyzed. Bar graphs represent mean values ± SD. Differences between groups were assessed using the Mann-Whitney test.

Fig. S10

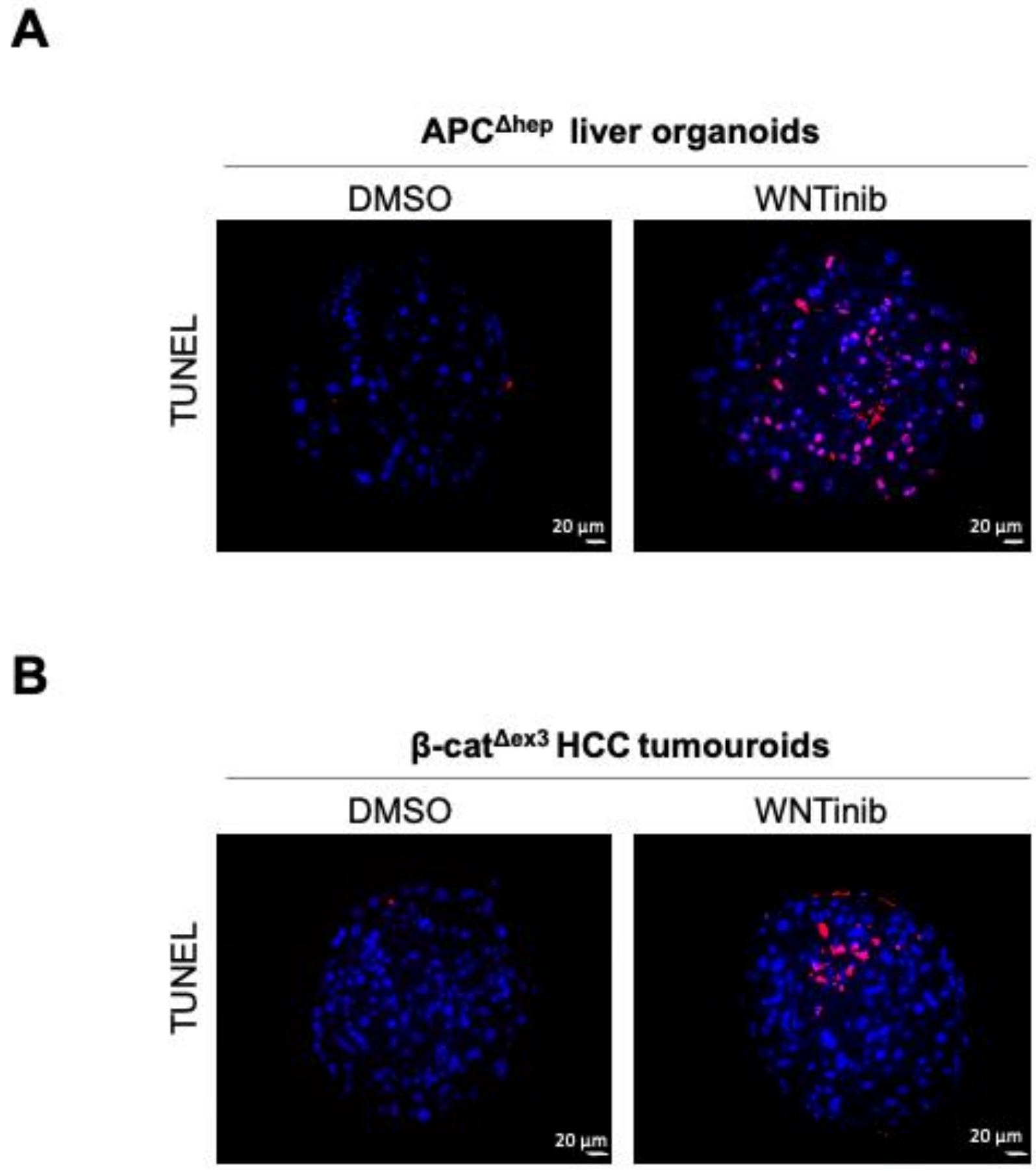

Fig.S10. TUNEL assay in Apc$^{\Delta hep}$ liver organoids and βcat$^{\Delta ex3}$ HCC tumouroids.

(A) Representative images of Apc$^{\Delta hep}$ liver organoids cultured during 8 days in the ClinoStar® system and treated with WNTinib (1 µM) or DMSO for 5 days. Apoptotic cells were detected by TUNEL staining (red), with nuclei counterstained with DAPI (blue) (n = 2 biological replicates, one mouse per replicate). For each biological replicate, 5 tumouroids were analyzed. **(B)** Representative images of β-cat$^{\Delta ex3}$ HCC tumouroids cultured during 8 days in the ClinoStar® system and treated with WNTinib (5 µM) or DMSO for 5 days. Apoptotic cells were detected by TUNEL staining (red), with nuclei counterstained with DAPI (blue) (n = 2 biological replicates, one mouse per replicate). For each biological replicate, 5-6 tumouroids were analyzed.

Supplementary tables

Table S1: Characteristics of $APC^{\Delta hep}$ tumours used for tumouroid generation and transcriptomics

Name	Type	Cumulative tumour diameter (mm) measured by ultrasound at sacrifice	Tumouroid number
TUM#1	HCC	12 * 11<br>hemorrhagic and necrotic areas	30
TUM#2	HCC	11 * 10	46
TUM#4G	HCC	6 * 6	41
TUM#4B	HCC	6 * 5	35
TUM#5	HB-like	10 * 8.5	32
TUM#7	HCC	8.5 * 8	28
TUM#9	HB-like	7 * 6.5	38
TUM#14	HB-like	12 * 10	63
TUM1#5	HB-like	11 * 10	75

Following tumour excision, one fragment was processed for histological analyses, a second fragment (~20 mg) was snap-frozen and stored at -80°C for RNA extraction, and the remaining tissue was used for tumouroid generation.

Table S2: Murine gene signatures generated for GSEA.

Human gene signature	Gene number	Reference	Murine gene list	Gene number
13_GENE_MBGS	13	43	*Slc1a2*, *Notum*, *Slc13a3*, *Lgr5*, *Axin2*, *Nkd1*, *Tcf7*, *Sp5*, *Rhbg*, *Glul*, *Tnfrsf19*, *Teddm1*, *Sbspon*	13
BOYAULT_LIVER_CANCER_SUBCLASS_G6_UP	66	40	*Acsl6*, *Acss3*, *Actn2*, *Ap3s1*, *Aspscr1*, *Atp6v0e2*, *Bcap31*, *Bmp4*, *Bpnt2*, *C1qtnf3*, *Cbr1*, *Ccdc170*, *Cemip2*, *Col7a1*, *Cryz*, *Ctnna2*, *Ctnnbl1*, *Dync1i1*, *Efr3a*, *Fbxl5*, *Fhit*, *Galnt1*, *Ggh*, *Greb1*, *Hexa*, *Hsdl2*, *Itpr2*, *Lef1*, *Lgals8*, *Lpin1*, *Mertk*, *Mlec*, *Mme*, *Nek3*, *Npr2*, *Panx1*, *Parvb*, *Preb*, *Rab4a*, *Rhobtb1*, *Rnf43*, *Samm50*, *Septin4*, *Siah2*, *Slc13a3*, *Slc22a4*, *Slc5a6*, *Slc6a2*, *Sult4a1*, *Tbca*, *Tceal1*, *Thsd1*, *Timm17a*, *Tmcc1*, *Trib2*, *Ttc9*, *Uggt1*, *Vsnl1*, *Vstm4*, *Washc3*, *Ypel1, Zbtb18*	62
LACHENMAYER Wnt-CTNNB1	17	42	*Nkd1, Axin2, Rock2, Sall1, Tle1, Dvl2, Ctnnbip1, Smad3, Tcf7, Brd7, Daam1, Cul1, Ppp3cb, Dlg1, Ruvbl1, Tbl1xr1, Senp2*	17
CHIANG_LIVER_CANCER_SUBCLASS_CTNNB1_UP	181	41	*Aaadac, Abcb11, Abcg2, Abhd6, Ace2, Acsl5, Acsl6, Acsf3, Actn2, Adh6a, Adrb2, Aldh1l1, Aldh3a1, Aldh3a2, Amacr, Ankfn1, Aox1, Aqp11, Aqp6, Aqp9, Ar, Asap2, Aspscr1, Axin2, Bambi, Bhlhe40, Bik, Bmp4, Bok, Cap2, Cavin2, Ccdc170, Cd36, Cdc14b, Cdk6, Cldn2, Corin, Cpped1, Crls1, Cst1, Ctnna2, Ctnnbl1, Cyp1a1, Cyp2e1, Cyp8b1, Dnajc12, Dpp4, Dsg1a, Ebpl, Ecm2, Ephb2, Esrrg, Exph5, Fam169a, Fam3b, Fam8a1, Fas, Fgf13, Fitm2, Frmd3, Gfra1, Glul, Glyat, Gnai1, Gpam, Gphn, Grhpr, Grk3, Gstm2, Habp4, Hepacam, Hhat, Hibadh, Hlf, Hoga1, Hpd, Hsd11b1, Hsdl2, Htr2b, Ift70a, Insig2, Irs1, Irx3, Itpr2, Kcnj8, Kcnk1, Lgr5, Map3k8, Mertk, Mme, Mthfd1, Myrip, Nags, Nek3, Nkd1, Nubpl, Nudt6, Panx1, Pdk1, Pdk4,*	177

			Phlpp1, Plaat2, Plppr1, Prag1, Preb, Prr5l, Ptprg, Rab11fip2, Rbp1, Reg1a, Reg3a, Rhbg, Rhobtb1, Rtp3, Sall1, Sec14l1, Selenbp1, Septin4, Shld2, Slc13a3, Slc16a1, Slc16a10, Slc16a11, Slc16a4, Slc17a1, Slc1a2, Slc22a11, Slc22a4, Slc25a30, Slc2a12, Slc47a1, Slc4a4, Slc5a6, Slc6a12, Slco1b2, Smpx, Snai2, Sparcl1, Spryd7, Srd5a2, Sult1b1, Tbc1d15, Tbck, Tbx3, Tenm2, Thbs4, Tmem100, Tmem150c, Tmem245, Tmem64, Tnfrsf19, Tprg1, Trib2, Tspan5, Ttc9, Ttpa, Ubxn10, Ust, Vegfd, Vldlr, Washc3, Zfp385b, Znrf3	
CAIRO_HEPATOBLASTOMA_UP	204	10	*Acaca, Acly, Acss3, Akr1c3, Alcam, Angel2, Ankrd27, Arl4a, Asap2, Aspscr1, Avl9, Bambi, Bcam, Bex1, Brd3, Camsap2, Cap2, Cbx1, Cbx5, Cbx6, Cct3, Cd24, Cd24p2, Cd46, Cdc23, Cdk4, Cep68, Cetn2, Ckap4, Ckb, Clic1, Clmn, Col15a1, Coq8a, Cpd, Creb3l2, Crim1, Cripto, Cs, Cyp51a1, Dcp2, Ddhd2, Denr, Dkk1, Dlg5, Dlk1, Dram1, Dzip3, Ehmt2, Elp1, Emcn, Epcam, Erg28, Etv1, Fasn, Fdft1, Fdps, Fkbp1b, Fnbp1l, Frrs1l, Fxr1, Gabbr1, Gja1, Glul, Gmnn, Gnpat, Gorasp2, Gpc3, Greb1, Gsta4, Hdac2, Heatr1, Hip1, Hmga2, Hmgcr, Hmgcs1, Hmgn4, Hsp90ab1, Hspa2, Hspb1, Igf2bp2, Ilf2, Impdh2, Itga6, Itgae, Jarid2, Kdm3b, Kdm5b, Khdc4, Lamc1, Lef1, Lgr5, Lrrc1, Lyz, Maged1, Maged2, Map3k4, Map4k4, Marchf8, Marcks, Mavs, Mcam, Mcm3, Meg3, Mff, Minpp1, Mlec, Mmp11, Mpzl1, Mrps27, Mtr, Nap1l1, Ncoa3, Ndn, Nsdhl, Nudt3, Nup133, Nup37, Obsl1, Palld, Papss1, Pdcd2, Pdgfa, Pdxk, Pdzk1, Peg10, Peg3, Pfkm, Piezo2, Plag1, Plppr1, Plvap, Plxnc1, Pms1, Podxl, Polr1d, Pomk, Ppp1cc, Ppp1r2, Prkca, Prkd1, Prr15l, Prune1, Psmd10, Psmd4, R3hdm1, Rab4a, Racgap1,*	204

			Rangrf, Rap2a, Rbm12, Rbm34, Rc3h 2, Rcor3, Reln, Rhobtb1, Rnf38, Rnf4 3, Robo1, Rock2, Rpa1, Rps6kc1, Rrp 1b, Sac3d1, Scd, Sephs1, Septin2, Se ptin8, Serpine2, Slc1a4, Slc25a36, Sl c26a2, Slf2, Smarca4, Smarcc1, Smy d2, Smyd3, Snhg32, Socs5, Sox4, Sp3 , Sqle, Srebf2, Suco, Tacc1, Tarbp1, T ax1bp3, Tbc1d9, Tbce, Tbx3, Tia1, T mem243, Tnks, Tomm20, Tor3a, Tp53 , Trib2, Tsn, Tspyl4, Tuft1, Tulp4, Uba p2, Ubap2l, Uggt1, Unc119b, Utrn, Uxs1, Vps45, Vwf, Wfs1, Zbtb38, Zfta , Znf189	
CAIRO_HEPATOBLASTOM A_DN	286	10	*Aass, Abat, Abcb11, Abhd2, Ablim3, Acaa1a, Acaa2, Acacb, Acadl, Acad m, Acads, Acat1, Acsl1, Adgra3, Adh 1, Adk, Adm, Agl, Agpat2, Agxt, Akr1 d1, Alas1, Aldob, Alpl, Ang, Angptl4, Ano1, Anxa10, Apof, Aqp3, Arhgef26, Asl, Ass1, Azgp1, Bbox1, Bmerb1, C 1r, C1rl, C6, C8a, C8b, C8g, C9, Ca 2, Cbs, Cd14, Cda, Cdc37l1, Cfp, Ch st7, Cideb, Clec1b, Clec4m, Cpeb3, Cpt2, Crem, Crhbp, Crp, Cth, Ctps1, Cxcl14, Cyp1a2, Cyp26a1, Cyp39a1, Cyp3a13, Cyp4f14, Dao, Dcps, Ddt, Dhodh, Dhrs1, Dhtkd1, Dnase1l3, Ec m1, Ehd3, Endog, Eno3, Epha2, Etfd h, Etnppl, Ets2, Ext1, F11, F9, Faah, Fahd2a, Fam149a, Fbp1, Fcgr2b, Fe tub, Foxo1, Fxyd1, Gadd45g, Gba3, Gcdh, Gcgr, Gch1, Ghr, Gls2, Glyat, Gne, Gnmt, Got1, Gpd1, Gpld1, Gpr 88, Gramdc1, Gramdc4, Grem2, Gstt 2, Gstz1, Gys2, Haao, Habp2, Hagh, Hal, Hao2, Hgd, Hgfac, Hp, Hpgd, H px, Hrg, Hsd17b6, Id2, Igf1, Igfals, I gfbp3, Il13ra2, Il1rap, Il4r, Inhbb, In pp1, Insig1, Itih4, Kcnd3, Kdm8, Klf6 , Kmo, Lcat, Lepr, Lonp2, Lpa, Lpin2 , Lyve1, Man1c1, Map2k3, Map3k14, Map3k5, Marco, Masp2, Mat1a, Mcl 1, Mfap3l, Mgll, Mmut, Mpc1, Mt1,*	273

			Myom1, N4bp2l1, Nampt, Nat2, Nfil3, Nnmt, Nnt, Nqo2, Nr1i2, Nr1i3, Nrg1, Ogdhl, Osbpl1a, Papss2, Pck1, Pck2, Pdlim5, Pemt, Pfkfb1, Plaat3, Plg, Pon1, Ppargc1a, Ppp1r1a, Prg4, Prodh, Pth1r, Pts, Pxdc1, Pxmp2, Pzp, Rab27a, Rcl1, Rdh16, Retreg1, Rida, Rnd3, Rnf125, Saa4, Sardh, Scp2, Sdhb, Sds, Serpinb8, Serpine1, Serping1, Shbg, Shmt1, Sigirr, Skap1, Slc10a1, Slc1a1, Slc20a1, Slc22a1, Slc22a7, Slc25a15, Slc27a2, Slc27a5, Slc37a4, Slc38a4, Slc3a1, Slc43a3, Slc46a3, Slpi, Socs2, Sorl1, Srd5a1, Srd5a2, St3gal6, Stab1, Stab2, Stard5, Suclg2, Syt17, Tdo2, Tenm1, Tent5a, Tfr2, Tmem131l, Tmprss2, Trib1, Tsku, Tspyl5, Ugp2, Ugt1a8, Vipr1, Vnn1, Vsig4, Xdh, Zc3h12a, Zfand5, Zg16, Zgpat	
CARRILLOREIXACH_HEPATOBLASTOMA_VS_NORMAL_HYPOMETHYLATED_AND_UP	162	44	*Acsl4, Adam9, Angpt1, Antxr1, Anxa1, Arid3a, Asap2, Axin2, B3galt2, Bambi, Bcam, Bcat1, Bcl9, Blmh, Bmp4, Cacnb4, Cd34, Cdh11, Cdh13, Cdk1, Cdk4, Cdk6, Cdkn3, Chd3, Chst11, Ckap2l, Clic1, Col12a1, Col15a1, Col1a1, Col1a2, Col2a1, Col4a1, Col4a2, Col6a3, Cpa6, Creb3l2, Depdc1, Dhx9, Dlgap5, Dnmt3a, Dpep1, Dtl, Dync1h1, Edil3, Esm1, F13a1, Fap, Fbln1, Fmnl2, Foxm1, Gas2l3, Gls, Gna12, Gramd1a, Greb1, Grk3, Hip1, Hk2, Hmgb2, Hnrnpa3, Hspg2, Htr1d, Hunk, Igf2bp1, Igf2bp2, Igsf1, Ilf3, Inpp5d, Itga2, Itga8, Kcnu1, Kif18a, Kit, Lama4, Lamb1, Lamc1, Lgr5, Map1b, Map4k4, Mctp1, Mgat5, Mgp, Mme, Mmp16, Ms4a2, Mtbp, Myo1d, Myo5a, Ncapd2, Ncapg, Ncaph, Nkd1, Notch3, Npm1, Nrp2, Nt5dc2, Nuak1, Nup93, Odam, Olfml2b, Olr1, Parpbp, Pdgfrb, Phyhipl, Piezo2, Plcg1, Pld3, Plxnc1, Prc1, Prkaa2, Prkdc, Ptk7, Ptpn14, R3hdm1, Racgap1, Rap2a, Rasgrf2, Rere, Rhobtb1, Rnas*	161

			e1, Robo1, Rpl8, Rps12, Rps3a, Rrm2, Sema3c, Sgo1, Slc1a5, Slc38a11, Slc7a11, Slc7a6, Slit3, Smarcc1, Smoc2, Sntg1, Spp1, Spry1, Stc1, Stk39, Sulf1, Tacc1, Tbx3, Tcf4, Tgfb2, Thbs2, Tmc7, Tnc, Tnfrsf19, Trib2, Trim71, Trps1, Tspan13, Txnip, Uaca, Uggt1, Vcan, Vwf, Zc3hav1l, Znrf3, Zswim5	
CARRILLOREIXACH_HEPATOBLASTOMA_VS_NORMAL_HYPERMETHYLATED_AND_DN	21	44	*A1bg, Acsl1, Adamtsl3, Akr7a3, Akr7l, Clybl, Ctps1, Cyp1a1, Cyp7b1, Ednrb, Esrp2, Hal, Hjv, Mt1l, Pemt, Per3, Plek2, Rhod, Slc43a3, Tbx15, Tspyl5*	21
WU_MESENCHYMAL_SPECIFIC_HEPATOBLASTOMA		28	*Sparc, Mef2c, Bgn, Col3a1, Timp2, Col5a1, Pdgfra, Col1a1, Dcn, Dnm3os, Cd9, Col1a2, Anxa1, Mgp, Igfbp7, Ifi6, Plxdc2, Eln, Itm2b, Calm1, Pls3, Nfic, Cybrd1, Timp3, Ano1, Fxyd6, Ahnak, Cd81, Eng, Col6a1, Egr1, Zcchc24, Junb, Vim, Col12a1, Anxa5, Ppp1r12b, Ids, Lpar1, Aebp1, Tagln, Emilin1, Kalrn, C1r, Mmp14, Ecm1, Lmna, Fstl1, Col8a1, S100a6*	50
WU_EMBRYONAL_SPECIFIC_HEPATOBLASTOMA		28	*Ambn, Mdfi, Mex3a, Vcan, Mmp16, Dach1, Sox4, Tyro3, Fbln2, Lnpep, Ibsp, Nap1l1, Rcn2, Tpbg, Gnas, Cxcl14, Nptx1, Laptm4b, Crabp2, Fkbp10, Tead2, Ptch1, Postn, Marcksl1, Sohlh2, Msx1, Frem2, Hnrnpa2b1, Ilf3, Ckap4, Cpxm1, Mycn, Phf6, Xrcc5, Hnrnpu, Slc34a2, Dbn1, Gpr161, Birc5, Tp53bp1, Wls, Fam84a, Axin2, Elmo1, Rasl11b, Vangl2, Hdac2, Hmga2*	49
SONG_FIBROBLAST_LIKE HEPATOBLASTOMA	145	27	*Col3a1, Col1a2, Col1a1, Postn, Cxcl14, Lum, Sparc, Dcn, Col6a3, Igfbp5, Fstl1, Col5a2, Prrx1, Col6a2, Ccdc80, Col14a1, Ogn, Ptn, Mgp, Ctsk, Igfbp3, Fbln1, Col6a1, Sfrp2, Aspn, Col5a1, Col12a1, Dpt, Emp1, Col16a1, Fbn1, Pcolce, Col2a1, Aebp1, Nnmt, Mtrnr2l1, Htra1, Igf1, Ltbp1, Prss23, Adamts1, Col11a1, Rnd3, Ptrf, Antxr1, Frzb, Lama4, Vwf, Angptl1, Cpe, Thbs2, Ppap2b, Cald1, Cybrd1, Timp3, Lamb1, Ctgf, Igfbp7, Defa3,*	142

			Crispld2, Wnt5a, Lgals1, Lama2, Spon1, Wwtr1, Tcf4, Il11ra, Palld, Timp2, Eln, Akap12, Igfbp4, Col4a1, Vcan, Col4a2, Thy1, Cyr61, Mxra5, Mmp2, Ednra, Moxd1, Cdh11, Sept11, Lrrc17, Fbln2, Cav1, Egfl6, Nfib, Gsn, Olfml3, Adamts9, Vim, Pxdn, Arid5b, Cpxm1, Fgf7, Ifi16, Kctd12, Abi3bp, Inhba, Comp, Zfp36l1, Calu, Emilin1, Col7a1, Slit3, Lox, Gng11, Mmp14, Fam150b, Kcne4, Mmrn1, Mxra8, Fmod, Ppic, Nbl1, Lrrc15, Sh3pxd2a, Sfrp1, Adamts2, Rpl9, Pdgfrb, Sulf1, Gja1, Adam33, Adirf, Anxa2, Tm4sf1, Calcrl, C1r, Bicc1, Serpinh1, Sesn3, Fibin, Serpine1, Fkbp7, Ttc3, Spry1, Twist1, Sept2, Id3, C1qtnf5, Omd	

Table S3: P values for heatmap from Figure 5C

Gene name	HCC vs NT	Tumouroid vs NT	Tumouroid vs HCC
Nkd1	8.41E-79	9.43E-78	0.89
Axin2	2.24E-15	3.83E-14	0.71
Lgr5	1.32E-08	6.81E-08	0.77
SP5	3.83E-34	6.81E-32	0.65
Znrf3	2.29E-06	6.72E-06	0.82
Lef1	2.78E-12	2.44E-12	0.98
Notum	3.52E-13	3.15E-12	0.76
Rnf43	5.94E-08	3.54E-07	0.74
Tnfrsf19	7.97E-10	8.54E-10	0.99
Slc13a3	3.38E-36	2.15E-45	0.89
Trib2	5.23E-10	1.46E-09	0.86
Lect2	4.17E-08	1.65E-07	0.8
Oat	1.66E-07	1.51E-06	0.67
Tbx3	3.39E-05	8.71E-05	0.82
Slc1a2	7.02E-18	7.58E-17	0.78
Glul	6.26E-30	4.42E-29	0.86
Rhbg	1.19E-23	4.21E-23	0.89
Aldh3a2	1.94E-07	7.18E-07	0.8
Aox1	5.55E-09	4.15E-08	0.72
Amacr	6.00E-03	6.00E-03	0.98
Abcc12	1.00E-02	1.00E-02	0.98
Abcc4	1.17E-30	5.51E-31	0.94
Cyp2a5	4.64E-09	5.88E-09	0.96
Ccna2	2.80E-37	6.41E-37	0.93
Mki67	1.15E-30	4.02E-31	0.92
E2f1	2.15E-15	5.96E-17	0.66
Nek2	3.60E-39	2.39E-39	0.97
Cd34	8.24E-39	2.30E-08	0.85
Mcam	1.43E-08	1.52E-06	0.38
Nqo1	9.78E-15	3.84E-13	0.62
Gsta2	3.00E-03	5.00E-03	0.84
Prdx3	2.75E-12	1.20E-11	0.83
Cbr3	5.00E-03	2.19E-12	0.97
Gstm6	2.13E-07	7.24E-07	0.81
Gstm2	4.79E-16	6.07E-15	0.75
Srxn1	1.61E-17	2.05E-19	0.62
Ccl2	8.00E-04	3.90E-06	0.19
Cd14	9.00E-04	3.20E-06	0.17
Cd68	4.00E-03	4.50E-06	0.07
Il10rb	3.60E-09	4.50E-09	0.96
Gldc	1.01E-17	4.17E-15	0.46
Sdsl	5.42E-14	2.18E-11	0.33
Hal	4.88E-07	1.31E-07	0.8

Got1	3.41E-23	2.21E-22	0.89

Table S4: P values for heatmap from Figure 6C

Gene name	HB vs NT	Tumouroid vs NT	Tumouroid vs Tum
Nkd1	0.00E+00	0.00E+00	0.82
Axin2	0.00E+00	0.00E+00	0.6
SP5	1.20E-93	1.00E-91	0.82
Notum	3.51E-83	4.06E-82	0.89
Znrf3	1.24E-53	7.09E-52	0.78
Rnf43	6.41E-26	3.87E-28	0.63
Tnfrsf19	2.86E-72	3.04E-71	0.89
Lef1	5.71E-47	4.15E-46	0.88
Tead1	4.00E-04	4.00E-04	0.99
Tead2	2.00E-39	6.38E-37	0.62
Tead4	6.73E-16	1.21E-17	0.55
Ankrd12	4.00E-04	4.32E-05	0.56
Nuak1	1.54E-08	1.79E-08	0.97
Tgfb2	1.32E-38	5.38E-40	0.79
Adam12	5.99E-24	1.77E-27	0.4
Adam19	2.14E-08	2.58E-10	0.45
Col1a2	2.87E-06	4.05E-07	0.69
Col3a1	5.00E-04	1.00E-04	0.66
Sox4	6.71E-47	3.72E-49	0.71
Sfrp1	2.66E-27	5.21E-26	0.75
Rspo3	1.00E-03	1.00E-03	0.98
Dkk1	8.41E-14	6.64E-14	0.89
Vim	5.47E-34	5.20E-36	0.7
Mmp2	7.99E-19	5.21E-20	0.76
Zeb1	3.42E-18	1.36E-18	0.91
Zeb2	1.42E-08	6.80E-11	0.38
Cdh1	1.00E-03	7.00E-04	0.77
Cdh2	2.00E-03	3.00E-03	0.96
Mcam	3.40E-06	2.85E-05	0.64
CD34	6.95E-15	1.78E-15	0.85
s	1.11E-07	3.14E-07	0.84
Mki67	3.69E-10	8.25E-10	0.89
E2f1	2.71E-05	3.13E-05	0.97
CD163	3.44E-08	7.98E-11	0.55
Il10rB	5.00E-03	2.00E-03	0.8
Ccr2	9.00E-03	2.00E-02	0.77
Cxcl10	7.00E-03	1.00E-02	0.77
Cxcl11	5.64E-06	5.56E-06	0.99
