# Supplementary material for: Novel 3D models for β-catenin-driven liver cancers
Source: JHEP Rep. 2026 Sep 18;8(12):102040. doi: 10.1016/j.jhepr.2026.102040 (PMC13755961; doi:10.1016/j.jhepr.2026.102040)
Supplement: Supplementary file 3 [file mmc5.pdf]

JHEP Reports

Novel 3D models for β-catenin-driven liver cancers

Authors

Vanessa Bou Malham, Fanny Léandre, Akila Hamimi, …, Angélique Gougelet, Sabine Colnot, Christèle Desbois-Mouthon

Correspondence

christele.desbois-mouthon@inserm.fr (C. Desbois-Mouthon), vanessa.bou-malham@inserm.fr (V. Bou Malham).

Graphical abstract

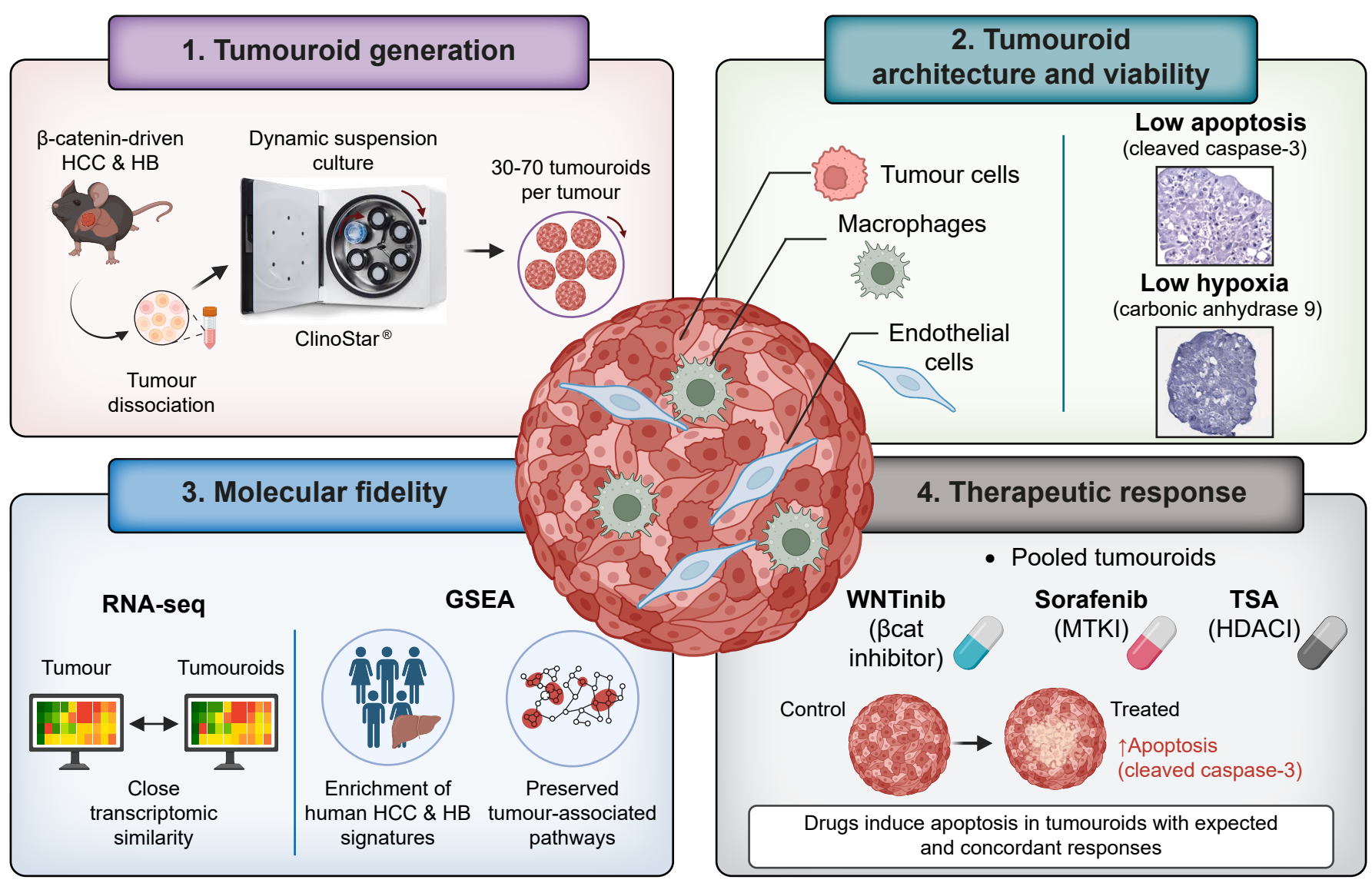

Tumouroids faithfully recapitulate tumour characteristics and tumour microenvironment providing a robust preclinal model for therapeutic testing

Highlights:

- Easy and rapid generation of mouse organoids and tumouroids from β-catenin-activated livers and tumours using a rotating bioreactor culture system.
- 3D models preserve the histological architecture, cellular diversity, and gene expression profiles of the corresponding native liver tissue.
- 3D models exhibit expected responses to several drugs, including the β-catenin inhibitor WNTinib.
- These robust 3D models provide a valuable platform for drug screening while reducing reliance on animal experimentation.

Impact and implications:

This study addresses the need for representative *in vitro* models to investigate β-catenin-driven liver cancers. The tumouroids developed here provide robust and reproducible 3D models that closely recapitulate the histological features, cellular diversity, and gene expression profiles of native liver tumours. These models offer a valuable platform for studying tumour biology, evaluating therapeutic candidates, and supporting the development of personalised treatment strategies. They may also contribute to reducing the use of animals in preclinical research.

https://doi.org/10.1016/j.jhepr.2026.102040

© 2026 The Author(s). Published by Elsevier B.V. on behalf of European Association for the Study of the Liver (EASL). This is an open access article under the CC BY license (http://creativecommons.org/licenses/by/4.0/).

JHEP|Reports

Novel 3D models for β-catenin-driven liver cancers

Vanessa Bou Malham[1,2,3,4,*], Fanny Léandre[1,2,3], Akila Hamimi[1,2,3], Isabelle Lagoutte[5], Sandrine Bouchet[1,2,3,6], Alexandre Archambault-Marsan[7,8], Jérémy Augustin[7,9], Angélique Gougelet[1,2,3], Sabine Colnot[1,2,3], Christèle Desbois-Mouthon[1,2,3,*]

JHEP Reports **2026**. vol. 8 | 1–14

Check for updates

Background & Aims: Constitutive activation of β-catenin is a key feature in the pathogenesis of hepatocellular carcinoma (HCC) and hepatoblastoma (HB). Activating alterations in the *CTNNB1* gene and, to a lesser extent, inactivating alterations in the *APC* gene are observed in 30-40% of HCC cases and 80-90% of HB cases. For both tumours, therapeutic options remain suboptimal. Therefore, relevant experimental models are needed to improve our understanding and to test new therapeutic approaches.

Methods: Liver organoids (n = 17 mice) and tumouroids (n = 14 mice) were established from $APC^{\Delta hep}$ and $\beta cat^{\Delta ex3}$ mouse models, which are clinically relevant models of β-catenin-activated HCC and mesenchymal HB. We developed a new methodological approach based on dynamic suspension culture in a rotating bioreactor. Morphological and molecular characteristics, as well as drug responses, were evaluated by histology, immunohistochemistry, immunofluorescence, transcriptomics and reverse-transcription quantitative PCR.

Results: This easy-to-implement methodology allows for the rapid production of large numbers of uniform organoids and tumouroids (30-75 per tumour) without detectable cell death and hypoxia in their core. The robustness of the approach is also illustrated by the preservation of histological architecture, cellular diversity (tumour cells, macrophages, and endothelial cells) and gene expression in organoids and tumouroids compared with native liver tissue. Gene set enrichment analysis showed significant enrichment of 10 human HCC/HB tumour signatures in murine tumouroids. Organoids and tumouroids showed the expected responses to specific inhibitors, including the β-catenin antagonist WNTinib.

Conclusions: These organoids and tumouroids represent robust *in vitro* models of β-catenin-driven HCC and mesenchymal HB. They provide a scalable platform for drug screening and may support the development of "à la carte" therapies urgently needed for these indications.

© 2026 The Author(s). Published by Elsevier B.V. on behalf of European Association for the Study of the Liver (EASL). This is an open access article under the CC BY license (http://creativecommons.org/licenses/by/4.0/).

Introduction

Oncogenic activation of β-catenin is a key driver of primary liver cancers in both adults and children, namely hepatocellular carcinoma (HCC) and hepatoblastoma (HB), respectively. HCC is the most common primary liver malignancy, with more than 900,000 new cases diagnosed worldwide annually, and represents the third leading cause of cancer-related death, accounting for more than 830,000 deaths in 2020.[1] HB is the most common primary liver cancer in children, typically diagnosed before five years of age, with an estimated incidence of 1-1.5 cases per million children and a marked increase over recent decades.[2]

Recurrent genetic alterations affecting *CTNNB1*, encoding β-catenin, are frequently observed in primary liver cancers. Gain-of-function mutations in *CTNNB1* occur in 30-40% of HCC, whereas gain-of-function deletions are detected in approximately 80% of HB. Loss-of-function deletions in *APC,* a tumour-suppressor gene encoding a key negative regulator of the β-catenin pathway, have also been reported in approximately 3% of HCC and 10% of HB cases.[3,4] These alterations induce β-catenin stabilisation and nuclear accumulation, enabling β-catenin to interact with the transcription factor TCF-4 and activate transcriptional programmes involved in tumour initiation and progression. β-catenin pathway-altered HCC represents a relatively homogenous subgroup of well-differentiated tumours with low proliferative activity. Although associated with favourable outcomes when detected early and treated surgically,[3,4] no specific treatment is currently available for advanced *CTNNB1*-mutated HCC, and oncogenic β-catenin activation has been linked to resistance to immune checkpoint inhibitor therapies.[5–8] HB exhibits marked phenotypic diversity, with three major histological subtypes spanning a spectrum of differentiation states[9] and reflecting distinct transcriptomic profiles.[10–13] Current treatment relies on cisplatin-based chemotherapy combined with surgical resection, resulting in 5-year survival rates exceeding 80%.[14] However, unresectable, chemoresistant or recurrent tumours are associated with poor outcomes, and chemotherapy-associated toxicity remains a major clinical concern.

For many years, our team has generated and characterised mouse models of β-catenin-driven liver carcinogenesis. Using

* Corresponding authors. Address: INSERM, Université Paris Cité, Sorbonne Université, Centre de Recherche des Cordeliers, F-75006 Paris, France.
E-mail addresses: christele.desbois-mouthon@inserm.fr (C. Desbois-Mouthon), vanessa.bou-malham@inserm.fr (V. Bou Malham).
https://doi.org/10.1016/j.jhepr.2026.102040

Cre-lox recombination and, more recently, CRISPR/Cas9-based approaches, we demonstrated that oncogenic activation of β-catenin signalling in hepatocytes, through loss of *Apc* expression ($APC^{\Delta hep}$ model) or stabilising mutations in *Ctnnb1* ($\beta cat^{\Delta ex3}$ model), is sufficient to induce transformation and tumour initiation.[15,16] $APC^{\Delta hep}$ and $\beta cat^{\Delta ex3}$ mice develop HCC and HB-like tumours with comparable incidence. These tumours closely recapitulate the histological and transcriptional features of human β-catenin-mutated HCC and mesenchymal HB, respectively.[16] These models have proven valuable for elucidating β-catenin-driven hepatocyte transformation, and for evaluating diagnostic and therapeutic strategies.[17–19]

3D *in vitro* culture systems have emerged as valuable approaches to bridge the gap between conventional 2D cultures and *in vivo* models, particularly in cancer research.[20–22] Tumour-derived organoids (tumouroids) better reproduce native tumour architecture and functions by preserving cell-cell and cell-extracellular matrix interactions, tumour heterogeneity, and microenvironmental complexity. Several methods have been developed, ranging from simple ultra-low attachment (ULA) plate cultures to more sophisticated scaffold-based systems, organ-on-chip platforms and bioreactors, each presenting specific advantages and limitations.[23] Human liver tumouroid generation predominantly relies on extracellular matrix embedding,[24–29] whereas ULA plate-based approaches remain less frequently reported.[30] Moreover, limited data are available for human tumouroids derived from β-catenin-mutated HCC and mesenchymal HB. Previous studies indicate that human HCC-derived tumouroids are more readily established from moderately to poorly differentiated tumours, limiting representation of β-catenin-mutated HCC subtypes.[24,30]

In the present study, we investigated whether tumouroids generated from our clinically relevant mouse models could address this gap in available tumouroid models for β-catenin-mutated HCC and mesenchymal HB. We used an original dynamic suspension culture system based on a rotating bioreactor (ClinoStar® system, Celvivo). Previous studies have demonstrated that this system supports the generation of HepG2/C3A-derived 3D cultures suitable for liver pathophysiology studies.[31–34] After optimising culture conditions for mouse hepatocytes with activated β-catenin signalling, we demonstrate that this technology enables the maintenance of murine HCC and HB-like tumouroids while preserving phenotypic and transcriptional features of their tumours of origin. These novel 3D preclinical models provide valuable tools for basic and translational research on β-catenin-driven liver cancers, as demonstrated by their preserved responses to pharmacological treatments.

Materials and methods

Mouse models and procedures

For the generation of $APC^{\Delta hep}$ liver organoids, *Apc*$^{fl/fl}$;TTR-CreERT2 mice (2-3-month-old, males) were injected intraperitoneally with 2 mg tamoxifen (MP Biomedicals) diluted in corn oil.[15] For the generation of $APC^{\Delta hep}$ liver tumouroids, *Apc*$^{fl/fl}$ mice (2-3-month-old males) were injected with an adenovirus encoding Cre recombinase (AdCre, 0.5 x 10^9 particles), resulting in APC loss in a fraction of hepatocytes (30-40%), subsequent β-catenin activation, and the development of liver tumours within 4-6 months.[15] For the generation of $\beta cat^{\Delta ex3}$ liver tumouroids, C57BL/6J mice were injected with two adeno-associated virus serotype 8 (AAV8) vectors encoding saCas9 and sgRNA targeting *Ctnnb1* to induce the specific deletion of *Ctnnb1* exon 3 in a subset of hepatocytes as previously reported.[16] In both models, tumour development was followed by 2D ultrasound under isoflurane anesthesia, as previously described.[18,19] The maximum cumulative tumour diameter permitted by the ethics committees was 12 mm and was not exceeded. For terminal tissue and blood collection, mice were anesthetised with ketamine (80 mg/kg) and xylazine (5 mg/kg). Mice were housed under a 12/12-h light/dark cycle at 22–24°C with *ad libitum* access to food (Safe A03, SAFE® Complete Care Competence) and water. All animal experiments were conducted in accordance with French government regulations and were approved by two ethics committees (Charles Darwin, Sorbonne Université, APAFIS#16420, APAFIS#45107 and CEEA34, Université de Paris, APAFIS#14472).

ClinoReactor® preparation

One day before use for organoid or tumouroid culture, the humidification beads inside the ClinoReactors (CelVivo) were hydrated with 20 ml sterile ultra-pure water (CelVivo). The ClinoReactors were then thoroughly washed with DPBS for 15 min at a rotation speed of 15 rpm before cell seeding, as previously described.[32]

2D culture of hepatocytes and generation of hepatic organoids from $APC^{\Delta hep}$ mice

$APC^{\Delta hep}$ mouse livers were perfused with collagenase six days after tamoxifen administration, and hepatocytes and non-parenchymal cells (NPCs) were isolated and counted as previously described.[35] Hepatocyte viability was systematically assessed by trypan blue exclusion and was consistently greater than 90%. Detailed procedures for 2D culture are provided in the supplementary methods. For culture in 96-well ULA plates (Greiner), a mixture of 2,000 hepatocytes and 1,000 NPCs was seeded into each well in 100 µl of culture medium. For dynamic culture in the ClinoStar, 500,000 hepatocytes and 250,000 NPCs were seeded into a ClinoReactor rotating at 45 rpm. For culture in Matrigel™ (Corning), a mixture of 50,000 hepatocytes and 25,000 NPCs was suspended in 40 µl of Matrigel and seeded as a dome into each well of a 24-well plate. After polymerisation at 37°C, 500 µl of culture medium was added to each well. After four days, the medium in the 3D cultures was replaced with the same medium without FBS. Thereafter, the medium was renewed every 3-4 days. The cell seeding densities used for ULA and Matrigel cultures were selected based on the conditions most frequently reported in the literature. In contrast, the seeding density for dynamic cultures was optimised experimentally. The maximum number of cells compatible with the ClinoReactors capacity was used to minimise organoid aggregation and fusion during culture. All cultures were maintained at 37°C in a humidified atmosphere containing 5% CO_2. Bright-field images of organoids were acquired using a MATEO TL digital transmitted-light microscope (Leica Microsystems).

Generation of hepatic tumouroids from APC$^{\Delta hep}$ and βcat$^{\Delta ex3}$ mice

Tumours were excised and immediately placed on ice in basal DMEM F-12 medium (Gibco) until processing. The tumours were then gently minced into small pieces using fine scissors, transferred to a 50 ml centrifuge tube containing 10 ml of digestion solution (0.75 mg/ml collagenase (Sigma-Aldrich) and 0.15 U dispase (STEMCELL™ technologies) in basal DMEM F-12 medium), and incubated at 37°C for 20 min under gentle agitation. Three to four rounds of enzymatic digestion were performed, depending on tumour size and type. After digestion, the cell suspension was filtered through a 100-μm cell strainer (Falcon®) to remove aggregates and centrifuged at low speed (50 x g for 5 min at room temperature) to separate hepatocytes (pellet) from NPCs (supernatant). The NPC fraction was then centrifuged at 300 x g for 5 min. The hepatocytes and NPC fractions were then combined, resuspended in 10 ml of HepatiCult™ Organoid Growth Medium (Mouse) (STEMCELL™ technologies), and seeded into a ClinoReactor. The cell suspension was cultured in the ClinoStar system at 45 rpm for up to 16 days, with the medium replaced every 3-4 days. Table S1 summarises the characteristics of the tumour samples used for transcriptomic analysis, including tumour type, tumour size at sacrifice, and the total number of tumouroids generated after tissue fragments had been reserved for RNA extraction and immunohistochemistry (IHC).

Organoid and tumouroid treatment with inhibitors

After 8 days of culture in the ClinoStar, organoids and tumouroids were transferred into ClinoReactors containing either vehicle (control condition) or WNTinib (1 μM for organoids and 5 μM for tumouroids; MedChemExpress), sorafenib (5 μM, Selleck Chemicals), or trichostatin A (TSA; 0.5 μM; Selleck Chemicals). Treatments were maintained for 5 days and renewed every 2 days.

Total RNA isolation, reverse transcription and quantitative real-time PCR

Total RNA was extracted from liver tissues, organoids and tumouroids using TRIzol™ reagent (Thermo Fisher Scientific) according to the manufacturer's instructions. cDNA was synthesised using Maxima reverse transcriptase (Thermo Fisher Scientific). qPCR was performed with specific primers using either BlasTaq™ 2X PCR master mix (abm®) or TaqMan™ gene expression assays (Applied Biosystems). Target gene expression was normalised to that of *B2m,* the most stable reference gene under our experimental conditions*,* and expressed as relative expression using the $2^{-\Delta\Delta Ct}$ method. For selected experiments, a pool of liver tissue cDNA was used as a calibrator.

Histology, IHC, TUNEL assay and multiplexed immunofluorescence

H&E staining, IHC and TUNEL were performed on formalin-fixed, paraffin-embedded liver tissue sections obtained as previously described.[19] Detailed procedures for IHC, TUNEL and multiplexed immunofluorescence are provided in the supplementary methods. Whole-slide images of IHC sections were acquired using an AxioScan Z1 scanner (Zeiss), whereas fluorescent and TUNEL images were acquired using an Eclipse E800 microscope (Nikon) and Axio Observer Z1 microscope (Zeiss). The number of stained cells per field was quantified using QuPath software (v0.7.0).[36] The surface area and nuclear density of organoids and tumouroids were quantified using Fiji software.

RNA sequencing analysis

RNA sequencing was performed on total RNA extracted from APC$^{\Delta hep}$ non-tumour liver tissues (n = 8), tumours (n = 5 for HCC; n = 4 for HB), and pools of tumouroids established from each tumour. Of note, two HCCs (tumours 4.B and 4.G) developed in the same mouse liver (Table S1). Detailed RNA sequencing data processing and statistical analysis methods are provided in the supplementary methods. Processed datasets, including lists of up- and down-regulated genes from the different comparisons, are provided in Dataset S1. Heatmaps were generated using the web-based tool Morpheus (https://software.broadinstitute.org/morpheus), and KEGG pathway enrichment analyses were performed using ShinyGO 0.85.1. For gene set enrichment analysis (GSEA), previously published human HCC and HB gene signatures were converted into mouse gene sets using orthology mappings from the MSigDB (Molecular Signatures Database). The resulting mouse ortholog gene sets were used as custom signatures for enrichment analysis (Table S2). Details for GSEA analysis are provided in the supplementary methods.

Statistical analysis

Statistical analysis was performed using GraphPad Prism (v10.6.1). Differences between two groups were assessed using the Mann-Whitney test. For comparisons involving more than two groups, the Kruskal-Wallis test was performed. A p value <0.05 was considered statistically significant.

Detailed information on antibodies, reagents, PCR primer pairs, TaqMan™ gene expression assays, animal models, and experimental resources is provided in the CTAT table.

Results

The ClinoStar system enables the generation of liver organoids from APC$^{\Delta hep}$ mice

For many years, 2D culture of human and murine hepatocytes on collagen matrix has been considered the gold standard approach. However, rapid cell dedifferentiation and cell mortality associated with this culture system have limited its relevance for maintaining hepatocyte identity.[37] Accordingly, we investigated whether 2D culture altered the phenotype of APC^{KO} hepatocytes isolated from *Apc*$^{fl/fl}$;TTR-CreERT2 mice injected with 2 mg tamoxifen (Fig. S1A). This treatment induces *Apc* deletion in approximately 90% of hepatocytes (Fig. S1B), resulting in the activation of β-catenin-dependent pathways throughout the liver parenchyma. Freshly isolated APC^{KO} hepatocytes overexpressed major direct β-catenin target genes, including *Glul* (encoding glutamine synthetase (GS)), *Axin2*, *Lgr5*, and *Slc13a3* compared with wild-type (WT) hepatocytes isolated from tamoxifen-treated *Apc*$^{fl/fl}$ mice (Fig. S1C). After 24 h of 2D culture, their expression remained significantly elevated in *APC*^{KO} hepatocytes, although the magnitude of induction was substantially lower than in freshly isolated

APC^{KO} hepatocytes (Fig. S1C). These findings indicate that 2D monoculture rapidly compromises the maintenance of the β-catenin-activated hepatocyte phenotype.

We next investigated whether dynamic 3D culture could better support the β-catenin-driven phenotype in hepatocytes. We therefore assessed the rotating ClinoStar bioreactor and compared its performance with that of ULA plates and Matrigel-based culture. *APC*^{KO} hepatocytes and NPCs were combined at a 2:1 ratio and cultured either in a ClinoReactor within the ClinoStar system, on ULA plates, or embedded in Matrigel (Fig. 1A). Of note, the initial cell seeding densities varied across culture systems: 750,000 cells per ClinoReactor (75,000 cells/ml), 3,000 cells per ULA well (30,000 cells/ml), and 75,000 cells embedded in a 40 µl Matrigel dome (1.88 x

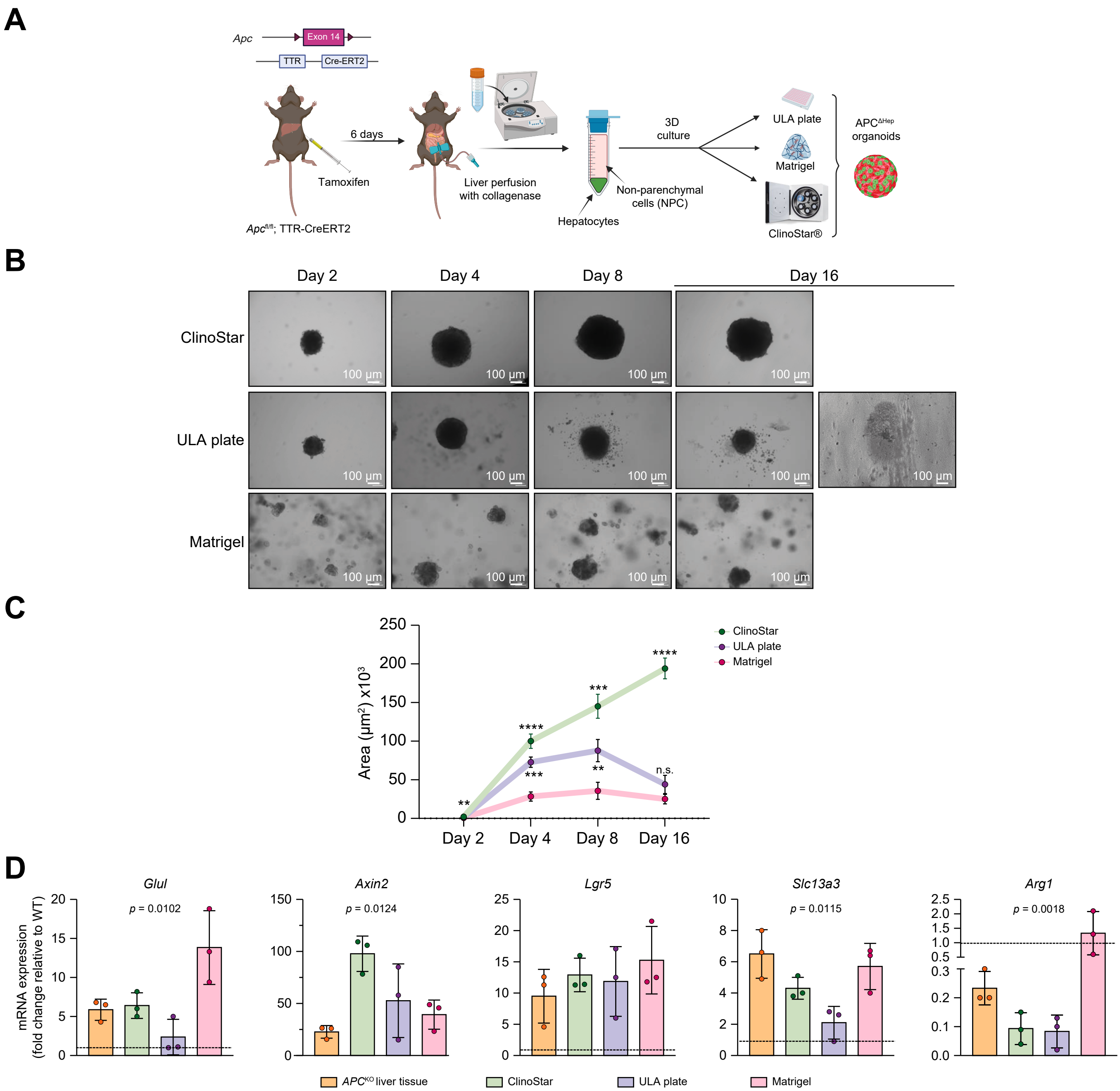

Fig. 1. The ClinoStar system supports the growth of APC$^{\Delta hep}$ liver organoids. (A) Schematic illustration of the experimental design for 3D organoid culture (created with BioRender.com). (B) Representative brightfield images of APC$^{\Delta hep}$ liver organoids cultured in ULA plates, Matrigel, or the ClinoStar system at days 2, 4, 8, and 16. (C) Quantification of projected organoid area. $^{**}p$ <0.01; $^{***}p$ <0.001; $^{****}p$ <0.0001. Differences were assessed using the Kruskal-Wallis test. (D) Relative transcript expression assessed by RT-qPCR in APC$^{\Delta hep}$ liver tissue and liver organoids compared with WT liver tissue normalised to 1; the dashed horizontal line indicates this reference level. Global differences were assessed using the Kruskal-Wallis test. Pairwise comparisons were performed using Dunn's multiple comparisons test; no pairwise comparison reached statistical significance. Values are mean ± SD (n = 3 biological replicates, one mouse per replicate). For each replicate, areas were analysed for 12-20 organoids and RNA was extracted from a pool of 20-25 organoids. RT-qPCR, reverse-transcription quantitative PCR; ULA, ultra-low attachment; WT, wild type.

10^6 cells/ml). By day 4, APC$^{\Delta Hep}$ organoids had formed under all culture conditions, demonstrating efficient 3D self-organisation of hepatocytes and NPCs (Fig. 1B,C). By day 8, organoids generated in the ClinoStar system displayed a uniform spherical morphology, and a larger projected area than those formed in ULA plates and Matrigel cultures (145 ± 15, 88 ± 14, 36 ± 14 x 10^3 μm^2, respectively). Notably, ClinoStar-derived organoids reached a larger size despite being generated from a lower initial cell density than Matrigel cultures. Organoids cultured in Matrigel also exhibited greater morphological heterogeneity. After 16 days of culture, organoids generated in the ClinoStar system retained a compact and spherical architecture. Similarly, Matrigel-derived organoids remained structurally intact despite their heterogeneous morphology. In contrast, 62% of organoids cultured in ULA plates exhibited structural loosening and reduced size, indicating impaired long-term stability. Together, these findings show that while APC$^{\Delta hep}$ organoids formed within 4 days in all culture systems tested, the ClinoStar system supported the generation of larger, more homogeneous and structurally stable organoids.

Reverse-transcription quantitative PCR analysis of canonical positive (*Glul*, *Axin2*, *Lgr5*, and *Slc13a3)* and negative (*Arg1*) β-catenin target genes demonstrated that APC$^{\Delta hep}$ organoids generated using ClinoStar recapitulated the β-catenin-dependent transcriptional signature observed in APC$^{\Delta hep}$ livers (Fig. 1D). By contrast, transcriptional responses were less consistent in organoids cultured in ULA plates or embedded in Matrigel. ULA-derived organoids showed only limited induction of *Glul* and *Slc13a3*, whereas Matrigel-cultured organoids failed to reproduce the downregulation of *Arg1* after 8 days of culture (Fig. 1D). Histology and IHC analyses were performed to further characterise APC$^{\Delta hep}$ organoids generated using the ClinoStar system at D8 (Fig. 2A). H&E staining revealed well-preserved tissue architecture in ClinoStar-derived organoids, which also exhibited reduced cell death, as evidenced by decreased cleaved caspase-3 staining (Fig. 2B,C), TUNEL positivity (Fig. 2D), and *Noxa* mRNA expression (Fig. S2A) compared with organoids generated using ULA plates. Ki67 staining revealed low proliferative activity in ClinoStar-derived organoids at D4, comparable to that observed in organoids generated using ULA plates (Fig. 2C). By D8, however, ClinoStar-derived organoids exhibited significantly higher proliferative activity than ULA-derived organoids (Fig. 2B,C), consistent with their improved viability. These findings were corroborated by reverse-transcription quantitative PCR analysis of *Mki67* mRNA expression (Fig. S2B). Furthermore, APC$^{\Delta hep}$ organoids generated using the ClinoStar system showed a trend toward higher proliferative activity than those cultured in Matrigel (Fig. S2B).

ClinoStar-derived organoids showed homogeneous GS staining together with membranous, cytoplasmic and nuclear localisation of β-catenin, consistent with sustained activation of the β-catenin pathway. Importantly, NPCs were also detected, including F4/80-positive macrophages and CD146-positive endothelial cells (Fig. 2B, C). In contrast, ULA-derived organoids exhibited weaker immunostaining for GS, β-catenin, F4/80, and CD146, accompanied by increased background staining (Fig. 2B, C). To further assess hypoxic stress, hypoxia was evaluated by carbonic anhydrase 9 (CA9) immunostaining and by measuring the expression of the HIF1-responsive genes *Epo* and *Vegfa*. ClinoStar-derived organoids exhibited reduced CA9 staining and lower *Epo* and *Vegfa* expression than ULA-derived organoids (Fig. S2C and D). Collectively, these data demonstrate that the ClinoStar system offers favourable conditions for the generation of β-catenin-activated liver organoids by preserving tissue architecture, cellular viability, and cellular heterogeneity while limiting hypoxic stress.

The ClinoStar system enables the generation of tumouroids from APC$^{\Delta hep}$ and βcat$^{\Delta ex3}$ mouse liver tumours

These findings prompted us to investigate whether the ClinoStar system could also be used to generate β-catenin-activated tumouroids. To this end, *Apc*$^{fl/fl}$ mice were injected with a low dose of an adenovirus encoding Cre recombinase (AdCre) and monitored by ultrasound imaging for tumour development over several months. Once tumours were detected, they were harvested, mechanically and enzymatically dissociated, and seeded into bioreactors (Fig. 3A). Two types of tumours were obtained from these mice: well-differentiated APC$^{\Delta hep}$ HCC and undifferentiated APC$^{\Delta hep}$ HB-like tumours.

After 8 days of culture in the ClinoStar, tumour cells and NPCs isolated from both HCC and HB-like tumours formed 3D structures, yielding approximately 30-75 tumouroids per culture with diameters ranging from 150 to 200 μm. These tumouroids reproduced key structural and cellular features of their corresponding tumour tissue. APC$^{\Delta hep}$ HCC and HB-like tumouroids retained activation of the β-catenin pathway, as demonstrated by cytoplasmic GS staining and nuclear β-catenin staining (Figs 3B,C and 4A,B; Table 1). HB-like tumouroids also exhibited vimentin expression, consistent with their mesenchymal phenotype (Fig. 4A,B). After 16 days of culture, HCC-derived tumouroids showed no detectable CA9 immunostaining within their core, whereas CA9-positive areas were observed in some HB-like tumouroids (Fig. S3A). This observation is consistent with the gene expression data showing higher *Vegfa* expression in both primary HB-like mouse tumours and the corresponding tumouroids than in their HCC counterparts (Fig. S3B), suggesting that the limited hypoxic response detected in HB-like tumouroids may primarily reflect the intrinsic characteristics of the tumour cells rather than the culture conditions. Overall, both tumouroid models exhibited little evidence of marked hypoxic stress under the conditions used. Consistent with this finding, cleaved caspase-3 staining was absent from the core of tumouroids, regardless of their tumour of origin, whereas Ki67-positive hepatocytes were distributed throughout the structures (Figs. 3B and 4A). As previously observed in the preneoplastic organoids (Fig. 2B,C), F4/80 and CD146 staining confirmed the presence of macrophages and endothelial cells in tumouroids, respectively (Figs 3B and 4A). In addition to conventional chromogenic IHC, HCC tumouroids were analysed using a high-throughput automated multiplex immunofluorescence platform based on high-pressure microfluidic circuits. As shown in Fig. S4, markers specific for the different cellular populations were readily detected within APC$^{\Delta hep}$ HCC tumouroids.

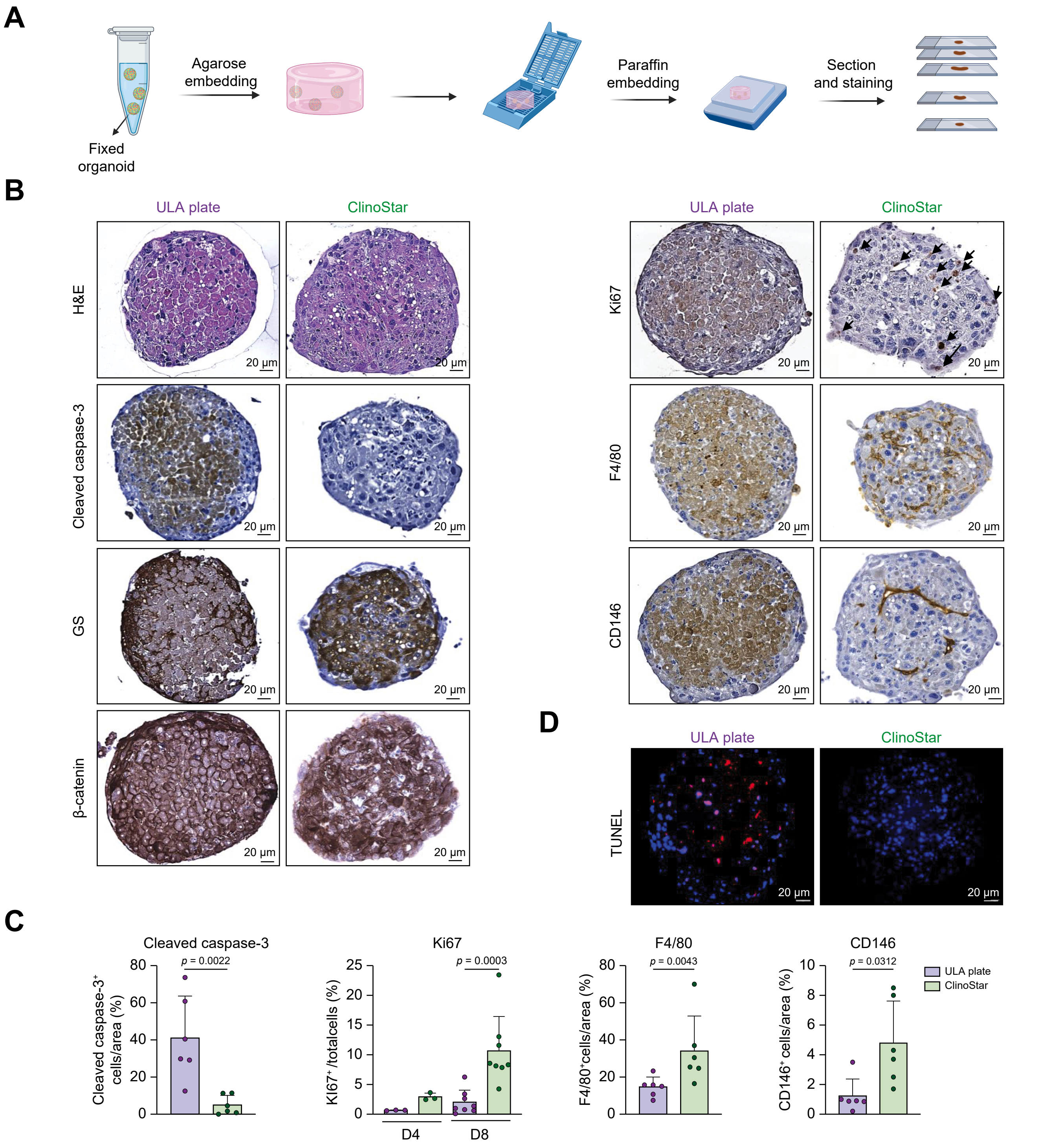

Fig. 2. The ClinoStar system promotes an organised tissue architecture in APC$^{\Delta hep}$ liver organoids. (A) Schematic illustration of the spheroid inclusion protocol (created with BioRender.com). (B) Representative H&E staining and immunostaining in APC$^{\Delta hep}$ organoids obtained after 8 days in ULA plates or in the ClinoStar. Black arrows indicate Ki67-positive nuclei. (C) Quantification of immunostaining. (D) Representative images of apoptotic cells detected by TUNEL staining (red), with nuclei counterstained with DAPI (blue), in organoids cultured for 8 days in ULA plates or ClinoStar. Values are mean ± SD (n = 6 biological replicates, one mouse per replicate; 8-14 organoids per replicate). Differences were assessed using the Mann-Whitney test. ULA, ultra-low attachment.

We further validated these data using βcat$^{\Delta ex3}$ HCC, which shares a similar transcriptomic profile with APC$^{\Delta hep}$ HCC.[16] Tumouroids derived from βcat$^{\Delta ex3}$ HCC retained the histological architecture of their tumour of origin (Fig. S5). Altogether, these data indicate that dynamic culture in the ClinoStar system enables the generation of tumouroids that

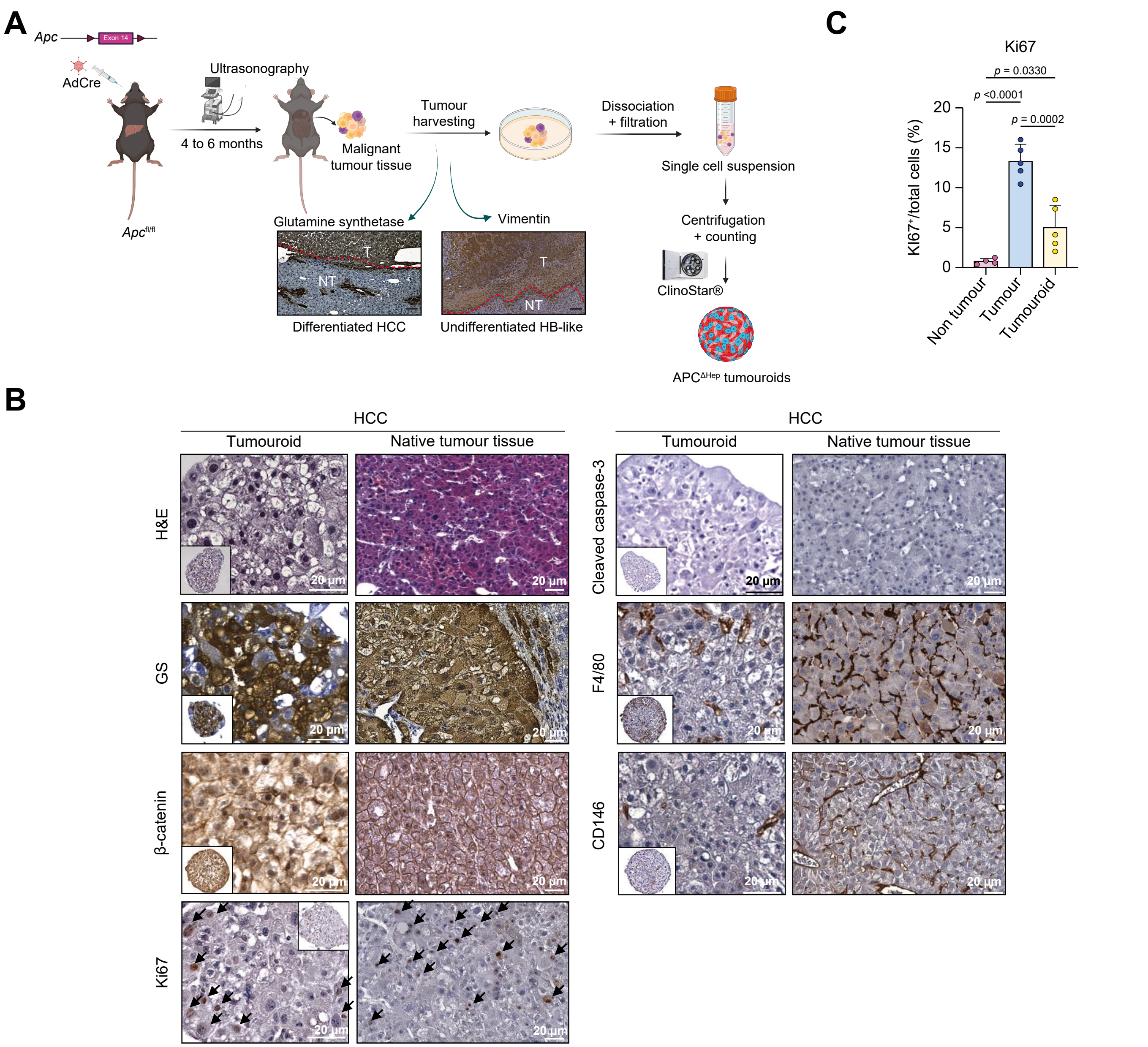

Fig. 3. Culture in the ClinoStar generates and preserves APC$^{\Delta hep}$ HCC tumouroids. (A) Schematic illustration of the experimental design for 3D tumouroid culture in the ClinoStar system (created with BioRender.com). (B) Representative H&E staining and immunostaining in tumouroids generated after 8 days of culture in the ClinoStar, compared with corresponding native HCC tissue. Black arrows indicate Ki-67 positive nuclei. (C) Quantification of Ki67-positive cells (n = 4-5 biological replicates, one mouse per replicate). Differences were assessed using the Kruskal-Wallis test. For each biological replicate, 7-13 tumouroids were analysed. HCC, hepatocellular carcinoma.

maintain key histological and cellular features of murine β-catenin-activated HCC and HB-like tumours while preserving intra-tumour cellular heterogeneity.

APC$^{\Delta hep}$ tumouroids cultured in the ClinoStar system retain the transcriptomic profile of their tumour of origin

To further characterise APC$^{\Delta hep}$ HCC (n = 5) and HB-like tumouroids (n = 4), RNA sequencing was performed, and their transcriptomic profiles were compared with those of the corresponding native tumours and adjacent non-tumour tissues. Principal component analysis showed that each tumouroid clustered closely with its tumour of origin, whereas non-tumour tissues formed a distinct cluster (Fig. 5A for HCC and Fig. 6A for HB). One sample pair (HCC#1/tumouroid#1) appeared as an outlier. Notably, HCC#1 was among the largest of the tumours analysed, and exhibited haemorrhagic and necrotic areas (Table S1). Differential gene expression analysis using MA plots further demonstrated the high degree of similarity between tumouroids and their corresponding tumours (Fig. 5B for HCC and Fig. 6B for HB). In HCC-derived tumouroids, only two genes (*Clec4d* and *Gm10273*) were differentially expressed compared with the corresponding primary tumours (Fig. 5B). Similarly, HB-like tumouroids showed a highly conserved transcriptomic profile, with only *Fam177a2* and *2810047C21Rik* genes significantly

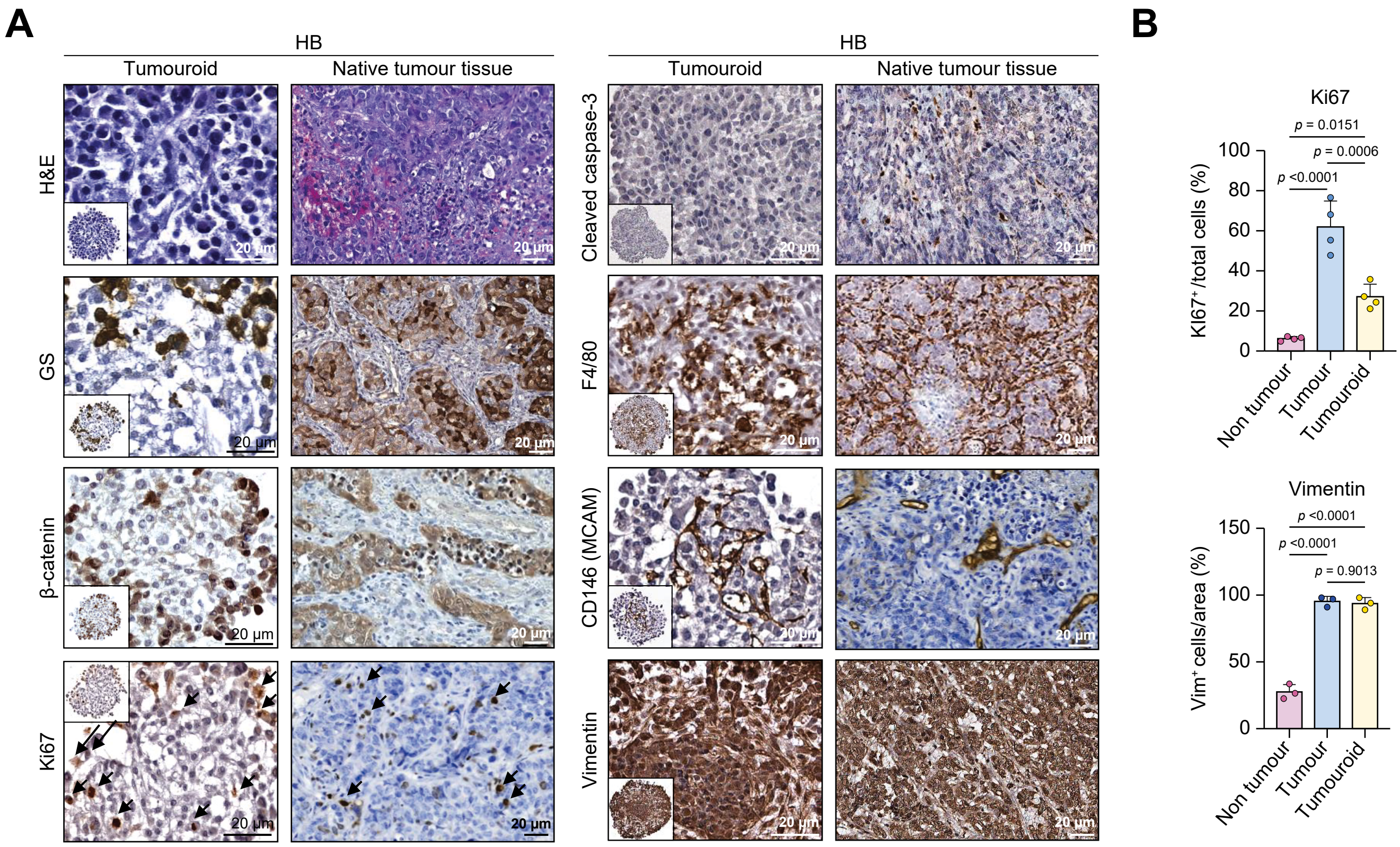

Fig. 4. Culture in the ClinoStar generates and preserves APC$^{\Delta hep}$ HB-like tumouroids. (A) Representative H&E staining and immunostaining in tumouroids generated after 8 days of culture in the ClinoStar, compared with corresponding native HB-like tissue. Black arrows indicate Ki67-positive nuclei. (B) Quantification of Ki67- and vimentin-positive cells (n = 3-4 biological replicates, one mouse per replicate). For each biological replicate, 6-10 tumouroids were analysed. Differences were assessed using the Kruskal-Wallis test. HB, hepatoblastoma.

upregulated compared with the tumour of origin (Fig. 6B). We next examined the expression of gene signatures characteristic of each tumouroid type relative to the corresponding non-tumour liver tissue (Figs. 5C, 6C, S6, and S7; Tables S3 and S4; samples HCC#1/tumouroid#1 were excluded from this analysis). Consistent with previous reports in APC$^{\Delta hep}$ HCC,[16,18,19,38,39] HCC-derived tumouroids exhibited high expression of *Ctnnb1* target genes, including both canonical targets and adult liver-specific targets involved in glutamine, bile acid, and xenobiotic metabolism. They also displayed increased expression of genes associated with inflammation and redox signalling. Conversely, genes involved in amino acid catabolism, known to be repressed by β-catenin in mature hepatocytes, were expressed at low levels (Fig. 5C and S6; Table S3). Similarly, HB-like tumouroids recapitulated APC$^{\Delta hep}$ HB-like tumours,[16] including high expression of canonical *Ctnnb1* target genes, *Apc*$^{\Delta hep}$ embryonic liver genes, mesenchymal markers and YAP pathway-associated genes (Fig. 6C and S7; Table S4). Transcripts encoding vascular markers, including *Mcam*/Cd146 were significantly upregulated in both APC$^{\Delta hep}$ HCC and HB-like tumouroids, consistent with the IHC detection of endothelial cells. KEGG pathway enrichment analysis was subsequently performed by comparing tumouroids with their corresponding non-tumour tissues (Fig. S8A–D). In HCC-derived tumouroids, the most enriched pathways were associated with cell cycle progression, xenobiotic metabolism and glutathione metabolism (Fig. S8A), whereas pathways related to amino acid metabolism were among the most downregulated (Fig. S8B). In HB-like tumouroids, pathways involved in mitosis and cell division showed the highest enrichment scores (Fig. S8C), while several lipid metabolism pathways were significantly downregulated (Fig. S8D). These pathway-level alterations closely mirrored the biological characteristics of the corresponding native tumours, namely a predominantly metabolic phenotype in HCC and a highly proliferative phenotype in HB-like tumours. Collectively, these findings demonstrate that the ClinoStar-based culture system generates murine HCC and HB-like tumouroids that preserve the transcriptomic programmes, cellular complexity, and biological features of the tumours of origin.

Murine tumouroids retain features of human HCC and HB transcriptional programmes

To evaluate the relevance of the murine tumouroids generated from HCC, we investigated whether they recapitulated transcriptional programmes associated with β-catenin-mutated human HCC. GSEA was performed using four previously published human β-catenin-related HCC signatures, including the Boyault G6 Up signature,[40] the Chiang *CTNNB1* Up signature,[41] the Lachenmayer signature,[42] and the recently developed mutated β-catenin gene signature, a robust 13-gene transcriptional classifier.[43] All four signatures were enriched in murine HCC-derived tumouroids (Fig. 5D). We next examined whether tumouroids derived from murine HB recapitulate molecular features observed in human HB. To this end,

Table 1. Immunohistochemical characterisation of murine HCC and HB and their corresponding tumouroids.

Name	H-score GS	Membranous β-catenin (%)	Nuclear β-catenin (%)
**HCC**			
Tumour #2	300	100	60
Tumouroid #2	275	100	93
Tumour #4B	300	97	58
Tumouroid #4B	283	100	100
Tumour #4G	300	100	90
Tumouroid #4G	217	98	67
Tumour #7	300	100	80
Tumouroid #7	272	80	55
**HB-like**			
Tumour #5	200	55	50
Tumouroid #5	75	85	33
Tumour #9	165	75	85
Tumouroid #9	218	83	70
Tumour #14	170	95	30
Tumouroid #14	95	90	29

H-score (range 0-300) of GS and percentage of membranous and nuclear β-catenin-positive cells in murine HCC, HB, and their corresponding tumouroids. Because GS expression is substantially lower in HB than in HCC, immunostaining conditions were adjusted to optimise signal detection in each tumour type. Therefore, H-score values are not intended for direct quantitative comparison between HCC and HB. Data are presented for each individual tumour and its corresponding tumouroids (3-7 tumouroids for each tumour). GS, glutamine synthetase; HB, hepatoblastoma; HCC, hepatocellular carcinoma.

GSEA was performed using six independent human HB gene signatures (Figs 6D and S8E). The enrichment patterns observed across these datasets revealed a strong concordance between murine HB-like tumouroids and human HB transcriptional programmes. Notably, murine HB-like tumouroids were significantly enriched for the hypomethylated gene signature described by Carrillo-Reixach *et al.*,[44] while exhibiting negative enrichment of the corresponding downregulated signature. These findings indicate that they share molecular characteristics with the Epi-CB subclass of human HB, which is characterised by global DNA hypomethylation. In addition, these tumouroids displayed significant enrichment for both the embryonal and mesenchymal signatures defined by Wu *et al.*,[28] suggesting the coexistence of developmental and mesenchymal transcriptional programmes associated with more immature HB phenotypes. Together, these results demonstrate that murine HCC and HB-like tumouroids preserve key transcriptional features associated with human β-catenin-driven liver cancers, supporting their relevance as models to investigate biologically relevant aspects of these diseases.

β-catenin-driven organoids and tumouroids exhibit drug-induced antiproliferative and pro-apoptotic responses

To assess whether ClinoStar-generated organoids and tumouroids represent relevant preclinical models for pharmacological testing, we evaluated their response to three compounds with distinct mechanisms of action: WNTinib, a multikinase inhibitor previously shown to target *CTNNB1*-mutated liver cancer cells,[45,46] sorafenib, a clinically approved multikinase inhibitor targeting RAF kinases and angiogenic signalling pathways,[47] and trichostatin A (TSA), a histone deacetylase inhibitor reported to exert potent antiproliferative and pro-apoptotic effects in β-catenin-activated hepatic cancer cell lines.[48] APC$^{\Delta hep}$ organoids were cultured for 8 days before compound treatment was initiated and maintained for an additional 5 days. WNTinib induced marked morphological alterations, including disruption of organoid architecture and the appearance of cell-depleted areas within the organoid core (Fig. 7A). These structural changes were associated with reduced proliferative activity, as assessed by Ki67 immunostaining (Fig. 7A, B), and activation of an apoptotic programme, evidenced by increased cleaved caspase-3 immunolabelling (Fig. 7A,B), loss of nuclear density (Fig. 7C), increased TUNEL positivity (Fig. S10A), increased *Noxa* expression and decreased *Bcl2l1* expression (Fig. 7D). TSA elicited an even stronger pro-apoptotic response (Fig. S9A–C), whereas sorafenib produced comparatively weaker effects (Fig. S9D and E). Notably, antiproliferative and pro-apoptotic effects were not accompanied by a detectable reduction in organoid size during the treatment period (Figs 7E and S9F). βcat$^{\Delta ex3}$ HCC-derived tumouroids also responded to WNTinib treatment by undergoing cell death and reduced proliferative activity (Figs 7F-I and S10B). In an exploratory experiment using βcat$^{\Delta ex3}$ tumouroids derived from a single independent tumour, TSA treatment was associated with a more pronounced apoptotic response, with apoptotic cells representing up to 80% of the tumouroid area (Fig. S9G–I). Again, no consistent change in tumouroid size was observed under any of the treatment conditions (Figs 7J and S9J). Together, these results demonstrate that organoids and tumouroids generated in the ClinoStar are amenable to pharmacological manipulation and can discriminate between compounds inducing distinct levels of biological response, supporting their potential for drug screening applications.

Discussion

In this study, we established a matrix-free dynamic culture platform enabling the generation of murine preneoplastic organoids from livers with hepatocyte-specific β-catenin activation, together with tumouroids derived from β-catenin-activated well-differentiated HCC and poorly differentiated HB-like tumours. Histological, immunohistochemical and transcriptomic analyses demonstrated that these 3D models faithfully preserve the architecture, cellular composition and molecular features of their tumours of origin. Our findings demonstrate that dynamic suspension culture can maintain the complexity of β-catenin-driven liver tumours while enabling downstream molecular and pharmacological analyses.

The successful establishment of these models relies on the unique culture properties of the ClinoStar rotary bioreactor. By continuously counterbalancing gravitational forces, the system maintains cells in suspension under near-microgravity conditions without extracellular matrix components. This low-shear environment promotes nutrient and oxygen distribution while limiting hypoxia and cell death, contributing to the morphological homogeneity and reproducible size of liver organoids and tumouroids, consistent with recent studies using the ClinoStar system to generate brain organoids[49] and lung tumouroids.[50] Beyond these biological advantages, the matrix-free configuration simplifies tissue handling, avoids Matrigel-associated variability, facilitates recovery of intact

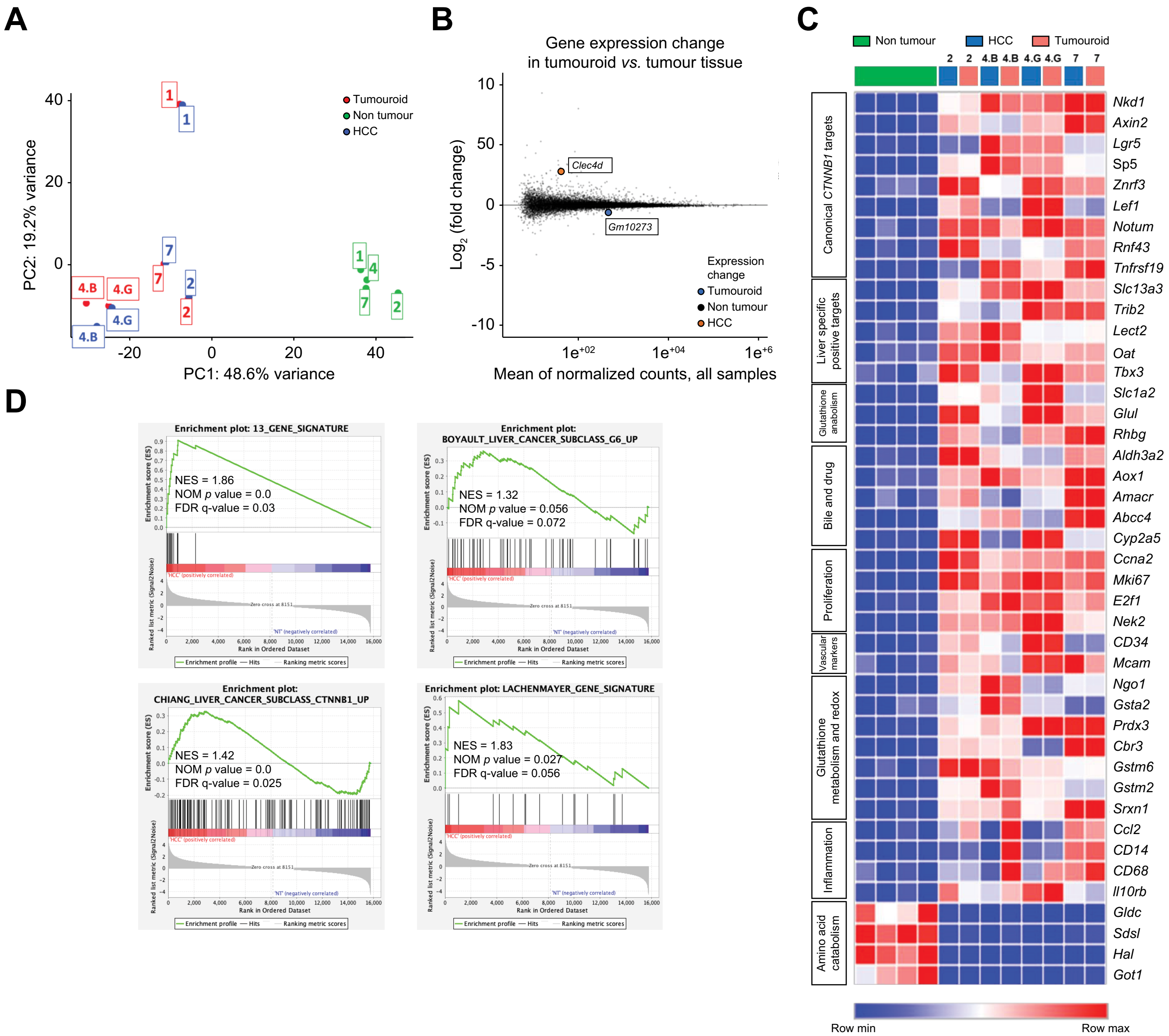

Fig. 5. Culture in the ClinoStar maintains the transcriptomic programmes of APC$^{\Delta hep}$ HCC tumouroids. (A) PCA of normalised gene expression profiles from five pooled tumouroid samples (each generated from a single HCC), five HCC, and four adjacent non-tumour liver tissues. (B) MA plot showing gene expression differences between each pool of tumouroids and its corresponding HCC. Each dot represents a gene, plotted as $\log_2$ fold change *vs*. mean normalised counts across all samples. (C) Heatmap of differentially expressed genes between four pools of tumouroids, four HCC, and four adjacent non-tumour liver tissues. Samples were clustered using unsupervised hierarchical clustering. (D) GSEA highlighting significantly enriched signatures between murine tumouroids and human HCC tissues. Enrichment results are shown for selected gene sets, with NES, NOM *p* values, and FDR q-values indicated. FDR, false discovery rate; GSEA, gene set enrichment analysis; HCC, hepatocellular carcinoma; NES, normalised enrichment score; NOM, nominal; PCA, principal component analysis.

tumouroids for molecular analyses, and enables the production of sufficient material for extensive histological characterisation. Together, these features provide a standardised and experimentally tractable alternative to conventional matrix-based culture systems.

The ClinoStar system preserves the molecular identity of individual tumours and captures inter-tumour heterogeneity rather than driving samples towards a common transcriptional profile. Principal component analysis demonstrated that tumouroids clustered with their corresponding tumour of origin, including HCC#1, a large tumour characterised by extensive haemorrhagic and necrotic regions and a distinctive transcriptional programme. These findings demonstrate preservation of tumour-specific features, an essential prerequisite for applications such as drug screening, where inter-patient variability influences therapeutic response.

A further strength of this culture system is its preservation of tumour cellular heterogeneity. Throughout at least 8 days of culture, differentiated and poorly differentiated tumour hepatocytes remained associated with macrophages and endothelial cells, indicating maintenance of key components of the tumour microenvironment *ex vivo*. This multicellular

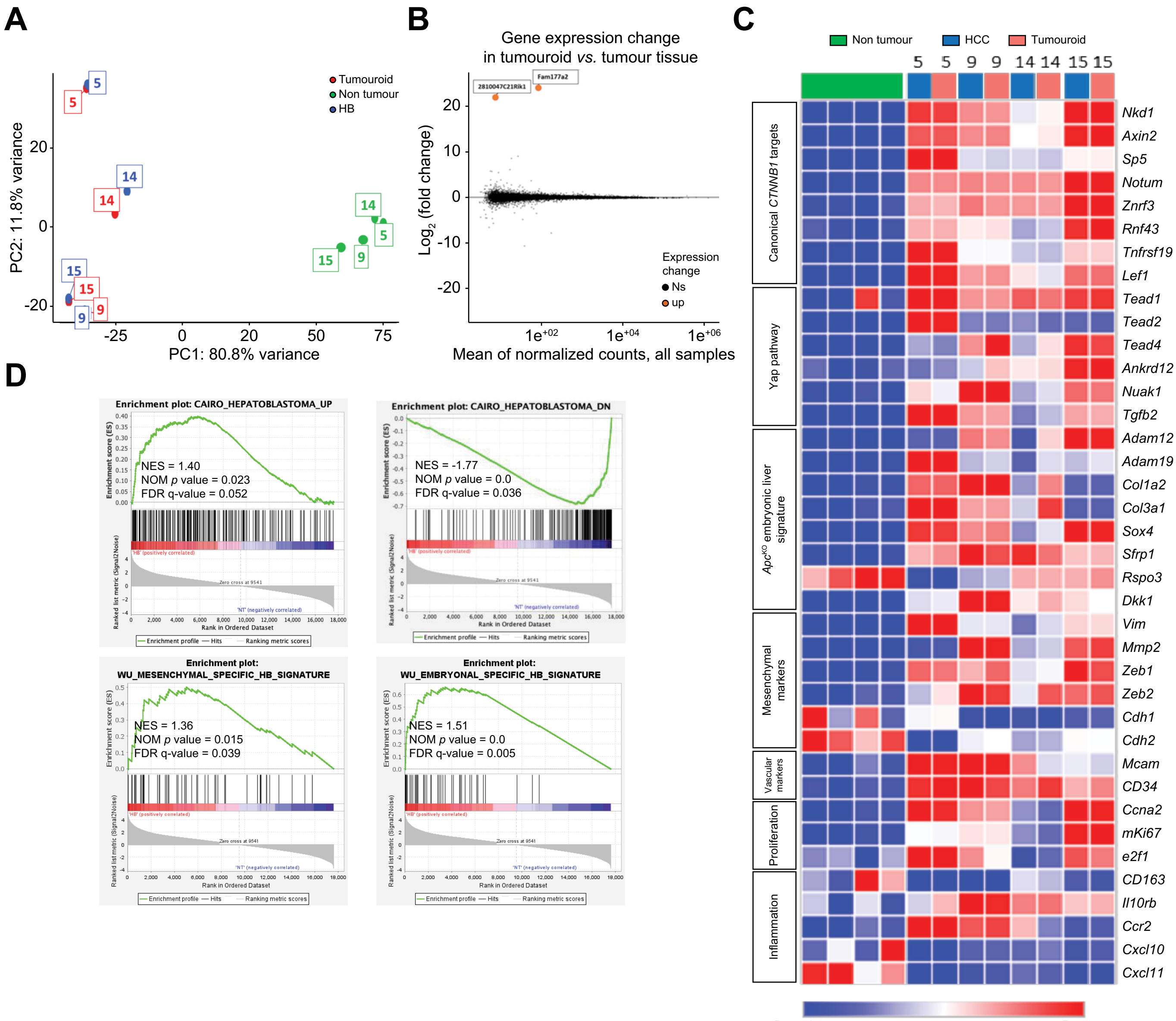

Fig. 6. Culture in the ClinoStar maintains the transcriptomic programmes of APC$^{\Delta hep}$ HB-like tumouroids. (A) PCA of normalised gene expression profiles from four pooled tumouroid samples (each generated from a single HB), four HB, and four adjacent non-tumour liver tissues. (B) MA plot showing gene expression differences between each pool of tumouroids and its corresponding HB-like tumour. Each dot represents a gene, plotted as $\log_2$ fold change *vs*. mean normalised counts across all samples. (C) Heatmap of differentially expressed genes between four pools of tumouroids, four HB, and four adjacent non-tumour liver tissues. Samples were clustered using unsupervised hierarchical clustering. (D) GSEA highlighting significantly enriched signatures between murine tumouroids and human HB tissues. Enrichment results are shown for selected gene sets, with NES, NOM *p* values, and FDR q-values indicated. FDR, false discovery rate; GSEA, gene set enrichment analysis; HCC, hepatocellular carcinoma; NES, normalised enrichment score; NOM, nominal; PCA, principal component analysis.

organisation provides a physiologically relevant platform to investigate tumour cell plasticity and tumour-microenvironment interactions. Although circulating immune cell populations are not represented in the current model, limiting studies of immune-mediated therapeutic responses, future incorporation of additional immune components should broaden its utility for immuno-oncology research.

Preclinical modelling of β-catenin-driven primary liver cancers remains challenging. Conventional liver cancer cell lines are readily amenable to drug screening but fail to reproduce the cellular heterogeneity and tissue architecture of primary tumours. Patient-derived xenografts retain these characteristics but are constrained by variable engraftment efficiency, high costs, lengthy experimental timelines and limited compatibility with medium-throughput therapeutic testing. Patient-derived tumouroids have emerged as an attractive complementary approach, particularly following the pioneering work of Huch and colleagues demonstrating long-term expansion of primary HCC and cholangiocarcinoma organoids.[24] However, subsequent studies consistently reported greater success in establishing tumouroids from poorly differentiated HCC than from well-differentiated *CTNNB1*-mutated tumours, leaving an

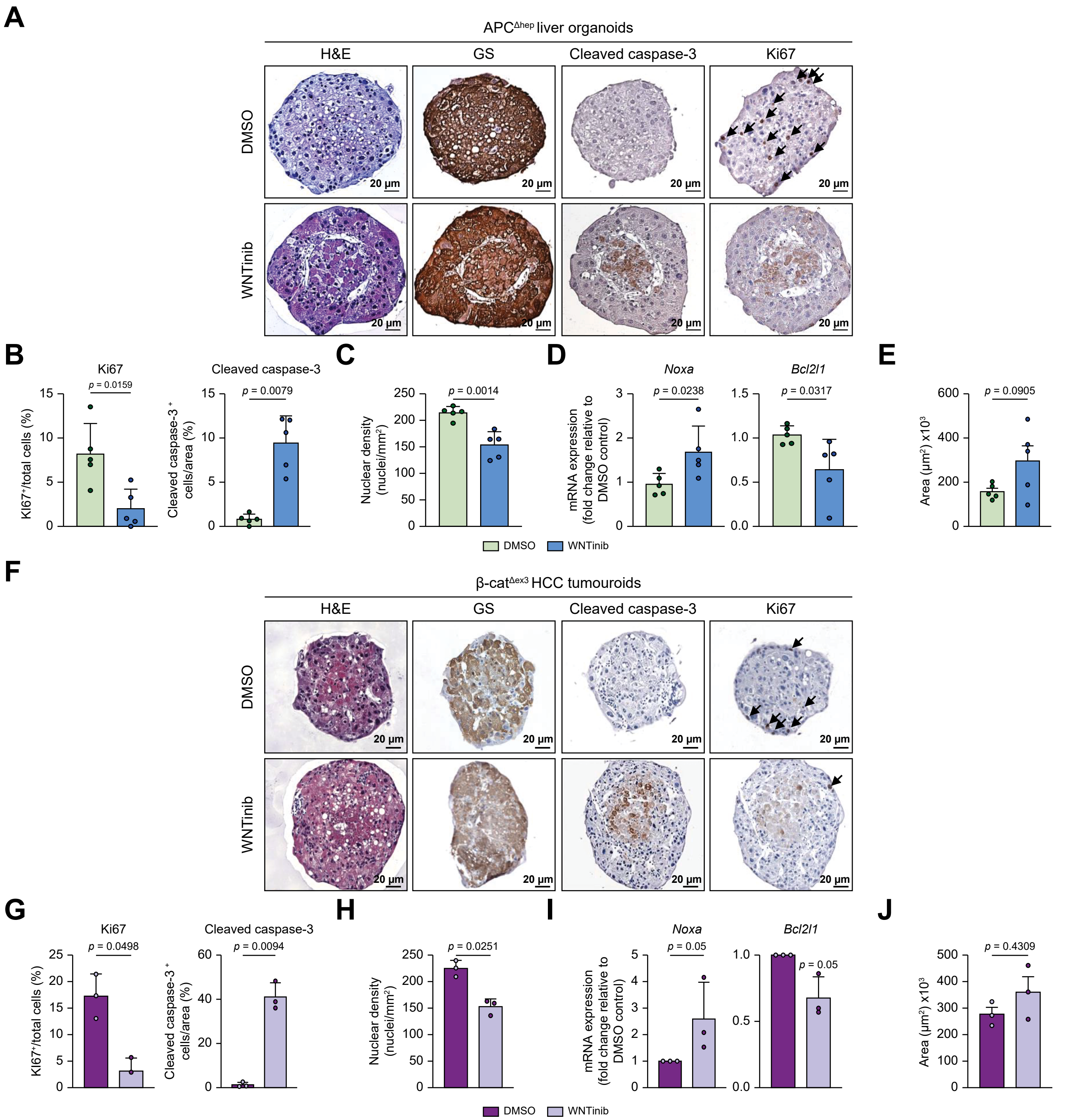

Fig. 7. WNTinib induces apoptosis and impairs proliferation in APC$^{\Delta hep}$ liver organoids and βcat$^{\Delta ex3}$ HCC tumouroids. (A) Representative H&E staining and immunostaining in APC$^{\Delta Hep}$ liver organoids after 8 days of culture in the ClinoStar and treated with WNTinib (1 μM) or DMSO for 5 additional days. Black arrows indicate Ki67-positive nuclei. (B) Quantification of immunostaining. (C) Quantification of nuclear density. (D) Relative transcript expression assessed by RT-qPCR. (E) Quantification of projected organoid area. n = 5 biological replicates, one mouse per replicate. For each biological replicate, RNA was extracted from a pool of 20-25 organoids and 8-15 tumouroids were analysed for protein expression. (F) Representative H&E staining and immunostaining in βcat$^{\Delta ex3}$ HCC tumouroids after 8 days of culture in the ClinoStar system and treated with WNTinib (5 μM) or DMSO for 5 additional days. Black arrows indicate Ki67-positive nuclei. (G) Quantification of immunostaining. (H) Quantification of nuclear density. (I) Relative transcript expression assessed by RT-qPCR. (J) Quantification of projected organoid area. n = 3 biological replicates, one mouse per replicate. For each biological replicate, RNA was extracted from a pool of 20-25 organoids and 5-10 tumouroids were analysed for protein expression. Values are mean ± SD. Differences were assessed using the Mann-Whitney test. HCC, hepatocellular carcinoma; RT-qPCR, reverse-transcription quantitative PCR.

important unmet need in available experimental models.[25,29,30] Likewise, although several groups have established human HB organoids,[26–28] transcriptomic information remains limited for tumours displaying mesenchymal features.

Our models directly address these limitations. Transcriptomic analyses demonstrated that murine HCC and HB-like tumouroids were enriched for molecular signatures previously associated with human *CTNNB1*-mutated HCC and mesenchymal HB, respectively. These findings indicate that the biological programmes in these murine tumours parallel those observed in their human counterparts, supporting the translational relevance of the models. The ability to reproducibly generate tumouroids representative of clinically relevant tumour subtypes should facilitate mechanistic studies while expanding the repertoire of preclinical models available for β-catenin-driven liver cancers.

A major objective of developing these organoids and tumouroids was to establish a biologically relevant platform for preclinical drug evaluation. Consistent with this goal, both preneoplastic organoids and HCC tumouroids responded to WNTinib, a recently described transcriptional inhibitor of mutant β-catenin, with reduced proliferation and increased apoptosis, in agreement with previous findings obtained in matrix-embedded organoid models.[45,46] In contrast, sorafenib showed limited activity under our experimental conditions, whereas TSA induced a marked pro-apoptotic response. These results demonstrate that the ClinoStar system can discriminate between compounds with distinct therapeutic effects while preserving clinically relevant tumour features.

Although the number of tumouroids obtained from an individual mouse tumour is inherently finite, the ClinoStar platform routinely produces sufficient tumouroids (typically 30-75 per tumour) that retain the molecular identity of the tumour of origin. Individual tumouroids can be distributed into multiwell plates using a dedicated dispensing system and exposed to different treatment conditions, enabling medium-throughput drug testing and functional prioritisation of candidate therapies. By maximising experimental information obtained from each tumour, this approach also contributes to the principle of reduction in animal research.

In conclusion, we describe a robust matrix-free methodology for generating murine tumouroids from β-catenin-driven HCC and HB. These models recapitulate tumour architecture, cellular heterogeneity and clinically relevant molecular features while providing a practical platform for histological, molecular and pharmacological investigations. By bridging genetically engineered mouse models and functional preclinical studies, this approach provides a valuable resource for investigating liver tumourigenesis and accelerating therapeutic development. Extending this strategy to additional murine liver cancer models and, ultimately, to human liver tumours could further broaden its translational applications.

Affiliations

[1]*INSERM, Université Paris Cité, Sorbonne Université, Centre de Recherche des Cordeliers, F-75006 Paris, France;* [2]*Team « Oncogenic functions of beta-catenin signaling in the liver », Équipe labellisée par la Ligue Nationale contre le Cancer, F-75013 Paris, France;* [3]*AP-HP, Institut du Cancer Paris CARPEM, F-75015 Paris, France;* [4]*Projet de recherche Enfants, Adolescents et Cancer Ligue Contre le Cancer, France;* [5]*INSERM, CNRS, Université Paris Cité, Institut Cochin, Plateforme Imagerie du vivant, F-75014 Paris, France;* [6]*AP-HP, Hôpital Saint-Antoine, F-75012 Paris, France;* [7]*AP-HP, Hôpital Henri Mondor, Département d'anatomopathologie, F-94000 Créteil, France;* [8]*Faculté de médecine, Université de Montréal, Département de pathologie et biologie cellulaire, Montréal, Québec, Canada;* [9]*INSERM, Institut Mondor de Recherche Biomédicale, F-94000 Créteil, France*

Abbreviations

AAV8, adeno-associated virus serotype 8; AdCre, adenovirus encoding Cre recombinase; CA9, carbonic anhydrase; GS, glutamine synthetase; HB, hepatoblastoma; IHC, immunohistochemistry; NPC, non-parenchymal cells; PDX, patient-derived xenograft; TSA, trichostatin A; ULA, ultra-low attachment; WT, wild-type.

Financial support

This study has been supported by grants from Institut National de la Santé et de la Recherche Médicale (INSERM), Association Française pour l'Etude du Foie (AFEF, to CDM), Institut National du Cancer (to CDM and VBM, PLBIO 24-107, and to SC and VBM, PLBIO 21-268, INCa_1602), and Ligue Nationale Contre le Cancer (to SC and VBM, AAPEAC2022.LCC/VM). VBM salary is funded by Ligue Nationale Contre le Cancer (AAPEAC2022.LCC/VM, INCa_1602 PLBIO 21-268, and by INCa PLBIO 24-107).

Conflict of interest

The authors declare no conflicts of interest pertaining to this manuscript.

Please refer to the accompanying ICMJE disclosure forms for further details.

Authors' contributions

C.D.-M. and V.B.M. conceived the study and supervised all aspects of the project. V.B.M., F.L. and A.G. performed hepatocyte isolation and cell culture experiments. V.B.M. and A.H. performed histology, immunodetection and RT-qPCR. I.L. performed liver ultrasonography. SB followed tumour progression by ultrasonography. A.A.-M. and J.A. analyzed IHC staining. A.G. managed $Apc^{fl/fl}$ mouse breeding. C.D.-M. and S.C. obtained funding supports. C.D.-M. and V.B.M. wrote the manuscript with contributions from all authors. All authors read and approved the final manuscript.

Data availability

The sequencing data generated in this study have been deposited in ENA under accession number PRJEB115263. Other data supporting the findings of this study are available from the corresponding author upon reasonable request.

Acknowledgements

We are grateful to CRC core facilities (INSERM UMRS_1138, Sorbonne Université, Université Paris Cité) and their team members for animal care and breeding (CFE, Mrs Sonia Prince), genotyping (CGB, Mrs Hermine Kakanakou), and cell imaging and COMET (CHICS, Dr Christophe Klein and Mrs Floriane Arbaretaz). We warmly thank the Institut Cochin's core facilities (INSERM U1016, CNRS UMR8104, Université Paris Cité) and their team members for animal care and breeding (Moust'IC, Mr Marcio Do-Cruzeiro), liver ultrasonography (PIV, Dr Gilles Renault) and transcriptomic analysis (Genom'IC, Dr Franck Letourneur). Dr Véronique Blouin (CAPACITES, INSERM UMR1089, Université de Nantes, France) is thanked for recombinant AAV8 production. Dr Ronan Le Bouffant and Dr Helle Sedighi Frandsen are thanked for critical reading of the manuscript.

Supplementary data

Supplementary data to this article can be found online at https://doi.org/10.1016/j.jhepr.2026.102040.

References

Author names in bold designate shared co-first authorship

[1] Vogel A, Meyer T, Sapisochin G, et al. Hepatocellular carcinoma. Lancet 2022;400:1345–1362.

[2] Jeong SU, Kang HJ. Recent updates on the classification of hepatoblastoma according to the International Pediatric liver Tumors Consensus. J Liver Cancer 2022;22:23–29.
[3] Xu C, Xu Z, Zhang Y, et al. beta-Catenin signaling in hepatocellular carcinoma. J Clin Invest 2022;132.
[4] Lehrich BM, Monga SP. WNT-beta-catenin signalling in hepatocellular carcinoma: from bench to clinical trials. Nat Rev Gastroenterol Hepatol 2026;23:246–263.
[5] Harding JJ, Nandakumar S, Armenia J, et al. Prospective genotyping of hepatocellular carcinoma: clinical implications of next-generation sequencing for Matching patients to targeted and immune therapies. Clin Cancer Res 2019;25:2116–2126.
[6] Luke JJ, Bao R, Sweis RF, et al. WNT/beta-catenin pathway activation Correlates with immune exclusion across human cancers. Clin Cancer Res 2019;25:3074–3083.
[7] Pinyol R, Sia D, Llovet JM. Immune exclusion-Wnt/CTNNB1 Class Predicts resistance to Immunotherapies in HCC. Clin Cancer Res 2019;25:2021–2023.
[8] Ruiz de Galarreta M, Bresnahan E, Molina-Sanchez P, et al. Beta-catenin activation promotes immune Escape and resistance to Anti-PD-1 Therapy in hepatocellular carcinoma. Cancer Discov 2019;9:1124–1141.
[9] Hooks KB, Audoux J, Fazli H, et al. New insights into diagnosis and therapeutic options for proliferative hepatoblastoma. Hepatology 2018;68:89–102.
[10] Cairo S, Armengol C, De Reynies A, et al. Hepatic stem-like phenotype and interplay of Wnt/beta-catenin and Myc signaling in aggressive childhood liver cancer. Cancer Cell 2008;14:471–484.
[11] **Hirsch TZ**, **Pilet J**, **Morcrette G**, et al. Integrated Genomic analysis identifies driver genes and cisplatin-Resistant Progenitor phenotype in Pediatric liver cancer. Cancer Discov 2021;11:2524–2543.
[12] Nagae G, Yamamoto S, Fujita M, et al. Genetic and epigenetic basis of hepatoblastoma diversity. Nat Commun 2021;12:5423.
[13] Sumazin P, Chen Y, Trevino LR, et al. Genomic analysis of hepatoblastoma identifies distinct molecular and prognostic subgroups. Hepatology 2017;65:104–121.
[14] Chen Z, Dong R. Advances in the conventional clinical treatment for hepatoblastoma and therapeutic innovation. World J Pediatr Surg 2021;4:e000220.
[15] Colnot S, Decaens T, Niwa-Kawakita M, et al. Liver-targeted disruption of Apc in mice activates beta-catenin signaling and leads to hepatocellular carcinomas. Proc Natl Acad Sci U S A 2004;101:17216–17221.
[16] Loesch R, Caruso S, Paradis V, et al. Deleting the beta-catenin degradation domain in mouse hepatocytes drives hepatocellular carcinoma or hepatoblastoma-like tumor growth. J Hepatol 2022;77:424–435.
[17] Sanceau J, Poupel L, Joubel C, et al. DLK1/DIO3 locus upregulation by a beta-catenin-dependent enhancer drives cell proliferation and liver tumorigenesis. Mol Ther 2024;32:1125–1143.
[18] **Gougelet A**, **Sartor C**, **Senni N**, et al. Hepatocellular carcinomas with mutational activation of beta-catenin Require choline and can Be detected by Positron Emission Tomography. Gastroenterology 2019;157:807–822.
[19] Gougelet A, Sartor C, Bachelot L, et al. Antitumour activity of an inhibitor of miR-34a in liver cancer with beta-catenin-mutations. Gut 2016;65:1024–1034.
[20] Zhang G, Liang Q, Wu Y, et al. Insights on the differences between two- and three-dimensional culture systems in tumor models. Int J Mol Med 2025;56:185.
[21] Abuwatfa WH, Pitt WG, Husseini GA. Scaffold-based 3D cell culture models in cancer research. J Biomed Sci 2024;31:7.
[22] Urzi O, Gasparro R, Costanzo E, et al. Three-Dimensional cell cultures: the bridge between in vitro and in vivo models. Int J Mol Sci 2023;24.
[23] Lee SY, Koo IS, Hwang HJ, et al. In Vitro three-dimensional (3D) cell culture tools for spheroid and organoid models. SLAS Discov 2023;28:119–137.
[24] Broutier L, Mastrogiovanni G, Verstegen MM, et al. Human primary liver cancer-derived organoid cultures for disease modeling and drug screening. Nat Med 2017;23:1424–1435.
[25] Nuciforo S, Fofana I, Matter MS, et al. Organoid models of human liver cancers derived from tumor Needle Biopsies. Cell Rep 2018;24:1363–1376.
[26] Saltsman JA, Hammond WJ, Narayan NJC, et al. A human organoid model of aggressive hepatoblastoma for disease modeling and drug testing. Cancers (Basel) 2020;12.
[27] **Song H**, **Bucher S**, Rosenberg K, et al. Single-cell analysis of hepatoblastoma identifies tumor signatures that predict chemotherapy susceptibility using patient-specific tumor spheroids. Nat Commun 2022;13:4878.
[28] Wu PV, Fish M, Hazard FK, et al. A developmental biliary lineage program cooperates with Wnt activation to promote cell proliferation in hepatoblastoma. Nat Commun 2024;15:10007.
[29] **Dai S**, **Wu J**, **Chai Y**, et al. Single-cell transcriptomic mapping of patient-derived primary liver cancer organoids reveals molecular subtypes and guides precision drug targeting. Cel Oncol (Dordr) 2026;49.
[30] Crouchet E, Almeida N, Durand SC, et al. A patient-derived HCC spheroid system to model the tumor microenvironment and treatment response. JHEP Rep 2025;7:101252.
[31] Wrzesinski K, Fey SJ. Metabolic Reprogramming and the recovery of physiological Functionality in 3D cultures in Micro-bioreactors. Bioengineering (Basel) 2018;5.
[32] Stransky S, Cutler R, Aguilan J, et al. Investigation of reversible histone acetylation and dynamics in gene expression regulation using 3D liver spheroid model. Epigenetics Chromatin 2022;15:35.
[33] Kim C, Zhu Z, Barbazuk WB, et al. Time-course characterization of whole-transcriptome dynamics of HepG2/C3A spheroids and its toxicological implications. Toxicol Lett 2024;401:125–138.
[34] Stampar M, Sedighi Frandsen H, Rogowska-Wrzesinska A, et al. Hepatocellular carcinoma (HepG2/C3A) cell-based 3D model for genotoxicity testing of chemicals. Sci Total Environ 2021;755:143255.
[35] Gougelet A, Torre C, Veber P, et al. T-cell factor 4 and beta-catenin chromatin occupancies pattern zonal liver metabolism in mice. Hepatology 2014;59:2344–2357.
[36] Bankhead P, Loughrey MB, Fernandez JA, et al. QuPath: Open source software for digital pathology image analysis. Sci Rep 2017;7:16878.
[37] Torres S, Abdullah Z, Brol MJ, et al. Recent Advances in practical methods for liver cell biology: a Short Overview. Int J Mol Sci 2020;21.
[38] Senni N, Savall M, Cabrerizo Granados D, et al. beta-catenin-activated hepatocellular carcinomas are addicted to fatty acids. Gut 2019;68:322–334.
[39] Anson M, Crain-Denoyelle AM, Baud V, et al. Oncogenic beta-catenin triggers an inflammatory response that determines the aggressiveness of hepatocellular carcinoma in mice. J Clin Invest 2012;122:586–599.
[40] Boyault S, Rickman DS, de Reynies A, et al. Transcriptome classification of HCC is related to gene alterations and to new therapeutic targets. Hepatology 2007;45:42–52.
[41] Chiang DY, Villanueva A, Hoshida Y, et al. Focal gains of VEGFA and molecular classification of hepatocellular carcinoma. Cancer Res 2008;68:6779–6788.
[42] **Lachenmayer A**, **Alsinet C**, Savic R, et al. Wnt-pathway activation in two molecular classes of hepatocellular carcinoma and experimental modulation by sorafenib. Clin Cancer Res 2012;18:4997–5007.
[43] Lehrich BM, Tao J, Liu S, et al. Development of mutated beta-catenin gene signature to identify CTNNB1 mutations from whole and spatial transcriptomic data in patients with HCC. JHEP Rep 2024;6:101186.
[44] Carrillo-Reixach J, Torrens L, Simon-Coma M, et al. Epigenetic footprint enables molecular risk stratification of hepatoblastoma with clinical implications. J Hepatol 2020;73:328–341.
[45] **Rialdi A**, **Duffy M**, **Scopton AP**, et al. WNTinib is a multi-kinase inhibitor with specificity against beta-catenin mutant hepatocellular carcinoma. Nat Cancer 2023;4:1157–1175.
[46] Balaseviciute U, Huguet-Pradell J, Abril-Fornaguera J, et al. Effective therapeutic targeting of CTNNB1-mutant hepatoblastoma with WNTinib. Mol Oncol 2026;20:920–932.
[47] Llovet JM, Ricci S, Mazzaferro V, et al. Sorafenib in advanced hepatocellular carcinoma. N Engl J Med 2008;359:378–390.
[48] Buurman R, Gurlevik E, Schaffer V, et al. Histone deacetylases activate hepatocyte growth factor signaling by repressing microRNA-449 in hepatocellular carcinoma cells. Gastroenterology 2012;143:811–820 e815.
[49] Aiello G, Nemir M, Vidimova B, et al. Increased reproducibility of brain organoids through controlled fluid dynamics. EMBO Rep 2025;26:6209–6239.
[50] Dona F, Erratico S, Terracciano LM, et al. Protocol for the establishment and characterization of lung tumoroids from surgical resection without the use of Matrigel. STAR Protoc 2025;6:103923.

Keywords: Tumouroids; 3D culture; rotating bioreactor; preclinical modeling; tumour microenvironment; therapeutic screening; translational research.

Received 2 April 2026; received in revised form 4 September 2026; accepted 10 September 2026; Available online 18 September 2026

Journal of Hepatology, Volume *8*

Supplemental information

Novel 3D models for β-catenin-driven liver cancers

Vanessa Bou Malham, Fanny Léandre, Akila Hamimi, Isabelle Lagoutte, Sandrine Bouchet, Alexandre Archambault-Marsan, Jérémy Augustin, Angélique Gougelet, Sabine Colnot, and Christèle Desbois-Mouthon

Novel 3D models for β-catenin-driven liver cancers

Vanessa Bou Malham, Fanny Léandre, Akila Hamimi, Isabelle Lagoutte, Sandrine Bouchet, Alexandre Archambault-Marsan, Jérémy Augustin, Angélique Gougelet, Sabine Colnot, Christèle Desbois-Mouthon

Table of contents

Supplementary materials and methods………………...2

Supplementary figures……..…………………………………………………………......…5

Supplementary tables……..………………………………………………………………..22

Supplementary materials and methods

2D culture of hepatocytes from APC$^{\Delta hep}$ mice. 500,000 hepatocytes were seeded onto 6-well plates coated with rat tail collagen type I (Gibco) in William's E medium with GlutaMAX™ (Gibco) supplemented with 10% fetal bovine serum (FBS), 100 U/mL penicillin, 10 µg/mL streptomycin, 4 µg/mL insulin (Sigma-Aldrich), 0.1% bovine serum albumin (BSA) (Eurobio), 0.5 µg/mL amphotericin B (Thermo Fisher Scientific), and 40 ng/mL dexamethasone (Sigma-Aldrich). Four hours later, the medium was replaced with the same medium lacking FBS, insulin, BSA and amphotericin B. For 3D culture, hepatocytes and NPC were resuspended in William's E medium with GlutaMAX™, supplemented with 10% FBS, penicillin/streptomycin, and 1% insulin-transferrin-selenium (ITS-G, Thermo Fisher Scientific).

Histology, IHC, TUNEL assay and multiplexed immunofluorescence. For IHC, liver sections were deparaffinized and rehydrated, and antigen retrieval was performed in citrate buffer (pH 6.0) for 20 min in a water bath at 95°C. Sections were blocked with TBS containing 0.1% Tween (TBST) and 5% normal goat serum (Vector Laboratories), incubated with the primary antibody for 1 h at room temperature or overnight at 4°C, depending on the antibody, washed twice with TBST, and then incubated with biotinylated secondary antibody. Immunoreactivity was detected using the VECTASTAIN® Elite ABC-HRP kit (Peroxidase; Vector Laboratories) and a 3,3′-diaminobenzidine substrate kit (Vector Laboratories). Nuclei were counterstained with Mayer's hemalun solution, and slides were dehydrated and mounted. For IHC analysis on organoids and tumouroids, samples were fixed for 1 h in 10% buffered formalin (VWR) at room temperature, washed with PBS, embedded in 2% agarose using a Leica mould, and subsequently processed for paraffin embedding. Detection of *in situ*

apoptosis was performed using the terminal deoxynucleotidyl transferase dUTP nick-end labeling (TUNEL) assay according to the manufacturer's instructions (Click-iT™ TUNEL Alexa Fluor™ 647, Thermo Fisher Scientific).

Multiplexed immunofluorescence was performed using the Comet^{TM} instrument (Lunaphore) on 5-µm paraffin-embedded sections from APC$^{\Delta hep}$ non-tumour liver tissues, HCC, and tumouroids using a 12x12 mm chamber. Slides underwent deparaffinization followed by heat-induced epitope retrieval in alkaline buffer (pH 9.0) at a temperature reaching 102°C, followed by controlled cooling before subsequent staining steps. Tissues were subjected to 16 cycles of antibody incubation/elution and image acquisition with dedicated imaging, elution, quenching, and washing buffers provided by Lunaphore. One or two primary antibodies were incubated for 8 min, followed by incubation with one or two corresponding secondary antibodies for 4 min. Fields of view were saved, and background was subtracted using the HORIZON software (Lunaphore).

RNA sequencing (RNA-seq) and analysis. FASTQ files were aligned to the Ensembl release 101 reference genome using the STAR algorithm (version 2.7.6a). Reads were quantified with RSEM (v1.3.1), and differential expression analysis was performed in R (version 3.6.3) using the DESeq2 package (version 1.26.0). Gene counts were normalized using the standard DESeq2 normalization method (median-of-ratios implemented in the DESeq function) after pre-filtering reads and genes (reads uniquely mapped to the genome, or mapped to up to 10 loci with count adjustment, and genes with at least 10 reads in at least 3 samples). Differentially expressed genes were identified using the Wald test with the contrast function, and *P* values were adjusted for multiple testing using the Benjamini-Hochberg false discovery rate (FDR)

procedure, as recommended in the DESeq2 package documentation. R scripts and analysis parameters are available at https://github.com/GENOM-IC-Cochin/RNA-Seq_analysis. Raw sequencing data generated in this study are available in the European Nucleotide Archive (ENA) under accession number PRJEB115263.

GSEA analysis. GSEA was performed using GSEA software (Broad Institute, version 4.4.0). Genes were ranked according to their $\log_2$ fold change ($\log_2$FC) obtained from the differential expression analysis, and enrichment was assessed using the GSEA Pre-ranked algorithm. Statistical significance was determined using 1,000 gene set permutations. Nominal *P* values were adjusted for multiple testing using the false discovery rate (FDR) method. Gene sets with an FDR < 0.25 were considered significantly enriched, in accordance with the Broad Institute recommendations.

Supplementary figures

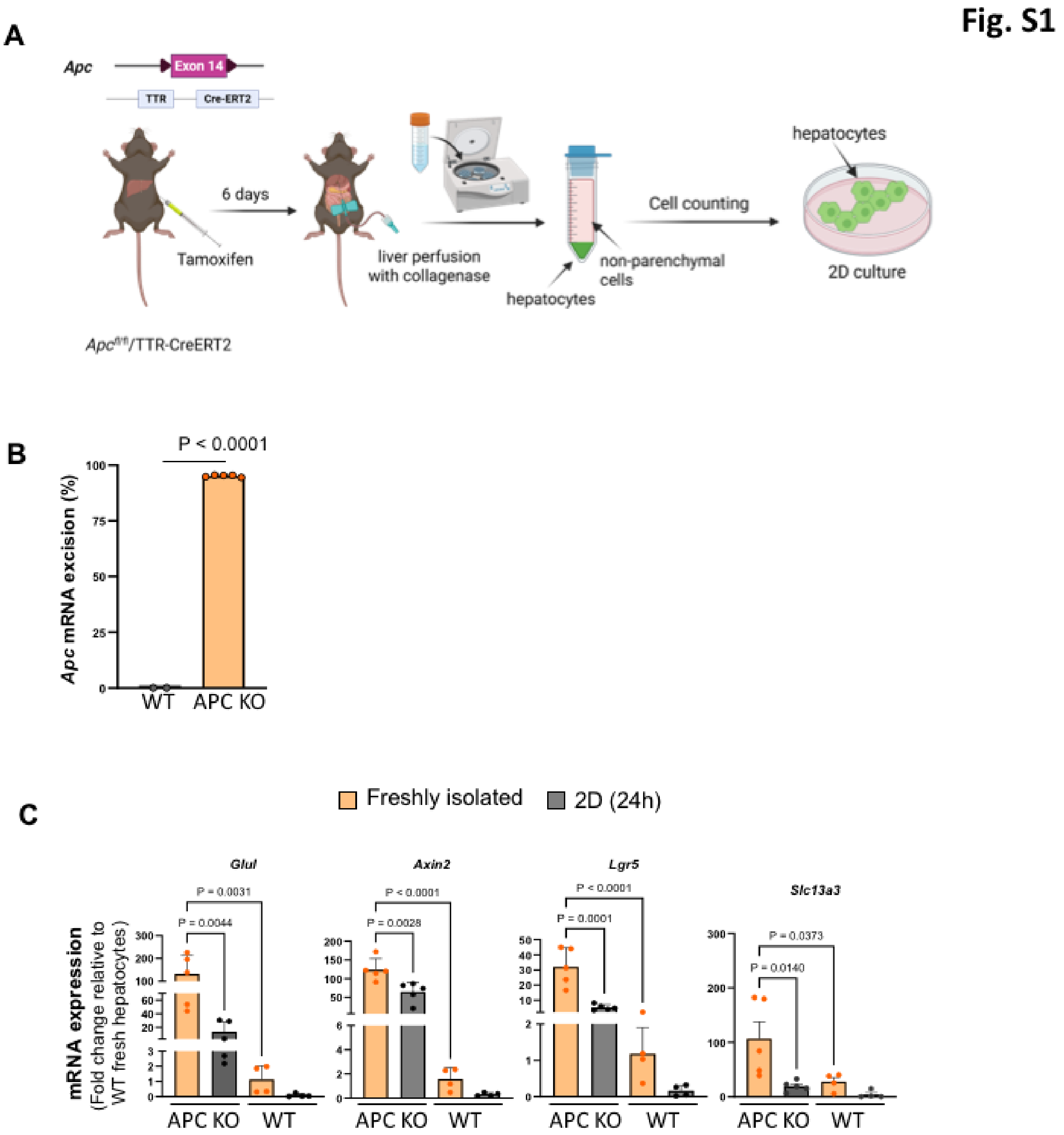

Fig. S1. 2D culture of APC^{KO} hepatocytes.

(A) Schematic illustration of the experimental design for the 2D culture of APC^{KO} hepatocytes (created with BioRender.com). **(B)** Percentage of *Apc* mRNA excision in freshly isolated wild-type (WT) and APC^{KO} hepatocytes determined by RT-qPCR (n = 5 biological replicates). **(C)** Relative transcript expression of *Glul*, *Axin2*, *Lgr5,* and *Slc13a3* assessed by RT-qPCR in freshly isolated APC^{KO} hepatocytes or after 24 h of

2D culture, normalized to WT freshly isolated hepatocytes (n = 5 biological replicates, one mouse per replicate). For each biological replicate, RNA was extracted from a pool of 20-25 organoids. Bar graphs represent mean values ± SD. Differences between two sample groups were assessed using the Mann-Whitney test. Differences among three sample groups were assessed using the Kruskal-Wallis test.

Fig. S2

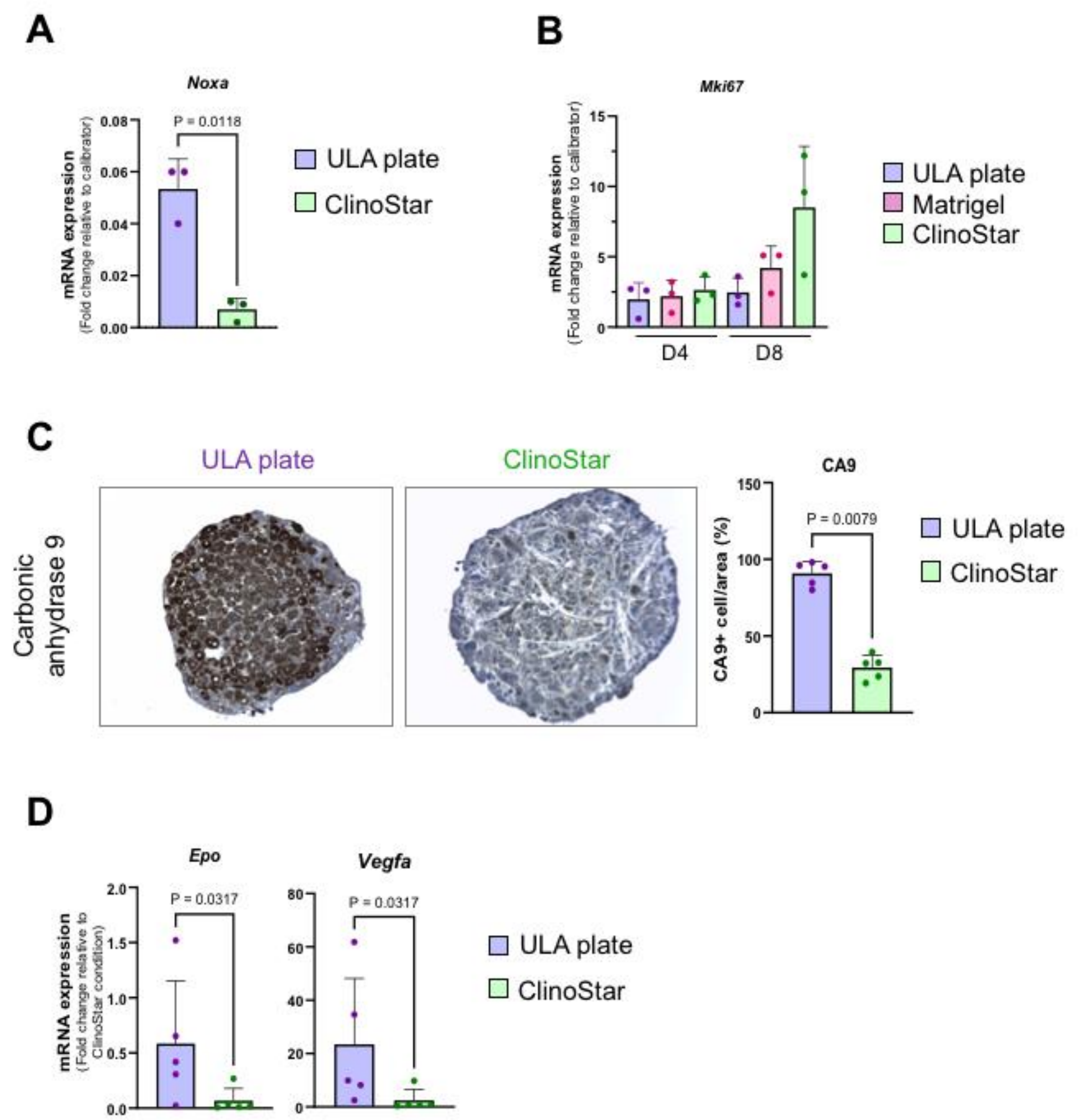

Fig. S2. Proliferation, apoptosis and hypoxia in APC$^{\Delta hep}$ liver organoids.

(A) Relative *Noxa* transcript expression assessed by RT-qPCR under the two culture conditions. Bar graphs show mean ± SD (n = 3 biological replicates, one mouse per replicate). For each biological replicate, RNA was extracted from a pool of 20-25 organoids. **(B)** Relative transcript expression of *Mki67* assessed by RT-qPCR in organoids cultured for 4 and 8 days in ULA plates, Matrigel or in the ClinoStar® system (n = 3 biological replicates, one mouse per replicate). For each biological replicate,

RNA was extracted from a pool of 20-25 organoids. Bar graphs represent mean values ± SD. **(C)** Representative immunostaining for carbonic anhydrase 9 (CA9) in $APC^{\Delta hep}$ liver organoids cultured for 8 days in ULA plates or in the ClinoStar® system (*left*), and quantification of CA9-positive cells (*right*) (n = 5 biological replicates, one mouse per replicate). For each biological replicate, 8-14 tumouroids were analyzed. **(D)** Relative transcript expression of *Epo* and *Vegfa*, assessed by RT-qPCR in organoids cultured for 8 days in ULA plates or in the ClinoStar® system (n = 5 biological replicates, one mouse per replicate). For each biological replicate, RNA was extracted from a pool of 20-25 organoids. Bar graphs represent mean values ± SD. Differences between groups were assessed using the Mann-Whitney test.

Fig. S3

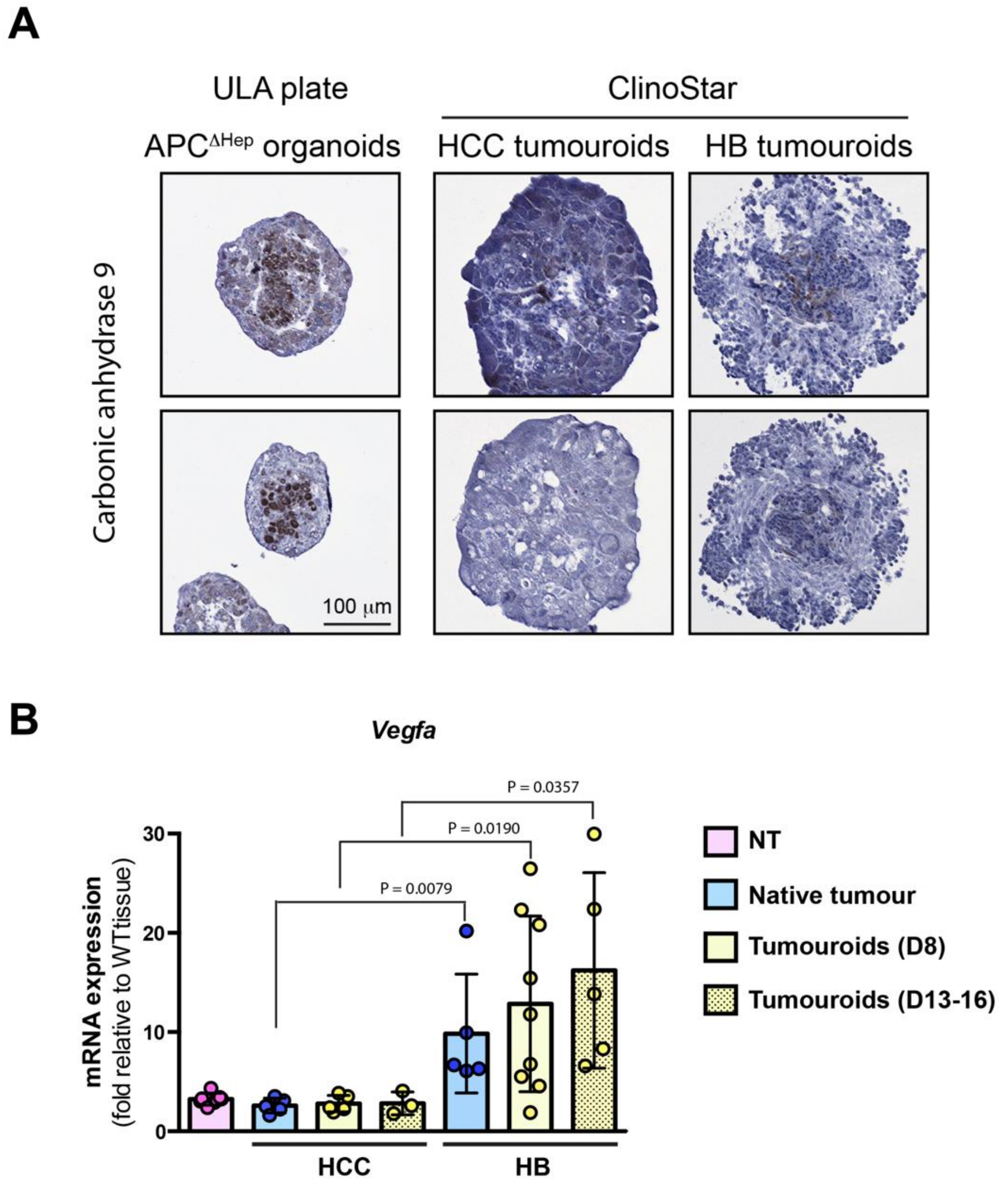

Fig. S3. Hypoxia in HCC and HB-liked tumouroids.

(A) Representative immunostaining for carbonic anhydrase 9 (CA9) in HCC and HB-liked tumouroids cultured during 16 days in the ClinoStar® system (n = 2 biological replicates, one mouse per replicate). For each biological replicate, 5-6 tumouroids were analyzed. As a positive control, APC$^{\Delta hep}$ liver organoids cultured in ULA plates are shown. **(B)** Relative transcript expression of *Vegfa*, assessed by RT-qPCR in non

tumour liver tissue (NT)s, HCC, HB, and HCC and HB-liked tumouroids cultured for 8 or 13-16 days in the ClinoStar® system (n = 3-9 biological replicates, one mouse per replicate). For each biological replicate, RNA was extracted from a pool of 20-25 organoids. Bar graphs represent mean values ± SD. Differences between groups were assessed using the Mann-Whitney test.

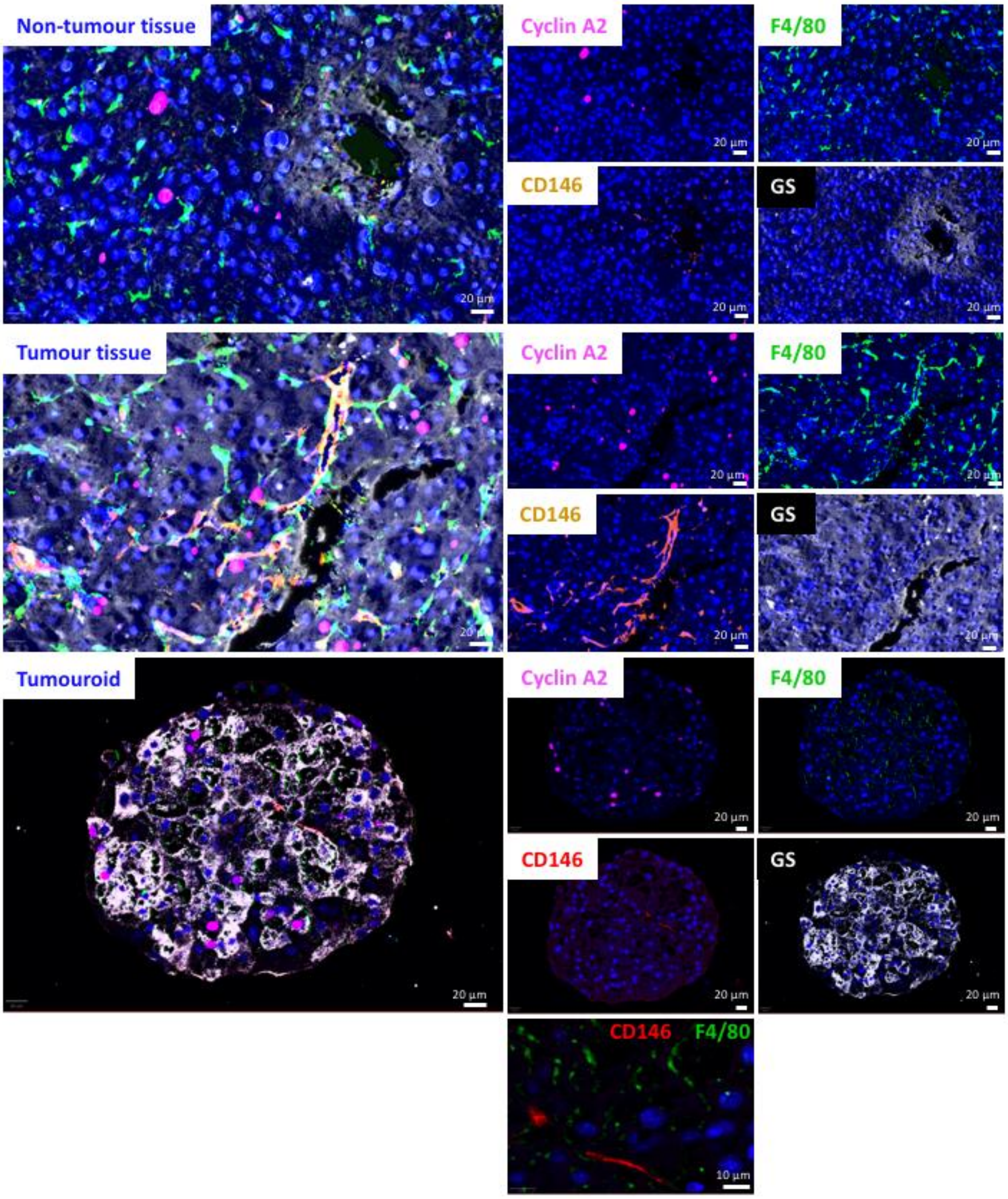

Fig. S4. Multiplex tissue profiling of HCC-derived tumouroids.

Representative images of multiplex immunofluorescence performed using COMET^{TM} on non-tumour liver tissue, HCC and HCC-derived tumouroid from APC$^{\Delta hep}$ mouse

showing cyclin A2 (magenta), CD146 (red), GS (white), F4/80 (green), and nuclei counterstained with DAPI (blue).

Fig. S5

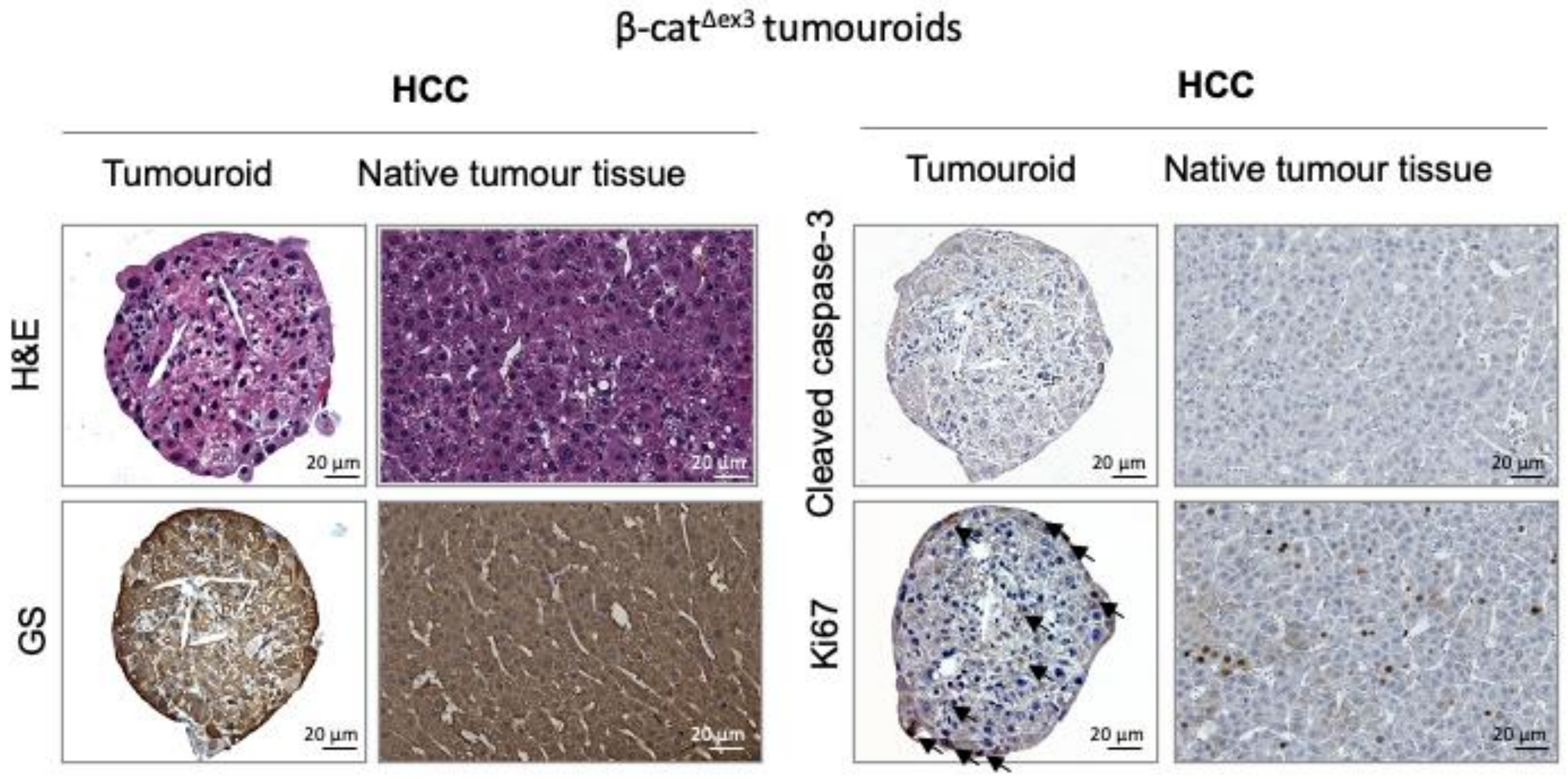

Fig.S5. Histological and IHC characterization of βcat$^{\Delta ex3}$ tumouroids.

Representative sections of H&E staining and immunostaining for cleaved capase-3, GS, and Ki67 in β-cat$^{\Delta ex3}$ tumouroids formed after 8 days of culture in the ClinoStar® system. Black arrows indicate Ki67-positive nuclei. n = 3 biological replicates, one mouse per replicate. For each biological replicate, 4-6 tumouroids were analyzed.

Fig. S6

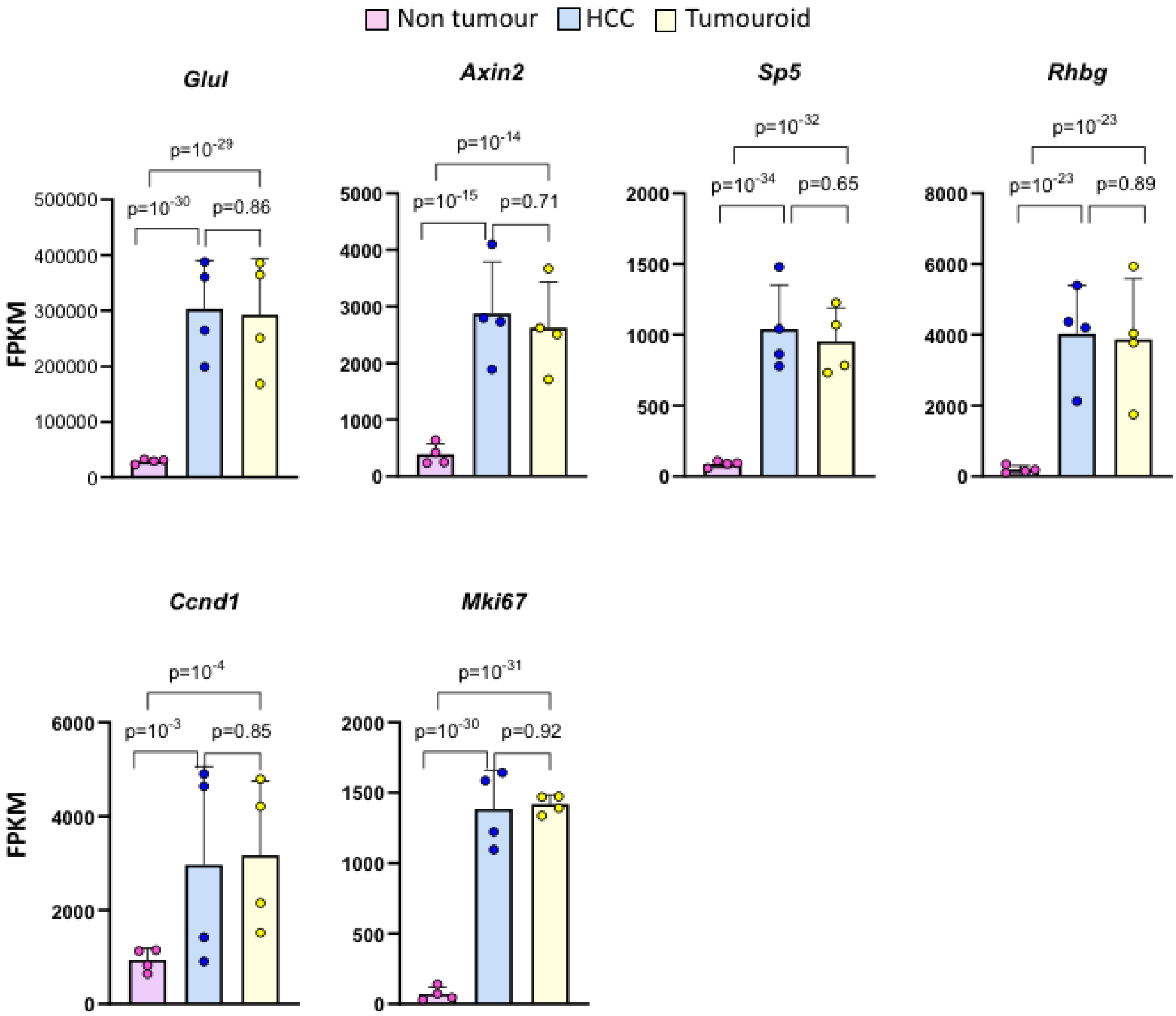

Fig. S6. Expression of proliferation- and β-catenin-related genes in HCC-derived APC$^{\Delta hep}$ tumouroids.

RNA-seq data (FPKM) showing the upregulation of *Glul*, *Axin2*, *Sp5*, *Rhbg*, *Ccnd1* and *Mki67* in murine HCC-derived APC$^{\Delta hep}$ tumouroids compared with adjacent non-tumour liver tissue and HCC**.** Differences among groups were assessed using the Kruskal-Wallis test.

Fig. S7

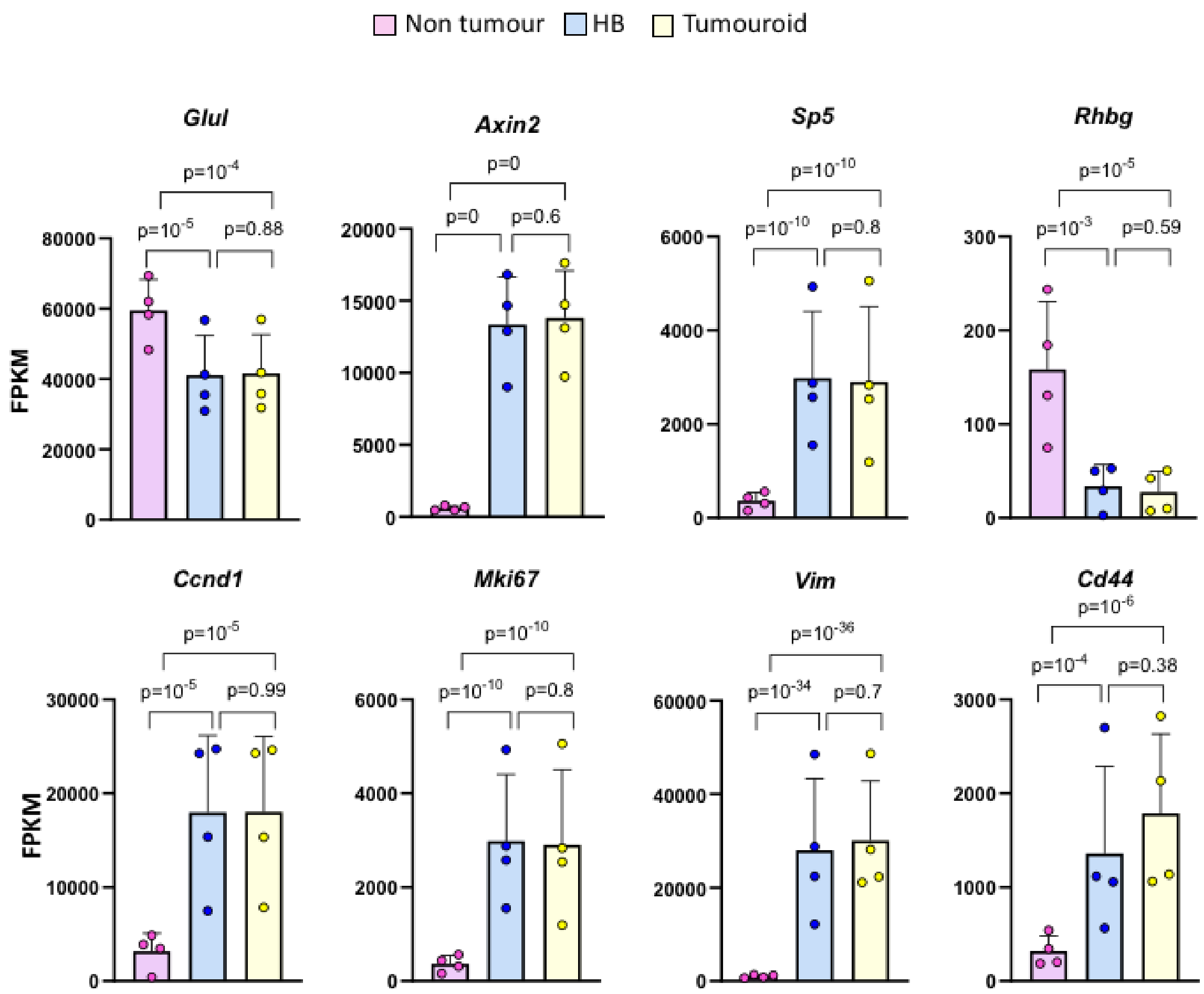

Fig. S7. Expression of proliferation-, β-catenin- and mesenchyme-related genes in HB-liked APC$^{\Delta hep}$ tumouroids.

RNA-seq data (FPKM) showing the status of *Glul*, *Axin2*, *Sp5*, *Rhbg*, *Ccnd1*, *Mki67, Vim* and *Cd44* in murine HB-liked APC$^{\Delta hep}$ tumouroids compared with adjacent non-tumour liver tissue and HB**.** Differences among groups were assessed using the Kruskal-Wallis test.

Fig. S8

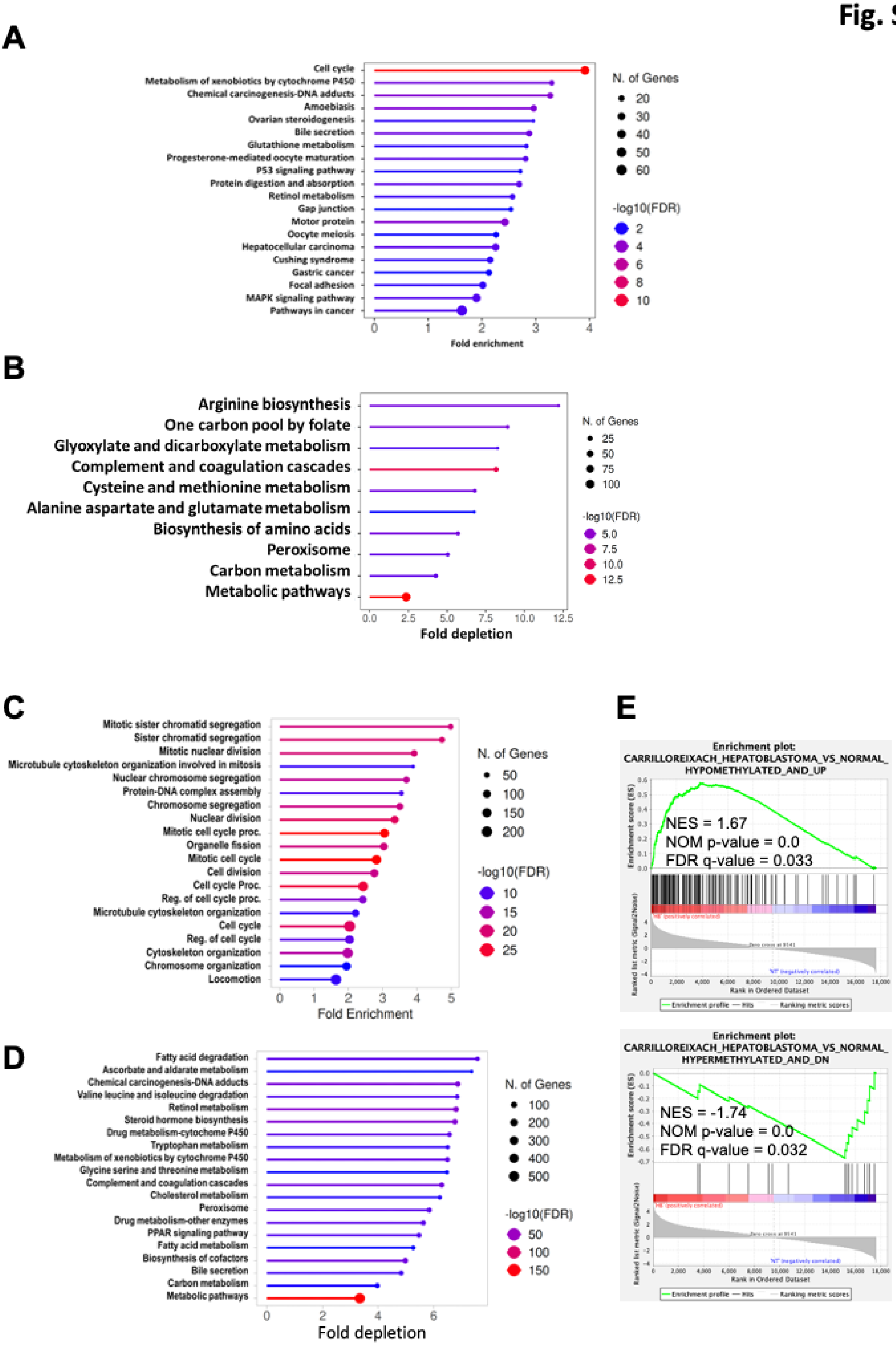
A
Cell cycle
Metabolism of xenobiotics by cytochrome P450
Chemical carcinogenesis-DNA adducts
Amoebiasis
Ovarian steroidogenesis
Bile secretion
Glutathione metabolism
Progesterone-mediated oocyte maturation
P53 signaling pathway
Protein digestion and absorption
Retinol metabolism
Gap junction
Motor protein
Oocyte meiosis
Hepatocellular carcinoma
Cushing syndrome
Gastric cancer
Focal adhesion
MAPK signaling pathway
Pathways in cancer
0 1 2 3 4
Fold enrichment
N. of Genes
20
30
40
50
60
-log10(FDR)
2
4
6
8
10
B
Arginine biosynthesis
One carbon pool by folate
Glyoxylate and dicarboxylate metabolism
Complement and coagulation cascades
Cysteine and methionine metabolism
Alanine aspartate and glutamate metabolism
Biosynthesis of amino acids
Peroxisome
Carbon metabolism
Metabolic pathways
0.0 2.5 5.0 7.5 10.0 12.5
Fold depletion
N. of Genes
25
50
75
100
-log10(FDR)
5.0
7.5
10.0
12.5
C
Mitotic sister chromatid segregation
Sister chromatid segregation
Mitotic nuclear division
Microtubule cytoskeleton organization involved in mitosis
Nuclear chromosome segregation
Protein-DNA complex assembly
Chromosome segregation
Nuclear division
Mitotic cell cycle proc.
Organelle fission
Mitotic cell cycle
Cell division
Cell cycle Proc.
Reg. of cell cycle proc.
Microtubule cytoskeleton organization
Cell cycle
Reg. of cell cycle
Cytoskeleton organization
Chromosome organization
Locomotion
0 1 2 3 4 5
Fold Enrichment
N. of Genes
50
100
150
200
-log10(FDR)
10
15
20
25
D
Fatty acid degradation
Ascorbate and aldarate metabolism
Chemical carcinogenesis-DNA adducts
Valine leucine and isoleucine degradation
Retinol metabolism
Steroid hormone biosynthesis
Drug metabolism-cytochrome P450
Tryptophan metabolism
Metabolism of xenobiotics by cytochrome P450
Glycine serine and threonine metabolism
Complement and coagulation cascades
Cholesterol metabolism
Peroxisome
Drug metabolism-other enzymes
PPAR signaling pathway
Fatty acid metabolism
Biosynthesis of cofactors
Bile secretion
Carbon metabolism
Metabolic pathways
0 2 4 6
Fold depletion
N. of Genes
100
200
300
400
500
-log10(FDR)
50
100
150
E
Enrichment plot:
CARRILLOREIXACH_HEPATOBLASTOMA_VS_NORMAL_HYPOMETHYLATED_AND_UP
NES = 1.67
NOM p-value = 0.0
FDR q-value = 0.033
Rank in Ordered Dataset
Enrichment plot:
CARRILLOREIXACH_HEPATOBLASTOMA_VS_NORMAL_HYPERMETHYLATED_AND_DN
NES = -1.74
NOM p-value = 0.0
FDR q-value = 0.032
Rank in Ordered Dataset

Fig. S8. KEGG pathway enrichment and GSEA analyses in murine HCC and HB-derived tumoroids

(A) KEGG pathway enrichment analysis of genes significantly upregulated in murine HCC-derived tumouroids compared to adjacent non-tumoural livers. **(B)** KEGG pathway enrichment analysis of genes significantly downregulated in HCC-derived tumouroids compared to adjacent non-tumoural livers. **(C)** KEGG pathway enrichment analysis of genes significantly upregulated in murine HB-derived tumouroids compared to adjacent non-tumoural livers. **(D)** KEGG pathway enrichment analysis of genes significantly downregulated in HB-liked tumouroids compared to adjacent non-tumoural livers. **(E)** GSEA highlighting significantly enriched signatures between murine tumouroids and human HB tissues. Enrichment results are shown for selected gene sets, with normalized enrichment scores (NES), nominal (NOM) p-values, and false discovery rate (FDR) q-values indicated.

Fig. S9

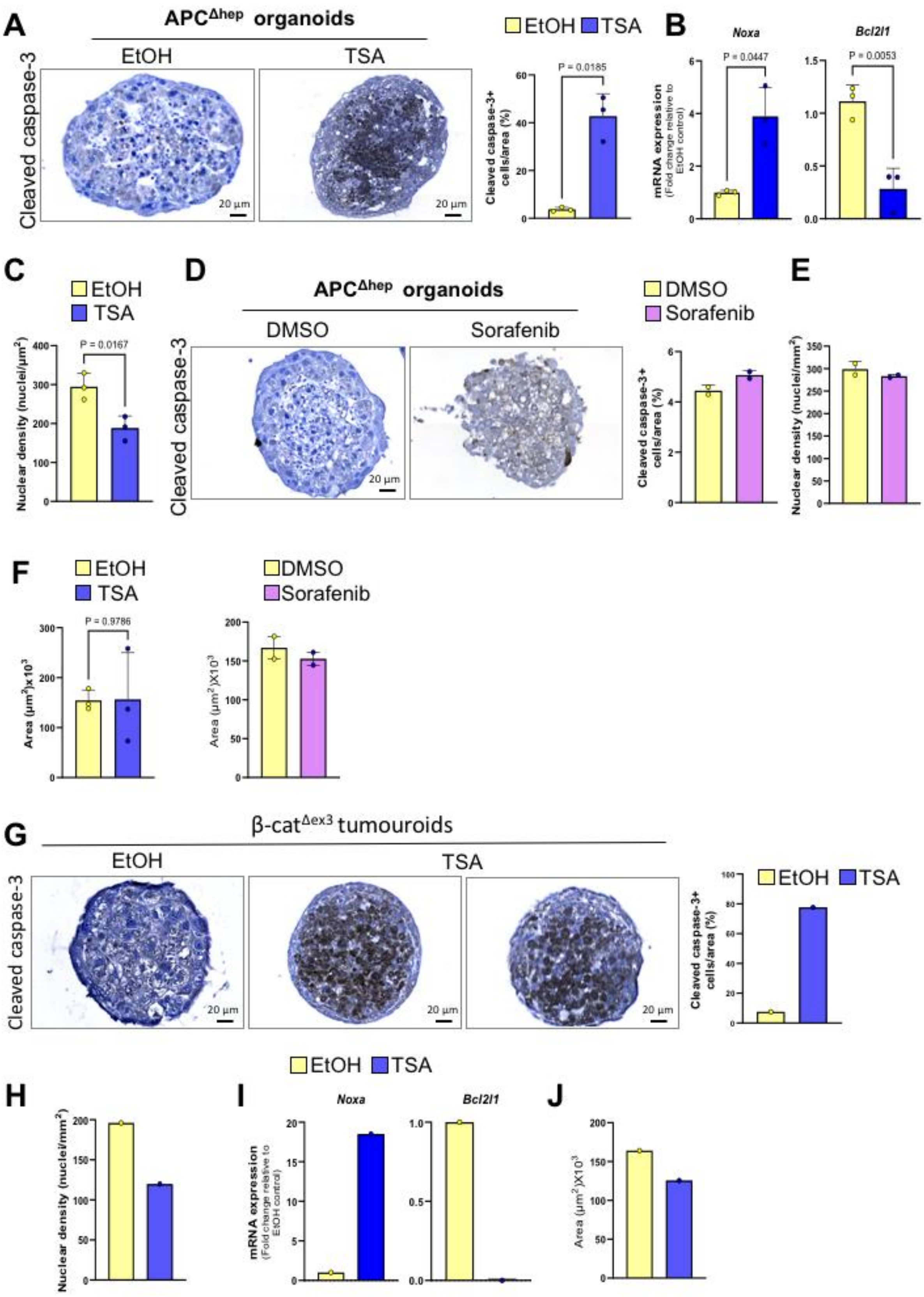

A
APC$^{\Delta hep}$ organoids
EtOH
TSA
Cleaved caspase-3
20 µm
EtOH TSA
P = 0.0185
Cleaved caspase-3+ cells/area (%)
B
Noxa
Bcl2l1
P = 0.0447
P = 0.0053
mRNA expression (Fold change relative to EtOH control)
C
EtOH
TSA
P = 0.0167
Nuclear density (nuclei/µm^2)
D
APC$^{\Delta hep}$ organoids
DMSO
Sorafenib
Cleaved caspase-3
20 µm
DMSO
Sorafenib
Cleaved caspase-3+ cells/area (%)
E
Nuclear density (nuclei/mm^2)
F
EtOH
TSA
DMSO
Sorafenib
P = 0.9786
Area (µm^2)x10^3
G
β-cat$^{\Delta ex3}$ tumouroids
EtOH
TSA
Cleaved caspase-3
20 µm
EtOH TSA
Cleaved caspase-3+ cells/area (%)
EtOH TSA
H
Nuclear density (nuclei/mm^2)
I
Noxa
Bcl2l1
mRNA expression (Fold change relative to EtOH control)
J
Area (µm^2)X10^3

Fig.S9. Effects of TSA and sorafenib in APC$^{\Delta hep}$ liver organoids and βcat$^{\Delta ex3}$ HCC tumouroids.

(A) Representative immunostaining for cleaved caspase-3 in APC$^{\Delta hep}$ liver organoids generated after 8 days of culture in the ClinoStar® system and treated with trichostatin A (TSA, 0.5 µM) or ethanol (EtOH) for 5 additional days (*left*), with quantification (*right*) (n = 3 biological replicates, one mouse per replicate). For each biological replicate, 7-14 tumouroids were analyzed. **(B)** Relative *Noxa* and *Bcl2l1* transcript expression assessed by RT-qPCR in TSA- or vehicule-treated APC$^{\Delta hep}$ organoids (n = 3 biological replicates, one mouse per replicate). For each biological replicate, RNA was extracted from a pool of 20-25 organoids. **(C, E)** Quantification of nuclear density (n = 3 biological replicates, one mouse per replicate). For each biological replicate, 7-14 tumouroids were analyzed. **(D)** Representative immunostaining for cleaved caspase-3 in APC$^{\Delta hep}$ liver organoids generated after 8 days of culture in the ClinoStar® system and treated with sorafenib (5 µM) or DMSO for 5 additional days (*left*), with quantification (*right*) (n = 2 biological replicates, one mouse per replicate). For each biological replicate, 6-10 tumouroids were analyzed. **(F)** Quantification of projected organoid area (n = 2-3 biological replicates, one mouse per replicate). For each biological replicate, 6-10 tumouroids were analyzed. **(G)** Representative immunostaining for cleaved caspase-3 in HCC tumouroids generated after 8 days of culture in the ClinoStar® and treated with trichostatin A (TSA, 0.5 µM) or ethanol (EtOH) for 5 days (*left*), with quantification (*right*) (n = 1 mouse). A total of 15 tumouroids was analyzed. **(H)** Quantification of nuclear density (n = 1 mouse). A total of 15 tumouroids were analyzed. **(I)** Relative *Noxa* and *Bcl2l1* transcript expression assessed by RT-qPCR in TSA- or ethanol-treated βcat$^{\Delta ex3}$ HCC tumouroids (n = 1 mouse). RNA was extracted from a pool of 20-25 organoids. **(J)** Quantification of projected organoid area (n = 1 mouse). 15

tumouroids were analyzed. Bar graphs represent mean values ± SD. Differences between groups were assessed using the Mann-Whitney test.

Fig. S10

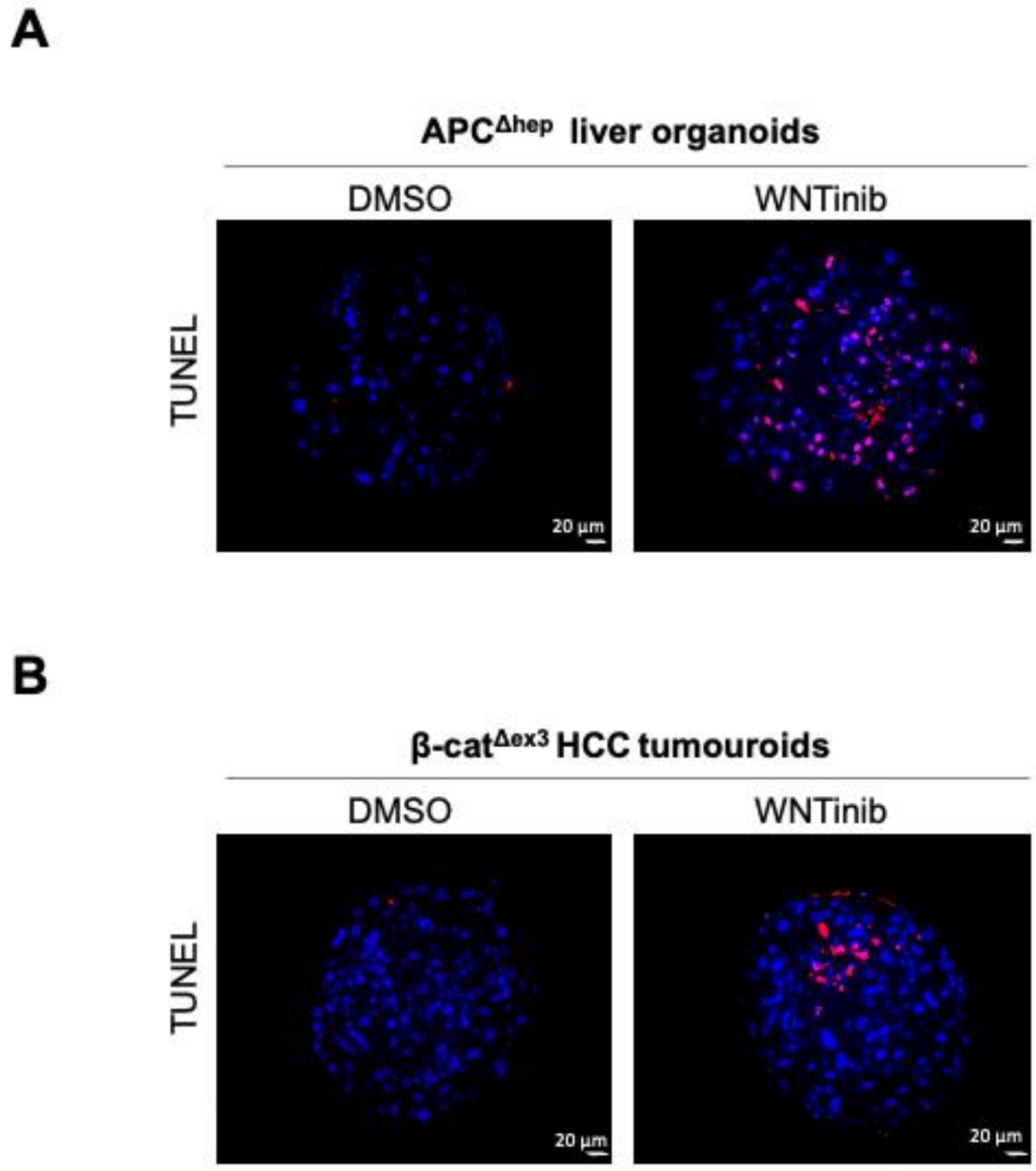

Fig.S10. TUNEL assay in Apc$^{\Delta hep}$ liver organoids and βcat$^{\Delta ex3}$ HCC tumouroids.

(A) Representative images of Apc$^{\Delta hep}$ liver organoids cultured during 8 days in the ClinoStar® system and treated with WNTinib (1 µM) or DMSO for 5 days. Apoptotic cells were detected by TUNEL staining (red), with nuclei counterstained with DAPI (blue) (n = 2 biological replicates, one mouse per replicate). For each biological replicate, 5 tumouroids were analyzed. **(B)** Representative images of β-cat$^{\Delta ex3}$ HCC tumouroids cultured during 8 days in the ClinoStar® system and treated with WNTinib (5 µM) or DMSO for 5 days. Apoptotic cells were detected by TUNEL staining (red), with nuclei counterstained with DAPI (blue) (n = 2 biological replicates, one mouse per replicate). For each biological replicate, 5-6 tumouroids were analyzed.

Supplementary tables

Table S1: Characteristics of $APC^{\Delta hep}$ tumours used for tumouroid generation and transcriptomics

Name	Type	Cumulative tumour diameter (mm) measured by ultrasound at sacrifice	Tumouroid number
TUM#1	HCC	12 * 11<br>hemorrhagic and necrotic areas	30
TUM#2	HCC	11 * 10	46
TUM#4G	HCC	6 * 6	41
TUM#4B	HCC	6 * 5	35
TUM#5	HB-like	10 * 8.5	32
TUM#7	HCC	8.5 * 8	28
TUM#9	HB-like	7 * 6.5	38
TUM#14	HB-like	12 * 10	63
TUM1#5	HB-like	11 * 10	75

Following tumour excision, one fragment was processed for histological analyses, a second fragment (~20 mg) was snap-frozen and stored at -80°C for RNA extraction, and the remaining tissue was used for tumouroid generation.

Table S2: Murine gene signatures generated for GSEA.

Human gene signature	Gene number	Reference	Murine gene list	Gene number
13_GENE_MBGS	13	43	*Slc1a2, Notum, Slc13a3, Lgr5, Axin2, Nkd1, Tcf7, Sp5, Rhbg, Glul, Tnfrsf19, Teddm1, Sbspon*	13
BOYAULT_LIVER_CANCER_SUBCLASS_G6_UP	66	40	*Acsl6, Acss3, Actn2, Ap3s1, Aspscr1, Atp6v0e2, Bcap31, Bmp4, Bpnt2, C1qtnf3, Cbr1, Ccdc170, Cemip2, Col7a1, Cryz, Ctnna2, Ctnnbl1, Dync1i1, Efr3a, Fbxl5, Fhit, Galnt1, Ggh, Greb1, Hexa, Hsdl2, Itpr2, Lef1, Lgals8, Lpin1, Mertk, Mlec, Mme, Nek3, Npr2, Panx1, Parvb, Preb, Rab4a, Rhobtb1, Rnf43, Samm50, Septin4, Siah2, Slc13a3, Slc22a4, Slc5a6, Slc6a2, Sult4a1, Tbca, Tceal1, Thsd1, Timm17a, Tmcc1, Trib2, Ttc9, Uggt1, Vsnl1, Vstm4, Washc3, Ypel1, Zbtb18*	62
LACHENMAYER Wnt-CTNNB1	17	42	*Nkd1, Axin2, Rock2, Sall1, Tle1, Dvl2, Ctnnbip1, Smad3, Tcf7, Brd7, Daam1, Cul1, Ppp3cb, Dlg1, Ruvbl1, Tbl1xr1, Senp2*	17
CHIANG_LIVER_CANCER_SUBCLASS_CTNNB1_UP	181	41	*Aaadac, Abcb11, Abcg2, Abhd6, Ace2, Acsl5, Acsl6, Acsf3, Actn2, Adh6a, Adrb2, Aldh1l1, Aldh3a1, Aldh3a2, Amacr, Ankfn1, Aox1, Aqp11, Aqp6, Aqp9, Ar, Asap2, Aspscr1, Axin2, Bambi, Bhlhe40, Bik, Bmp4, Bok, Cap2, Cavin2, Ccdc170, Cd36, Cdc14b, Cdk6, Cldn2, Corin, Cpped1, Crls1, Cst1, Ctnna2, Ctnnbl1, Cyp1a1, Cyp2e1, Cyp8b1, Dnajc12, Dpp4, Dsg1a, Ebpl, Ecm2, Ephb2, Esrrg, Exph5, Fam169a, Fam3b, Fam8a1, Fas, Fgf13, Fitm2, Frmd3, Gfra1, Glul, Glyat, Gnai1, Gpam, Gphn, Grhpr, Grk3, Gstm2, Habp4, Hepacam, Hhat, Hibadh, Hlf, Hoga1, Hpd, Hsd11b1, Hsdl2, Htr2b, Ift70a, Insig2, Irs1, Irx3, Itpr2, Kcnj8, Kcnk1, Lgr5, Map3k8, Mertk, Mme, Mthfd1, Myrip, Nags, Nek3, Nkd1, Nubpl, Nudt6, Panx1, Pdk1, Pdk4,*	177

			Phlpp1, Plaat2, Plppr1, Prag1, Preb, Prr5l, Ptprg, Rab11fip2, Rbp1, Reg1a, Reg3a, Rhbg, Rhobtb1, Rtp3, Sall1, Sec14l1, Selenbp1, Septin4, Shld2, Slc13a3, Slc16a1, Slc16a10, Slc16a11, Slc16a4, Slc17a1, Slc1a2, Slc22a11, Slc22a4, Slc25a30, Slc2a12, Slc47a1, Slc4a4, Slc5a6, Slc6a12, Slco1b2, Smpx, Snai2, Sparcl1, Spryd7, Srd5a2, Sult1b1, Tbc1d15, Tbck, Tbx3, Tenm2, Thbs4, Tmem100, Tmem150c, Tmem245, Tmem64, Tnfrsf19, Tprg1, Trib2, Tspan5, Ttc9, Ttpa, Ubxn10, Ust, Vegfd, Vldlr, Washc3, Zfp385b, Znrf3	
CAIRO_HEPATOBLASTOMA_UP	204	10	*Acaca, Acly, Acss3, Akr1c3, Alcam, Angel2, Ankrd27, Arl4a, Asap2, Aspscr1, Avl9, Bambi, Bcam, Bex1, Brd3, Camsap2, Cap2, Cbx1, Cbx5, Cbx6, Cct3, Cd24, Cd24p2, Cd46, Cdc23, Cdk4, Cep68, Cetn2, Ckap4, Ckb, Clic1, Clmn, Col15a1, Coq8a, Cpd, Creb3l2, Crim1, Cripto, Cs, Cyp51a1, Dcp2, Ddhd2, Denr, Dkk1, Dlg5, Dlk1, Dram1, Dzip3, Ehmt2, Elp1, Emcn, Epcam, Erg28, Etv1, Fasn, Fdft1, Fdps, Fkbp1b, Fnbp1l, Frrs1l, Fxr1, Gabbr1, Gja1, Glul, Gmnn, Gnpat, Gorasp2, Gpc3, Greb1, Gsta4, Hdac2, Heatr1, Hip1, Hmga2, Hmgcr, Hmgcs1, Hmgn4, Hsp90ab1, Hspa2, Hspb1, Igf2bp2, Ilf2, Impdh2, Itga6, Itgae, Jarid2, Kdm3b, Kdm5b, Khdc4, Lamc1, Lef1, Lgr5, Lrrc1, Lyz, Maged1, Maged2, Map3k4, Map4k4, Marchf8, Marcks, Mavs, Mcam, Mcm3, Meg3, Mff, Minpp1, Mlec, Mmp11, Mpzl1, Mrps27, Mtr, Nap1l1, Ncoa3, Ndn, Nsdhl, Nudt3, Nup133, Nup37, Obsl1, Palld, Papss1, Pdcd2, Pdgfa, Pdxk, Pdzk1, Peg10, Peg3, Pfkm, Piezo2, Plag1, Plppr1, Plvap, Plxnc1, Pms1, Podxl, Polr1d, Pomk, Ppp1cc, Ppp1r2, Prkca, Prkd1, Prr15l, Prune1, Psmd10, Psmd4, R3hdm1, Rab4a, Racgap1,*	204

			Rangrf, Rap2a, Rbm12, Rbm34, Rc3h2, Rcor3, Reln, Rhobtb1, Rnf38, Rnf43, Robo1, Rock2, Rpa1, Rps6kc1, Rrp1b, Sac3d1, Scd, Sephs1, Septin2, Septin8, Serpine2, Slc1a4, Slc25a36, Slc26a2, Slf2, Smarca4, Smarcc1, Smyd2, Smyd3, Snhg32, Socs5, Sox4, Sp3, Sqle, Srebf2, Suco, Tacc1, Tarbp1, Tax1bp3, Tbc1d9, Tbce, Tbx3, Tia1, Tmem243, Tnks, Tomm20, Tor3a, Tp53, Trib2, Tsn, Tspyl4, Tuft1, Tulp4, Ubap2, Ubap2l, Uggt1, Unc119b, Utrn, Uxs1, Vps45, Vwf, Wfs1, Zbtb38, Zfta, Znf189	
CAIRO_HEPATOBLASTOMA_DN	286	10	*Aass, Abat, Abcb11, Abhd2, Ablim3, Acaa1a, Acaa2, Acacb, Acadl, Acadm, Acads, Acat1, Acsl1, Adgra3, Adh1, Adk, Adm, Agl, Agpat2, Agxt, Akr1d1, Alas1, Aldob, Alpl, Ang, Angptl4, Ano1, Anxa10, Apof, Aqp3, Arhgef26, Asl, Ass1, Azgp1, Bbox1, Bmerb1, C1r, C1rl, C6, C8a, C8b, C8g, C9, Ca2, Cbs, Cd14, Cda, Cdc37l1, Cfp, Chst7, Cideb, Clec1b, Clec4m, Cpeb3, Cpt2, Crem, Crhbp, Crp, Cth, Ctps1, Cxcl14, Cyp1a2, Cyp26a1, Cyp39a1, Cyp3a13, Cyp4f14, Dao, Dcps, Ddt, Dhodh, Dhrs1, Dhtkd1, Dnase1l3, Ecm1, Ehd3, Endog, Eno3, Epha2, Etfdh, Etnppl, Ets2, Ext1, F11, F9, Faah, Fahd2a, Fam149a, Fbp1, Fcgr2b, Fetub, Foxo1, Fxyd1, Gadd45g, Gba3, Gcdh, Gcgr, Gch1, Ghr, Gls2, Glyat, Gne, Gnmt, Got1, Gpd1, Gpld1, Gpr88, Gramdc1, Gramdc4, Grem2, Gstt2, Gstz1, Gys2, Haao, Habp2, Hagh, Hal, Hao2, Hgd, Hgfac, Hp, Hpgd, Hpx, Hrg, Hsd17b6, Id2, Igf1, Igfals, Igfbp3, Il13ra2, Il1rap, Il4r, Inhbb, Inpp1, Insig1, Itih4, Kcnd3, Kdm8, Klf6, Kmo, Lcat, Lepr, Lonp2, Lpa, Lpin2, Lyve1, Man1c1, Map2k3, Map3k14, Map3k5, Marco, Masp2, Mat1a, Mcl1, Mfap3l, Mgll, Mmut, Mpc1, Mt1,*	273

			Myom1, N4bp2l1, Nampt, Nat2, Nfil3, Nnmt, Nnt, Nqo2, Nr1i2, Nr1i3, Nrg1, Ogdhl, Osbpl1a, Papss2, Pck1, Pck2, Pdlim5, Pemt, Pfkfb1, Plaat3, Plg, Pon1, Ppargc1a, Ppp1r1a, Prg4, Prodh, Pth1r, Pts, Pxdc1, Pxmp2, Pzp, Rab27a, Rcl1, Rdh16, Retreg1, Rida, Rnd3, Rnf125, Saa4, Sardh, Scp2, Sdhb, Sds, Serpinb8, Serpine1, Serping1, Shbg, Shmt1, Sigirr, Skap1, Slc10a1, Slc1a1, Slc20a1, Slc22a1, Slc22a7, Slc25a15, Slc27a2, Slc27a5, Slc37a4, Slc38a4, Slc3a1, Slc43a3, Slc46a3, Slpi, Socs2, Sorl1, Srd5a1, Srd5a2, St3gal6, Stab1, Stab2, Stard5, Suclg2, Syt17, Tdo2, Tenm1, Tent5a, Tfr2, Tmem131l, Tmprss2, Trib1, Tsku, Tspyl5, Ugp2, Ugt1a8, Vipr1, Vnn1, Vsig4, Xdh, Zc3h12a, Zfand5, Zg16, Zgpat	
CARRILLOREIXACH_HEPATOBLASTOMA_VS_NORMAL_HYPOMETHYLATED_AND_UP	162	44	*Acsl4, Adam9, Angpt1, Antxr1, Anxa1, Arid3a, Asap2, Axin2, B3galt2, Bambi, Bcam, Bcat1, Bcl9, Blmh, Bmp4, Cacnb4, Cd34, Cdh11, Cdh13, Cdk1, Cdk4, Cdk6, Cdkn3, Chd3, Chst11, Ckap2l, Clic1, Col12a1, Col15a1, Col1a1, Col1a2, Col2a1, Col4a1, Col4a2, Col6a3, Cpa6, Creb3l2, Depdc1, Dhx9, Dlgap5, Dnmt3a, Dpep1, Dtl, Dync1h1, Edil3, Esm1, F13a1, Fap, Fbln1, Fmnl2, Foxm1, Gas2l3, Gls, Gna12, Gramd1a, Greb1, Grk3, Hip1, Hk2, Hmgb2, Hnrnpa3, Hspg2, Htr1d, Hunk, Igf2bp1, Igf2bp2, Igsf1, Ilf3, Inpp5d, Itga2, Itga8, Kcnu1, Kif18a, Kit, Lama4, Lamb1, Lamc1, Lgr5, Map1b, Map4k4, Mctp1, Mgat5, Mgp, Mme, Mmp16, Ms4a2, Mtbp, Myo1d, Myo5a, Ncapd2, Ncapg, Ncaph, Nkd1, Notch3, Npm1, Nrp2, Nt5dc2, Nuak1, Nup93, Odam, Olfml2b, Olr1, Parpbp, Pdgfrb, Phyhipl, Piezo2, Plcg1, Pld3, Plxnc1, Prc1, Prkaa2, Prkdc, Ptk7, Ptpn14, R3hdm1, Racgap1, Rap2a, Rasgrf2, Rere, Rhobtb1, Rnas*	161

			e1, Robo1, Rpl8, Rps12, Rps3a, Rrm2, Sema3c, Sgo1, Slc1a5, Slc38a11, Slc7a11, Slc7a6, Slit3, Smarcc1, Smoc2, Sntg1, Spp1, Spry1, Stc1, Stk39, Sulf1, Tacc1, Tbx3, Tcf4, Tgfb2, Thbs2, Tmc7, Tnc, Tnfrsf19, Trib2, Trim71, Trps1, Tspan13, Txnip, Uaca, Uggt1, Vcan, Vwf, Zc3hav1l, Znrf3, Zswim5	
CARRILLOREIXACH_HEPATOBLASTOMA_VS_NORMAL_HYPERMETHYLATED_AND_DN	21	44	*A1bg, Acsl1, Adamtsl3, Akr7a3, Akr7l, Clybl, Ctps1, Cyp1a1, Cyp7b1, Ednrb, Esrp2, Hal, Hjv, Mt1l, Pemt, Per3, Plek2, Rhod, Slc43a3, Tbx15, Tspyl5*	21
WU_MESENCHYMAL_SPECIFIC_HEPATOBLASTOMA		28	*Sparc, Mef2c, Bgn, Col3a1, Timp2, Col5a1, Pdgfra, Col1a1, Dcn, Dnm3os, Cd9, Col1a2, Anxa1, Mgp, Igfbp7, Ifi6, Plxdc2, Eln, Itm2b, Calm1, Pls3, Nfic, Cybrd1, Timp3, Ano1, Fxyd6, Ahnak, Cd81, Eng, Col6a1, Egr1, Zcchc24, Junb, Vim, Col12a1, Anxa5, Ppp1r12b, Ids, Lpar1, Aebp1, Tagln, Emilin1, Kalrn, C1r, Mmp14, Ecm1, Lmna, Fstl1, Col8a1, S100a6*	50
WU_EMBRYONAL_SPECIFIC_HEPATOBLASTOMA		28	*Ambn, Mdfi, Mex3a, Vcan, Mmp16, Dach1, Sox4, Tyro3, Fbln2, Lnpep, Ibsp, Nap1l1, Rcn2, Tpbg, Gnas, Cxcl14, Nptx1, Laptm4b, Crabp2, Fkbp10, Tead2, Ptch1, Postn, Marcksl1, Sohlh2, Msx1, Frem2, Hnrnpa2b1, Ilf3, Ckap4, Cpxm1, Mycn, Phf6, Xrcc5, Hnrnpu, Slc34a2, Dbn1, Gpr161, Birc5, Tp53bp1, Wls, Fam84a, Axin2, Elmo1, Rasl11b, Vangl2, Hdac2, Hmga2*	49
SONG_FIBROBLAST_LIKE HEPATOBLASTOMA	145	27	*Col3a1, Col1a2, Col1a1, Postn, Cxcl14, Lum, Sparc, Dcn, Col6a3, Igfbp5, Fstl1, Col5a2, Prrx1, Col6a2, Ccdc80, Col14a1, Ogn, Ptn, Mgp, Ctsk, Igfbp3, Fbln1, Col6a1, Sfrp2, Aspn, Col5a1, Col12a1, Dpt, Emp1, Col16a1, Fbn1, Pcolce, Col2a1, Aebp1, Nnmt, Mtrnr2l1, Htra1, Igf1, Ltbp1, Prss23, Adamts1, Col11a1, Rnd3, Ptrf, Antxr1, Frzb, Lama4, Vwf, Angptl1, Cpe, Thbs2, Ppap2b, Cald1, Cybrd1, Timp3, Lamb1, Ctgf, Igfbp7, Defa3,*	142

			Crispld2, Wnt5a, Lgals1, Lama2, Spon1, Wwtr1, Tcf4, Il11ra, Palld, Timp2, Eln, Akap12, Igfbp4, Col4a1, Vcan, Col4a2, Thy1, Cyr61, Mxra5, Mmp2, Ednra, Moxd1, Cdh11, Sept11, Lrrc17, Fbln2, Cav1, Egfl6, Nfib, Gsn, Olfml3, Adamts9, Vim, Pxdn, Arid5b, Cpxm1, Fgf7, Ifi16, Kctd12, Abi3bp, Inhba, Comp, Zfp36l1, Calu, Emilin1, Col7a1, Slit3, Lox, Gng11, Mmp14, Fam150b, Kcne4, Mmrn1, Mxra8, Fmod, Ppic, Nbl1, Lrrc15, Sh3pxd2a, Sfrp1, Adamts2, Rpl9, Pdgfrb, Sulf1, Gja1, Adam33, Adirf, Anxa2, Tm4sf1, Calcrl, C1r, Bicc1, Serpinh1, Sesn3, Fibin, Serpine1, Fkbp7, Ttc3, Spry1, Twist1, Sept2, Id3, C1qtnf5, Omd	

Table S3: P values for heatmap from Figure 5C

Gene name	HCC vs NT	Tumouroid vs NT	Tumouroid vs HCC
Nkd1	8.41E-79	9.43E-78	0.89
Axin2	2.24E-15	3.83E-14	0.71
Lgr5	1.32E-08	6.81E-08	0.77
SP5	3.83E-34	6.81E-32	0.65
Znrf3	2.29E-06	6.72E-06	0.82
Lef1	2.78E-12	2.44E-12	0.98
Notum	3.52E-13	3.15E-12	0.76
Rnf43	5.94E-08	3.54E-07	0.74
Tnfrsf19	7.97E-10	8.54E-10	0.99
Slc13a3	3.38E-36	2.15E-45	0.89
Trib2	5.23E-10	1.46E-09	0.86
Lect2	4.17E-08	1.65E-07	0.8
Oat	1.66E-07	1.51E-06	0.67
Tbx3	3.39E-05	8.71E-05	0.82
Slc1a2	7.02E-18	7.58E-17	0.78
Glul	6.26E-30	4.42E-29	0.86
Rhbg	1.19E-23	4.21E-23	0.89
Aldh3a2	1.94E-07	7.18E-07	0.8
Aox1	5.55E-09	4.15E-08	0.72
Amacr	6.00E-03	6.00E-03	0.98
Abcc12	1.00E-02	1.00E-02	0.98
Abcc4	1.17E-30	5.51E-31	0.94
Cyp2a5	4.64E-09	5.88E-09	0.96
Ccna2	2.80E-37	6.41E-37	0.93
Mki67	1.15E-30	4.02E-31	0.92
E2f1	2.15E-15	5.96E-17	0.66
Nek2	3.60E-39	2.39E-39	0.97
Cd34	8.24E-39	2.30E-08	0.85
Mcam	1.43E-08	1.52E-06	0.38
Nqo1	9.78E-15	3.84E-13	0.62
Gsta2	3.00E-03	5.00E-03	0.84
Prdx3	2.75E-12	1.20E-11	0.83
Cbr3	5.00E-03	2.19E-12	0.97
Gstm6	2.13E-07	7.24E-07	0.81
Gstm2	4.79E-16	6.07E-15	0.75
Srxn1	1.61E-17	2.05E-19	0.62
Ccl2	8.00E-04	3.90E-06	0.19
Cd14	9.00E-04	3.20E-06	0.17
Cd68	4.00E-03	4.50E-06	0.07
Il10rb	3.60E-09	4.50E-09	0.96
Gldc	1.01E-17	4.17E-15	0.46
Sdsl	5.42E-14	2.18E-11	0.33
Hal	4.88E-07	1.31E-07	0.8

Got1	3.41E-23	2.21E-22	0.89

Table S4: P values for heatmap from Figure 6C

Gene name	HB vs NT	Tumouroid vs NT	Tumouroid vs Tum
Nkd1	0.00E+00	0.00E+00	0.82
Axin2	0.00E+00	0.00E+00	0.6
SP5	1.20E-93	1.00E-91	0.82
Notum	3.51E-83	4.06E-82	0.89
Znrf3	1.24E-53	7.09E-52	0.78
Rnf43	6.41E-26	3.87E-28	0.63
Tnfrsf19	2.86E-72	3.04E-71	0.89
Lef1	5.71E-47	4.15E-46	0.88
Tead1	4.00E-04	4.00E-04	0.99
Tead2	2.00E-39	6.38E-37	0.62
Tead4	6.73E-16	1.21E-17	0.55
Ankrd12	4.00E-04	4.32E-05	0.56
Nuak1	1.54E-08	1.79E-08	0.97
Tgfb2	1.32E-38	5.38E-40	0.79
Adam12	5.99E-24	1.77E-27	0.4
Adam19	2.14E-08	2.58E-10	0.45
Col1a2	2.87E-06	4.05E-07	0.69
Col3a1	5.00E-04	1.00E-04	0.66
Sox4	6.71E-47	3.72E-49	0.71
Sfrp1	2.66E-27	5.21E-26	0.75
Rspo3	1.00E-03	1.00E-03	0.98
Dkk1	8.41E-14	6.64E-14	0.89
Vim	5.47E-34	5.20E-36	0.7
Mmp2	7.99E-19	5.21E-20	0.76
Zeb1	3.42E-18	1.36E-18	0.91
Zeb2	1.42E-08	6.80E-11	0.38
Cdh1	1.00E-03	7.00E-04	0.77
Cdh2	2.00E-03	3.00E-03	0.96
Mcam	3.40E-06	2.85E-05	0.64
CD34	6.95E-15	1.78E-15	0.85
s	1.11E-07	3.14E-07	0.84
Mki67	3.69E-10	8.25E-10	0.89
E2f1	2.71E-05	3.13E-05	0.97
CD163	3.44E-08	7.98E-11	0.55
Il10rB	5.00E-03	2.00E-03	0.8
Ccr2	9.00E-03	2.00E-02	0.77
Cxcl10	7.00E-03	1.00E-02	0.77
Cxcl11	5.64E-06	5.56E-06	0.99
