# Supplementary material for: Novel 3D models for β-catenin-driven liver cancers
Source: JHEP Rep. 2026 Sep 18;8(12):102040. doi: 10.1016/j.jhepr.2026.102040 (PMC13755961; doi:10.1016/j.jhepr.2026.102040)
Supplement: Supplementary file 4 [file mmc2.docx]

**JHEP Reports**

**CTAT methods**

Tables for a “Complete, Transparent, Accurate and Timely account” (CTAT) are now mandatory for all revised submissions. The aim is to enhance the reproducibility of methods.

- Only include the parts relevant to your study
- Refer to the CTAT in the main text as ‘Supplementary CTAT Table’
- Do not add subheadings
- Add as many rows as needed to include all information
- Only include one item per row
  1.1 **Antibodies**

| **Name** | **Supplier** | **Reference** | **Dilution** | **Application** |
| --- | --- | --- | --- | --- |
| Cyclin A2 | Abcam | AB-32386 | 1:100 | Comet |
| CD146 | Cell Signaling | 81701 | 1:50, 1:100 | Comet / IHC |
| F4/80 | Cell Signaling | 70076 | 1:100 | Comet / IHC |
| β-catenin | Abcam | AB-224803 | 1:200 | Comet |
| Vimentin | Abcam | AB-92547 | 1:1000 | Comet |
| Glutamine synthetase | BD Biosciences | 610518 | 1:2000 / 1:1000 | Comet / IHC |
| Anti-Rabbit AF555 | ThermoFisher Scientific | A31572 | 1:100 | Comet |
| Anti-Rabbit AF647 | ThermoFisher Scientific | A21245 | 1:200 | Comet |
| Anti-Mouse AF647 | ThermoFisher Scientific | A21236 | 1:200 | Comet |
| Anti-Rat AF647 | ThermoFisher Scientific | A21247 | 1:200 | Comet |
| Anti-Goat 647 | ThermoFisher Scientific | A21446 | 1:200 | Comet |
| Ki67 | ThermoFisher Scientific | MA5-14520 | 1:200 | IHC |
| Cleaved caspase-3 | Cell signaling | D175 | 1:600 | IHC |
| Carbonic anhydrase 9 | Abcam | AB-243660 | 1:2000 | IHC |

1.2 **Cell lines**

| **Name** | **Citation** | **Supplier** | **Cat no.** | **Passage no.** | **Authentication test method** |
| --- | --- | --- | --- | --- | --- |
|  |  |  |  |  |  |

1.3 **Organisms**

| **Name** | **Citation** | **Supplier** | **Strain** | **Sex** | **Age** | **Overall n number** |
| --- | --- | --- | --- | --- | --- | --- |
| Mus musculus | PMID: **35257829** | Janvier | C57Bl6J | Males | 2 months | **4** |
| APC^lox/lox^ mice | PMID: 15563600 | Sabine Colnot | C57Bl6J | Males | 2 months | **17** |
| APC^lox/lox^/ CRE^ERT2^ mice | PMID: 15563600 | Sabine Colnot | C57Bl6J | Males | 2 months | **10** |

1.4 **Sequence based reagents**

**List of primers and probes for QPCR**

| **Name** | **Reference/Sequence** | **Supplier** |
| --- | --- | --- |
| *Apc*  *(total transcript)* | Mm00545877 | ThermoFisher Scientific |
| *Apc*  *(exon 14 transcript)* | Mm01130462 | ThermoFisher Scientific |
| *Axin2* | Mm00443610 | ThermoFisher Scientific |
| *Glul* | Mm00725701 | ThermoFisher Scientific |
| *Epo* | Mm01202755 | ThermoFisher Scientific |
| *Lgr5* | Mm00438890 | ThermoFisher Scientific |
| *Arg1* | Mm00475988 | ThermoFisher Scientific |
| *B2m* | F 5’-ACAGTTCCACCCGCCTCACATT-3’  R 5’-TAGAAAGACCAGTCCTTGCTGAAG-3’ | Eurogentec |
| *Noxa* | F 5’-GGAAGTCGCAAAAGAGCAGGATG-3’  R 5’-CTGCCGTAAATTCACTTTGTCTCC-3’ | Eurogentec |
| *Bcl2l1* | F 5’-CGACCCAGCCACCACCTCCTC-3’  R 5’-TGGGGACTCAGTCCTATTCTC-3’ | Eurogentec |
| *Mki67* | F 5’-CTGCCTGCGAAGAGAGCATC  R 5’-AGCTCCACTTCGCCTTTTGG | Eurogentec |
| *Scl13a3* | F 5’-GAAGTTGGTGTCGAGGAAGT  R 5’-GGAAGGCCGATGCCTCTATG | Eurogentec |
| *Epo* | F 5’-CACAACCCATCGTGACATTTT  R 5’-CATCTGCGACAGTCGAGTTCT | Eurogentec |
| *Vegfa* | F 5’-GGACATCTTCCAGGAGTACC  R 5’-TCCGCATAATCTGCATGGTG | Eurogentec |

1.5 **Biological samples**

| **Description** | **Source** | **Identifier** |
| --- | --- | --- |
|  |  |  |

1.6 **Deposited data**

| **Name of repository** | **Identifier** | **Link** |
| --- | --- | --- |
| European Nucleotide Archive (ENA) | PRJEB115263 |  |

1.7 **Software**

| **Software name** | **Manufacturer** | **Version** |
| --- | --- | --- |
| GraphPad Prism | GraphPad Software | 10 |
| MicrosoftExcel for PC | Microsoft | 2024 |
| Microsoft Power point for PC | Microsoft | 2024 |
| Microsoft Word for PC | Microsoft | 2024 |
| Biorender | Biorender |  |
| Fiji for PC | Image J | 2026 |
| Qupath | Qupath | 0.6.0 |
| Zeiss zen | Zeiss | 3.13 |

1.8 **Other (*e.g*. drugs, proteins, vectors etc.)**

| **Drug** | **Supplier** | **Reference** |
| --- | --- | --- |
| WNTinib | MedChemExpress | HY-160765 |
| trichostatin A | Selleck Chemicals | S1045 |
| sorafenib | Selleck Chemicals | S7397 |

1.9 **Please provide the details of the corresponding methods author for the manuscript:**

**Christèle Desbois-Mouthon e-mail:** [**christele.desbois-mouthon@inserm.fr**](mailto:christele.desbois-mouthon@inserm.fr) **and Vanessa Bou Malham, e-mail:** [**vanessa.bou-malham@inserm.fr**](mailto:vanessa.bou-malham@inserm.fr)

**2.0	Please confirm for randomised controlled trials all versions of the clinical protocol are included in the submission. These will be published online as supplementary information.**
